# Supplementary material for: Spatio-temporal model to estimate life expectancy and to detect unusual trends at the local authority level in England
Source: BMJ Open. 2020 Nov 12;10(11):e036855. doi: 10.1136/bmjopen-2020-036855 (PMC7662413; doi:10.1136/bmjopen-2020-036855)

Supplementary Material: A spatio-temporal model to estimate life expectancy and to detect unusual trends at the local authority level in England

Areti Boulieri[a,], Marta Blangiardo[a*]

[a] Department of Epidemiology and Biostatistics, MRC Centre for Environment and Health, Imperial College London, London, UK

[*] Corresponding author:
Marta Blangiardo
Imperial College London, St Mary's Campus,
Norfolk Place, W2 1PG
m.blangiardo@imperial.ac.uk

Table S1: *: Local authorities with a low life expectancy for their deprivation level for females under the model with deprivation index at 2010. The areas detected under the model with deprivation index at 2019 are a subset (in bold). We present the posterior probability under the two models.*

		IMD2010		IMD2019	
Local Authority	Year	Decile	Pr(ω_{it}>0.95)	Decile	Pr(ω_{it}>0.95)
Blackpool	2017	D1	0.976	D1	0.912
Blackpool	2018	D1	0.959	D1	0.871
Bradford	2013	D1	0.981	D1	0.941
Kingston upon Hull, City of	2014	D1	0.951	D1	0.881
Kingston upon Hull, City of	2015	D1	0.972	D1	0.914
**Kingston upon Hull, City of**	**2017**	**D1**	**0.997**	**D1**	**0.977**
Kingston upon Hull, City of	2018	D1	0.981	D1	0.915
Manchester	2014	D1	0.960	D1	0.894
Oldham	2014	D1	0.980	D1	0.949
Stoke-on-Trent	2017	D1	0.980	D1	0.913
**Stoke-on-Trent**	**2018**	**D1**	**1.000**	**D1**	**0.999**
**Tameside**	**2018**	**D1**	**0.993**	**D1**	**0.969**
Wolverhampton	2017	D1	0.961	D1	0.864
Wolverhampton	2018	D1	0.991	D1	0.941
**Leeds**	**2013**	**D3**	**0.999**	**D2**	**0.997**
**Leeds**	**2015**	**D3**	**0.981**	**D2**	**0.958**
**Leeds**	**2018**	**D3**	**1.000**	**D2**	**1.000**
**Newcastle upon Tyne**	**2015**	**D2**	**0.974**	**D2**	**0.951**
Mansfield	2017	D2	0.962	D2	0.919
**Plymouth**	**2017**	**D3**	**0.986**	**D2**	**0.970**
King's Lynn and West Norfolk	2017	D3	0.951	D3	0.922
Medway	2014	D3	0.952	D3	0.930
**Derby**	**2018**	**D4**	**0.954**	**D3**	**0.956**
**County Durham**	**2013**	**D3**	**0.989**	**D4**	**0.978**
Cornwall inc Isles of Scilly	2013	D3	0.956	D4	0.923
**Cornwall inc Isles of Scilly**	**2016**	**D3**	**0.974**	**D4**	**0.964**
North Tyneside	2017	D4	0.954	D4	0.925
Breckland	2015	D5	0.956	D5	0.912
Stockport	2013	D5	0.967	D5	0.942
**Bournemouth, Christchurch and Poole**	**2015**	**D3**	**0.998**	**D6**	**0.996**
Bournemouth, Christchurch and Poole	2016	D3	0.959	D6	0.941
**Bournemouth, Christchurch and Poole**	**2017**	**D3**	**0.975**	**D6**	**0.963**
Colchester	2014	D7	0.959	D6	0.925
Colchester	2017	D7	0.970	D6	0.939
East Riding of Yorkshire	2012	D7	0.967	D7	0.931

Epping Forest	2014	D7	0.955	D7	0.905
Dorset Council	2016	D7	0.982	D7	0.946
North Somerset	2015	D7	0.967	D7	0.920
Chelmsford	2017	D6	0.965	D8	0.908
**Sutton**	**2014**	**D6**	**0.974**	**D8**	**0.954**
Tonbridge and Malling	2018	D8	0.954	D8	0.889
**Stroud**	**2013**	**D8**	**0.977**	**D9**	**0.954**
Stroud	2017	D8	0.952	D9	0.932
Harrogate	2014	D9	0.951	D9	0.901

Table S2: *Local authorities with a low life expectancy for their deprivation level for males under the model with deprivation index at 2010. The areas detected under the model with deprivation index at 2019 are a subset (in bold). We present the posterior probability under the two models.*

		IMD2010		IMD2019	
Local Authority	Year	Decile	Pr(ω_{it}>0.95)	Decile	Pr(ω_{it}>0.95)
Middlesbrough	2014	D1	0.962	D1	0.944
**Middlesbrough**	**2017**	**D1**	**0.989**	**D1**	**0.976**
**Blackpool**	**2015**	**D1**	**0.993**	**D1**	**0.985**
Blackpool	2018	D1	0.952	D1	0.896
Kingston upon Hull, City of	2016	D1	0.955	D1	0.892
**Kingston upon Hull, City of**	**2017**	**D1**	**1.000**	**D1**	**0.998**
Leicester	2015	D1	0.953	D1	0.902
Leicester	2017	D1	0.960	D1	0.891
Manchester	2014	D1	0.970	D1	0.933
Stoke-on-Trent	2018	D1	0.974	D1	0.918
South Tyneside	2018	D2	0.967	D1	0.945
**Birmingham**	**2014**	**D1**	**1.000**	**D1**	**0.996**
Walsall	2015	D2	0.970	D1	0.933
Walsall	2017	D2	0.974	D1	0.920
**Wolverhampton**	**2018**	**D1**	**0.995**	**D1**	**0.978**
Bradford	2016	D1	0.965	D1	0.870
**Leeds**	**2015**	**D3**	**0.980**	**D2**	**0.985**
**Newcastle upon Tyne**	**2014**	**D2**	**0.967**	**D2**	**0.970**
**Southampton**	**2013**	**D3**	**0.974**	**D2**	**0.977**
**Tendring**	**2017**	**D3**	**0.961**	**D2**	**0.967**
**Bristol, City of**	**2013**	**D3**	**0.975**	**D3**	**0.985**
**Bristol, City of**	**2018**	**D3**	**0.977**	**D3**	**0.990**
**Cornwall inc Isles of Scilly**	**2018**	**D3**	**0.995**	**D4**	**0.993**
**Ashford**	**2018**	**D6**	**0.957**	**D5**	**0.958**
**Breckland**	**2018**	**D6**	**0.995**	**D5**	**0.995**
**Hillingdon**	**2018**	**D4**	**0.951**	**D5**	**0.959**
Cherwell	2014	D8	0.952	D7	0.942
**Dorset Council**	**2016**	**D7**	**0.963**	**D7**	**0.965**
**Teignbridge**	**2012**	**D6**	**0.965**	**D7**	**0.964**
**Stroud**	**2016**	**D8**	**0.975**	**D9**	**0.976**
**Bath and North East Somerset**	**2016**	**D9**	**0.966**	**D9**	**0.967**

Mortality rates for the detected areas under the main model and comparison against the corresponding decile (IMD2019), females

Figure S1: Kingston upon Hull, females

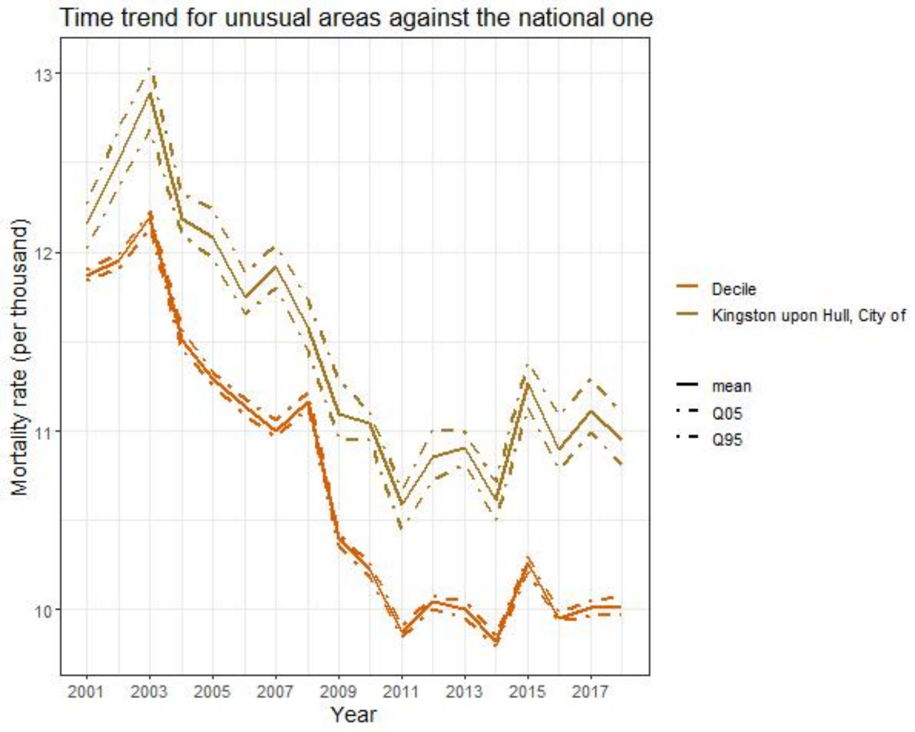

Figure S2: Stoke-on-Trent, females

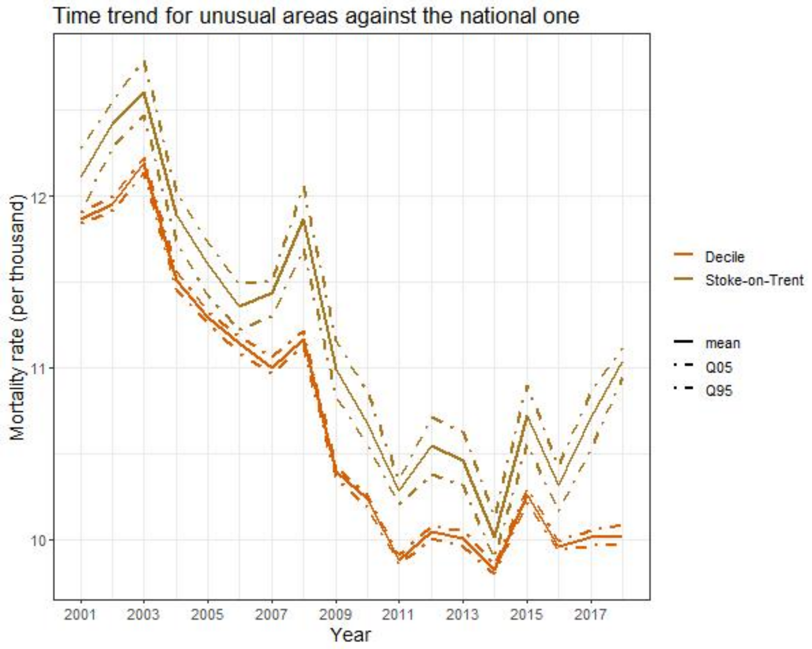

Figure S3: Tameside, females

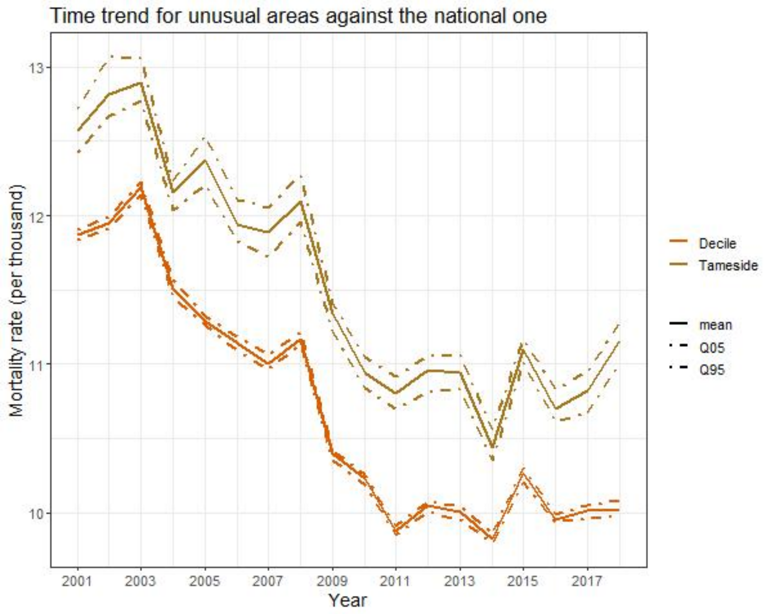

Figure S4: Newcastle upon Tyne, females

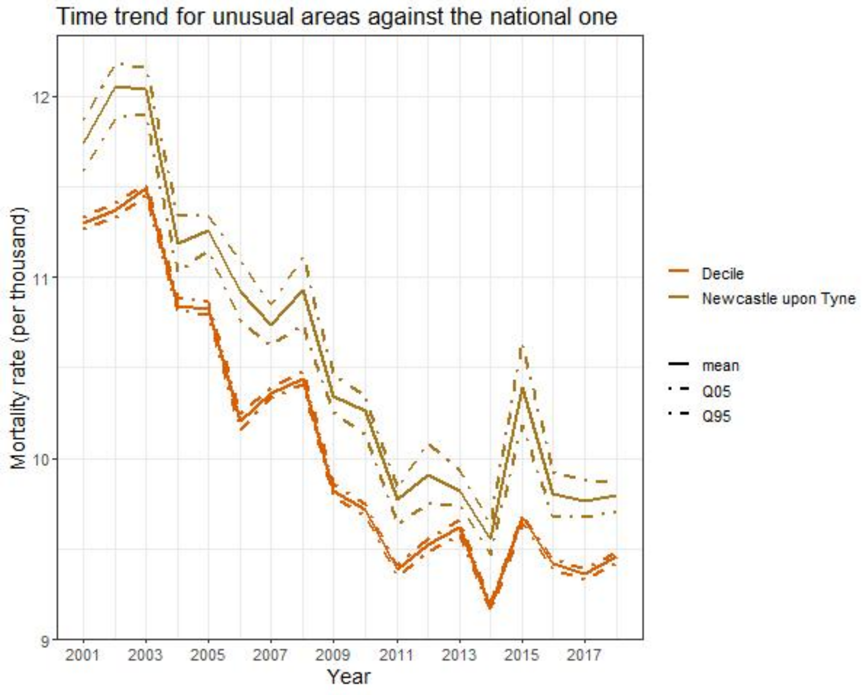

Figure S5: Plymouth, females

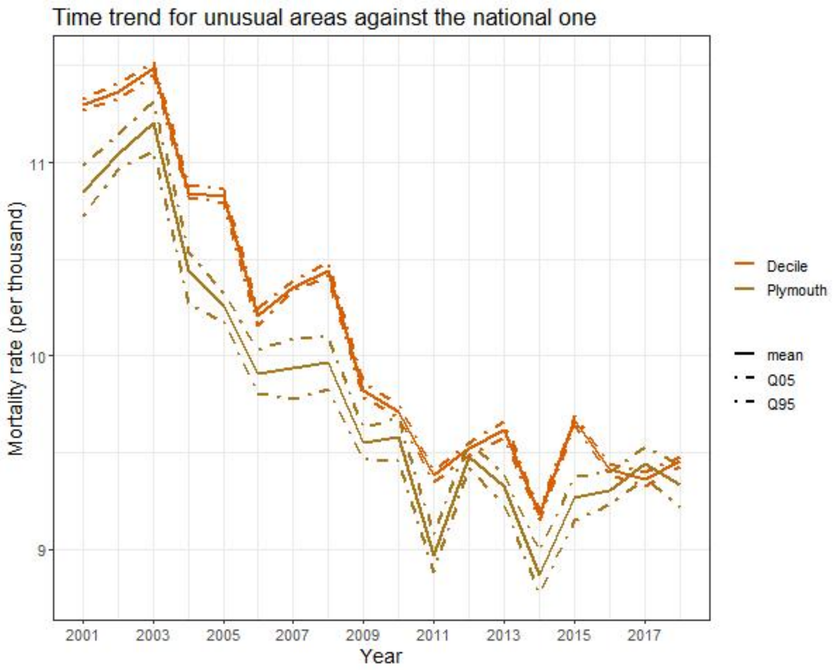

Figure S6: Leeds, females

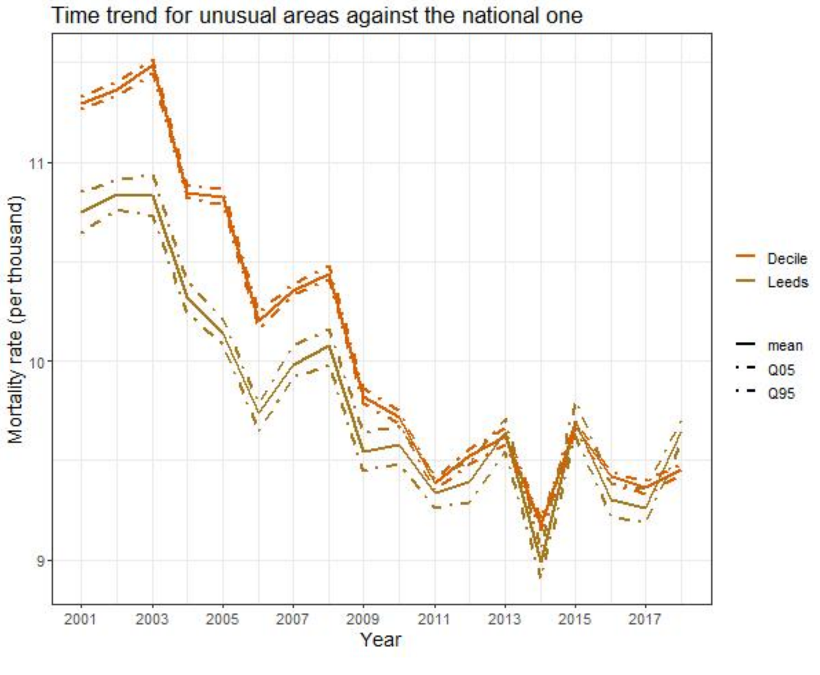

Figure S7: Derby, females

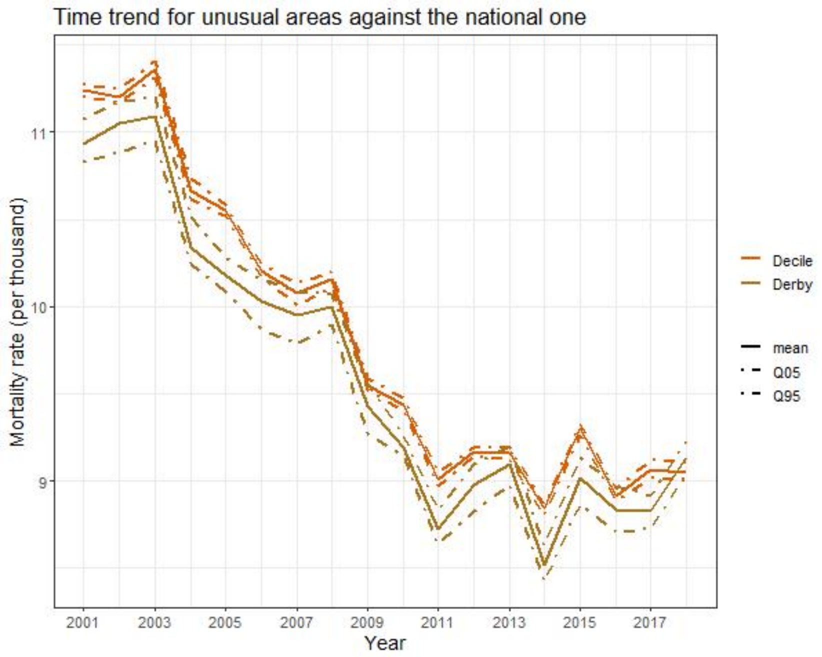

Figure S8: County Durham, females

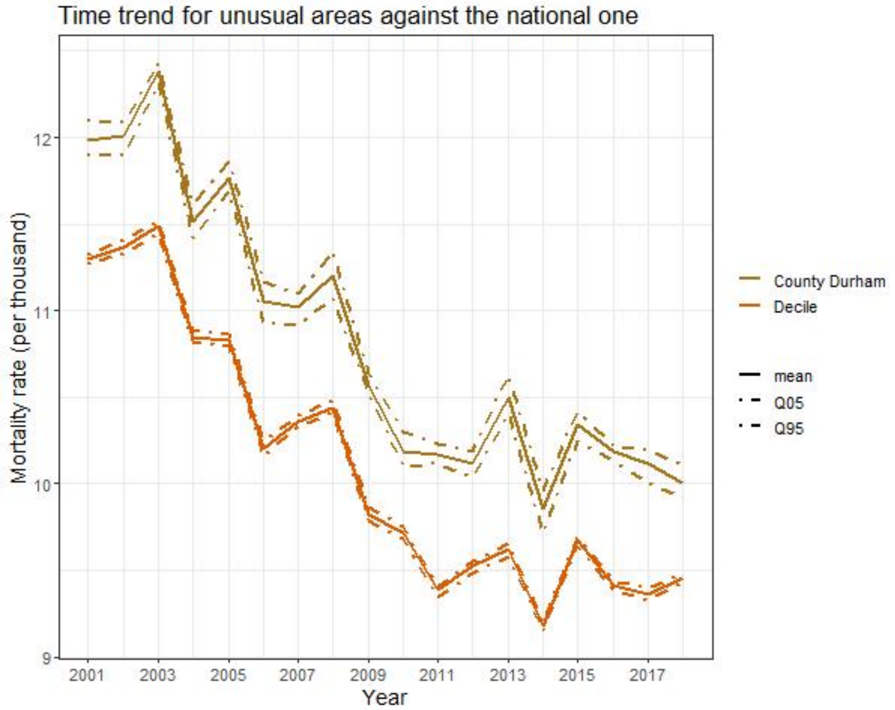

Figure S9: Cornwall including the Isles of Scilly, females

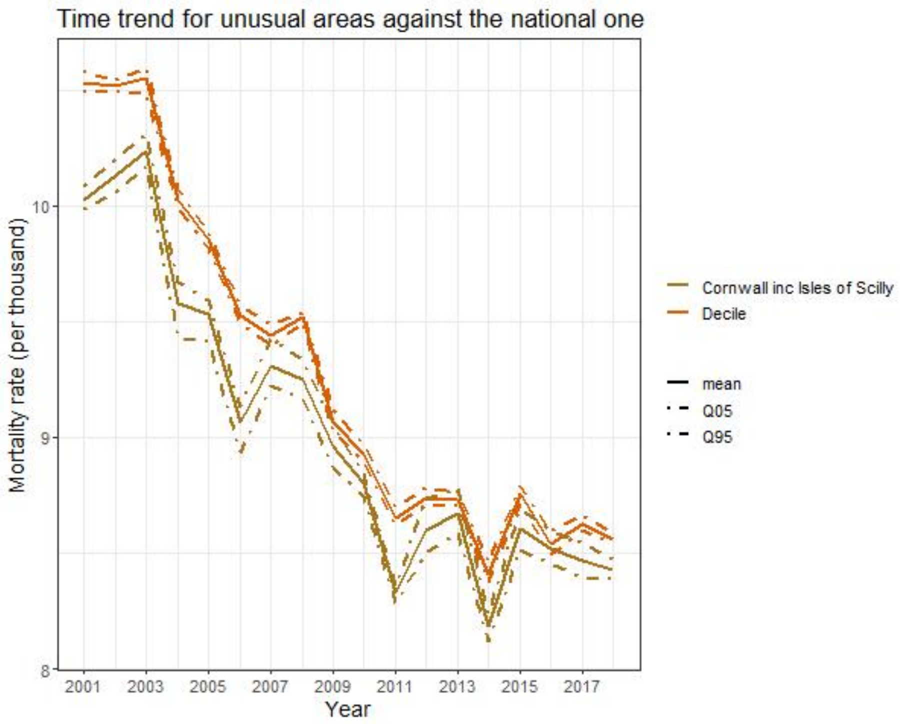

Figure S10: Bournemouth, Christchurch and Poole, Females

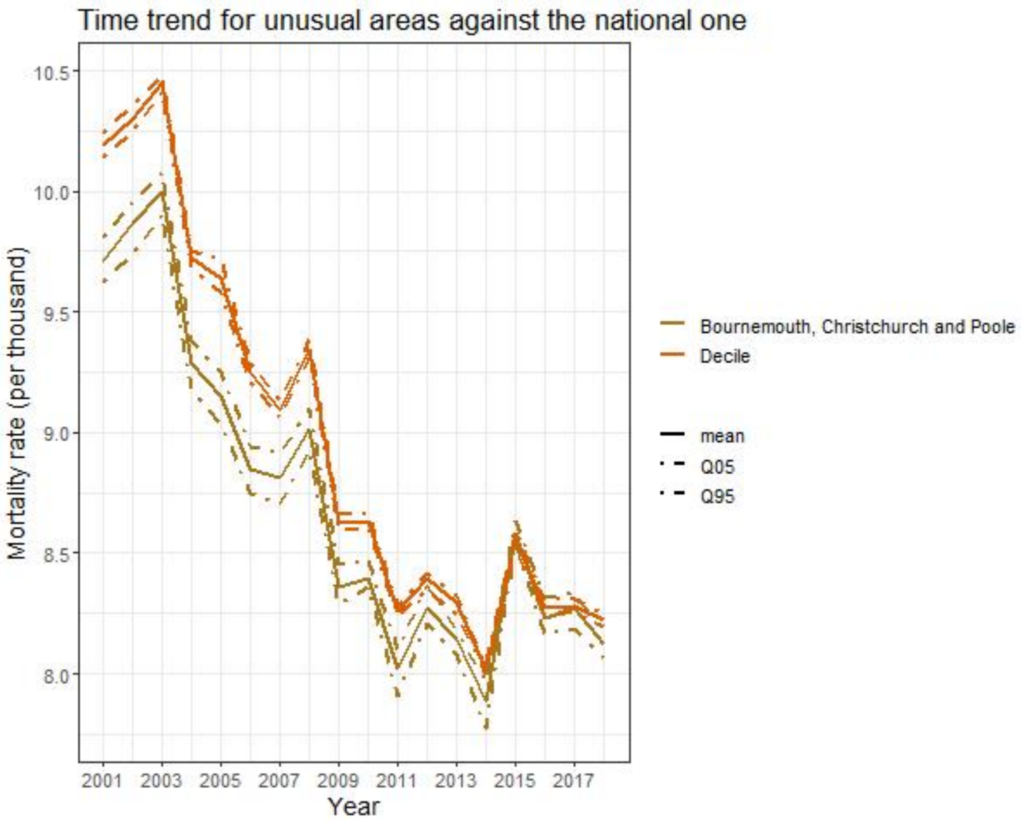

Figure S11: Stroud, females

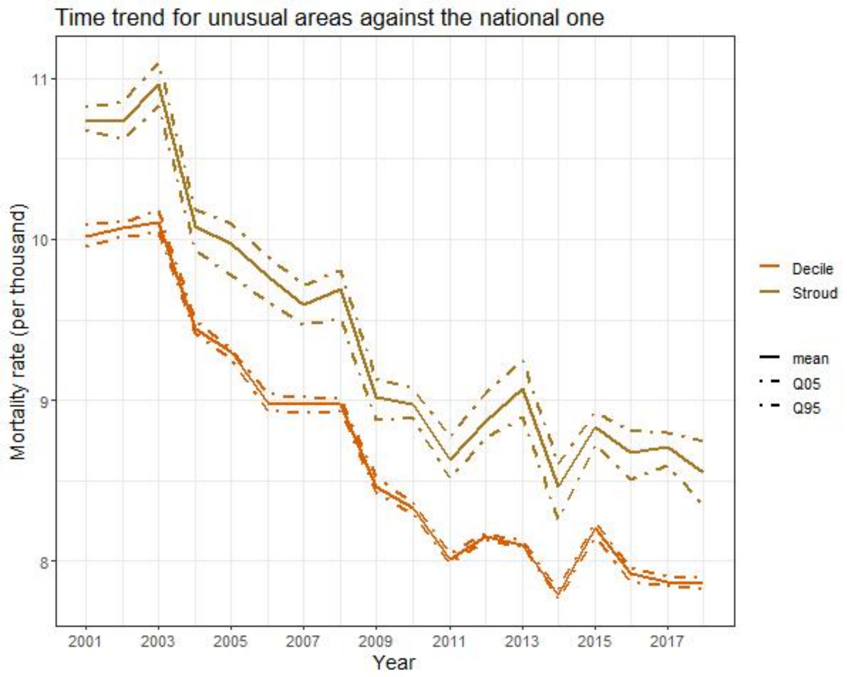

Figure S12: Sutton, females

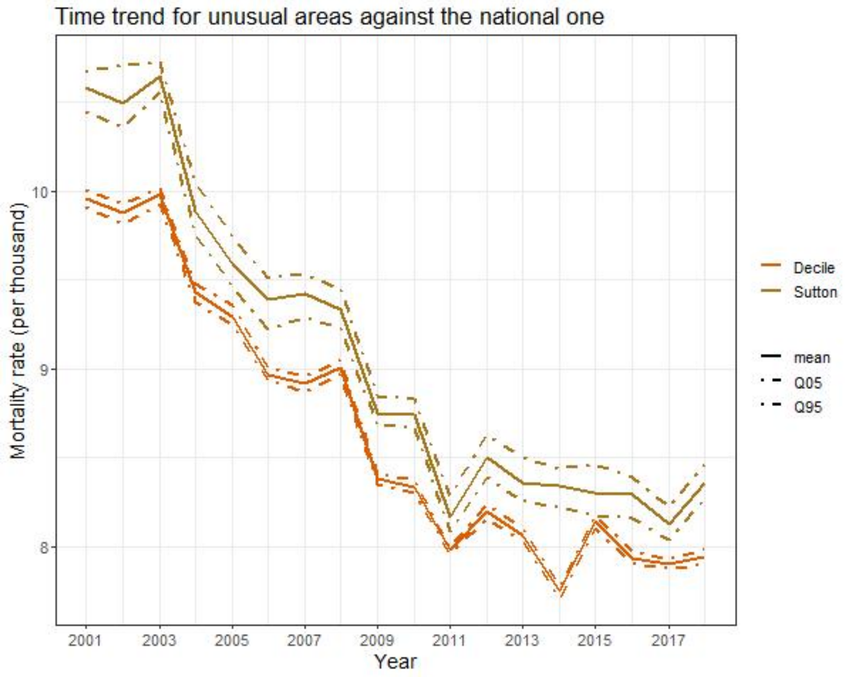

Mortality rates for the detected areas under the main model and comparison against the corresponding decile (IMD2019), males

Figure S13: Middlesbrough, males

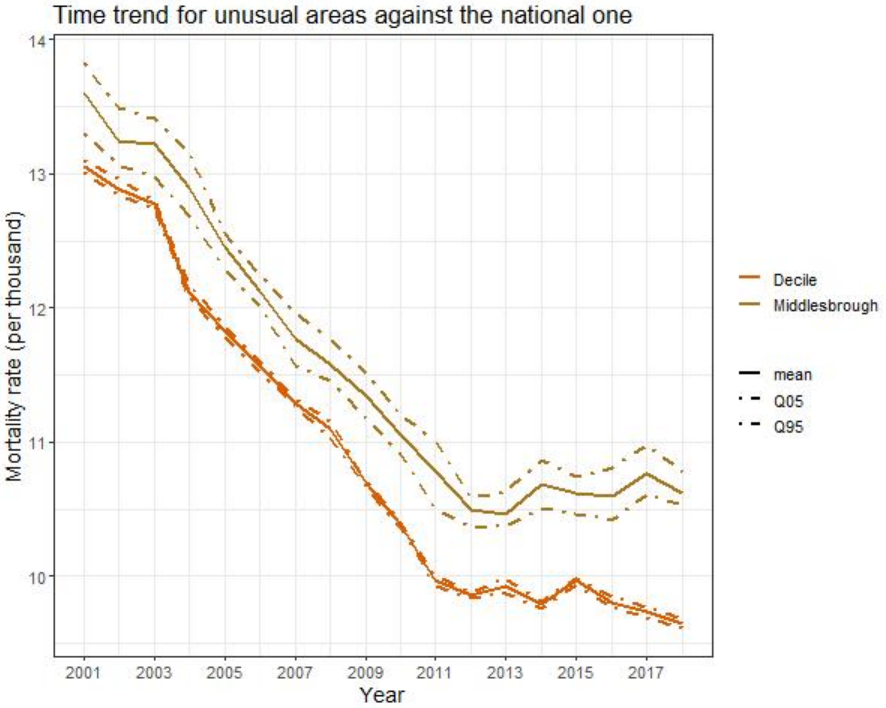

Figure S14: Wolverhampton, males

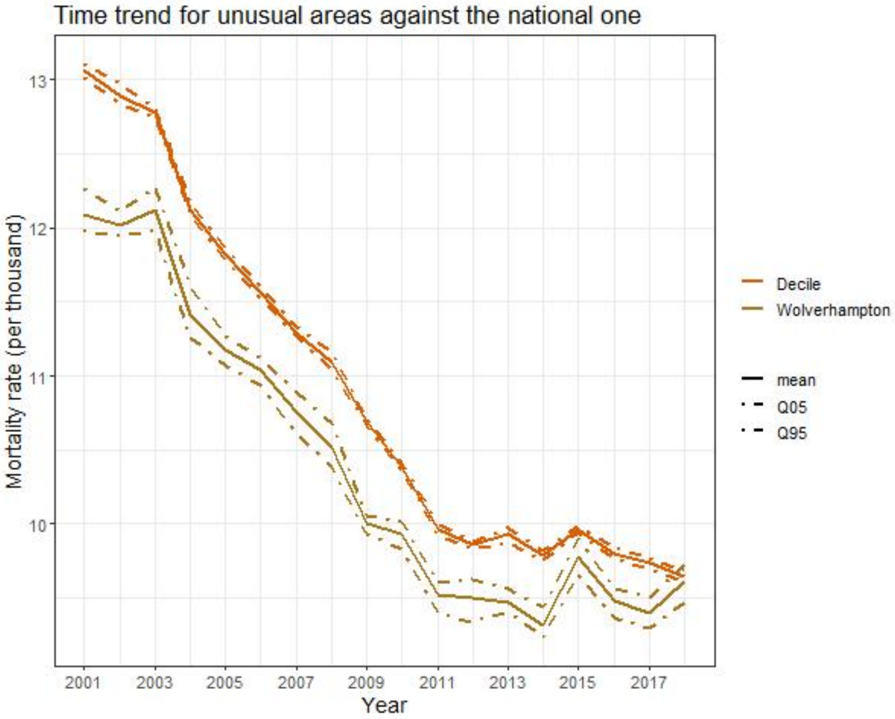

Figure S15: Kingston upon Hull, males

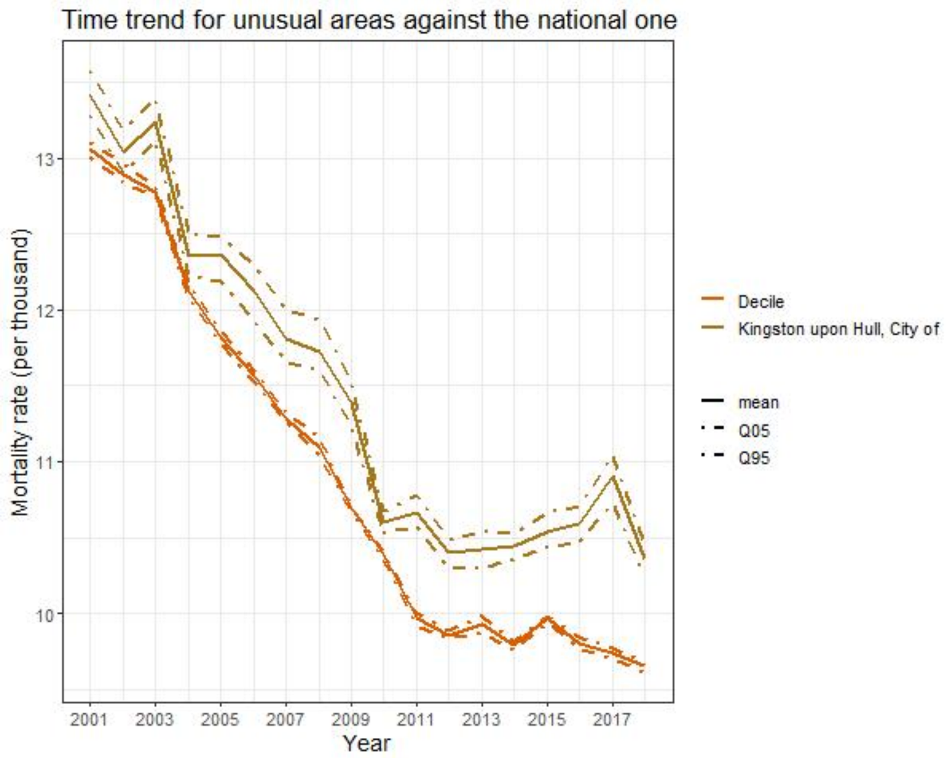

Figure S16: Birmingham, males

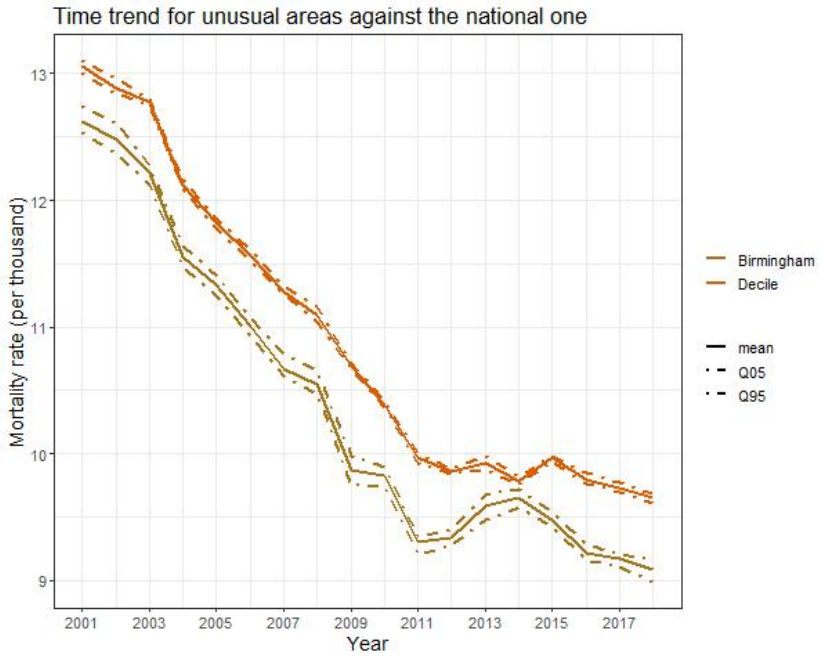

Figure S17: Tendring, males

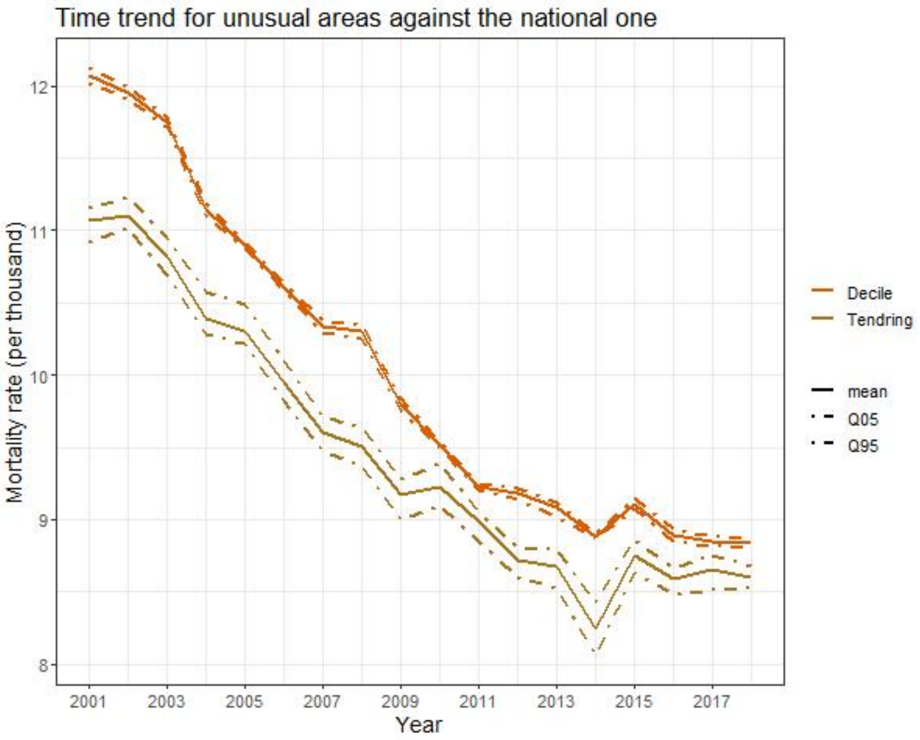

Figure S18: Leeds, males

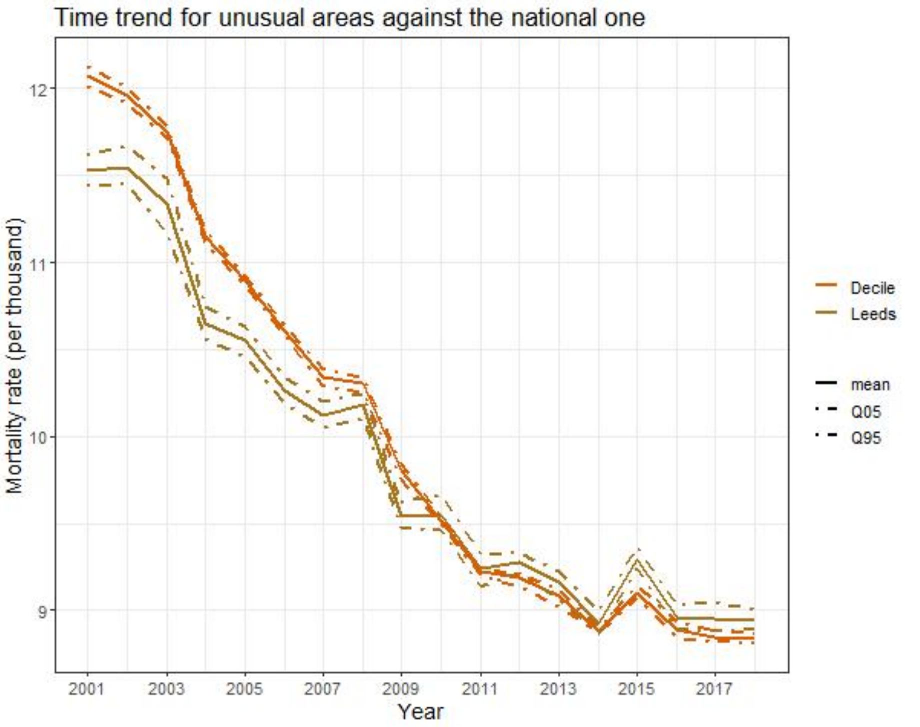

Figure S19: Newcastle upon Tyne, males

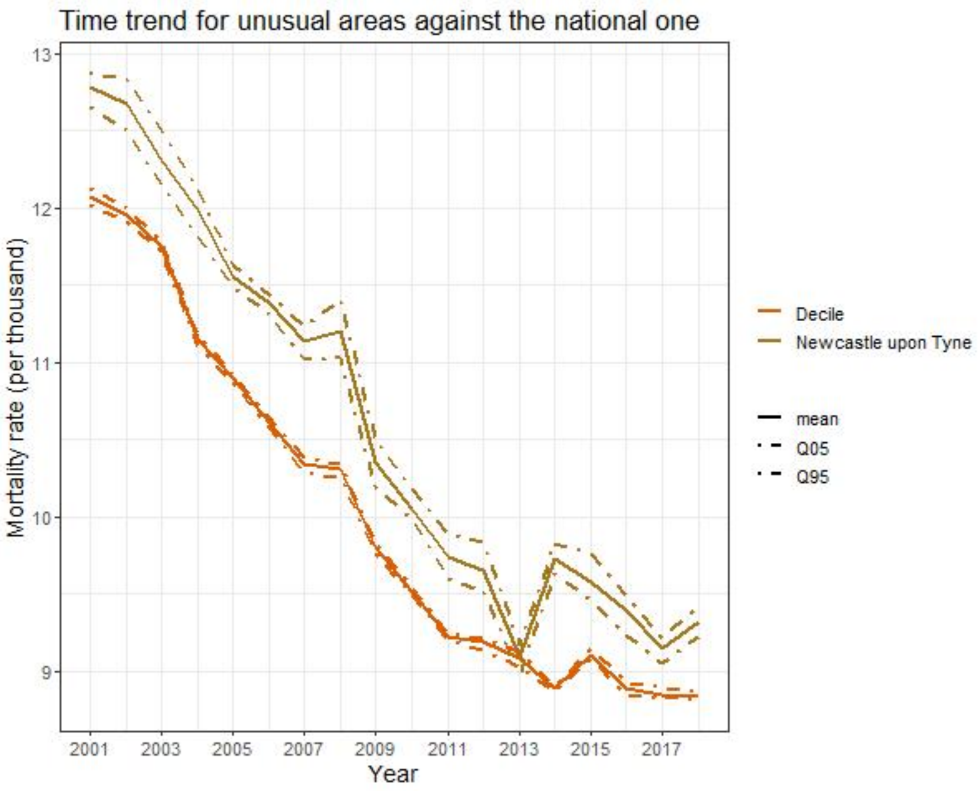

Figure S20: Southampton, males

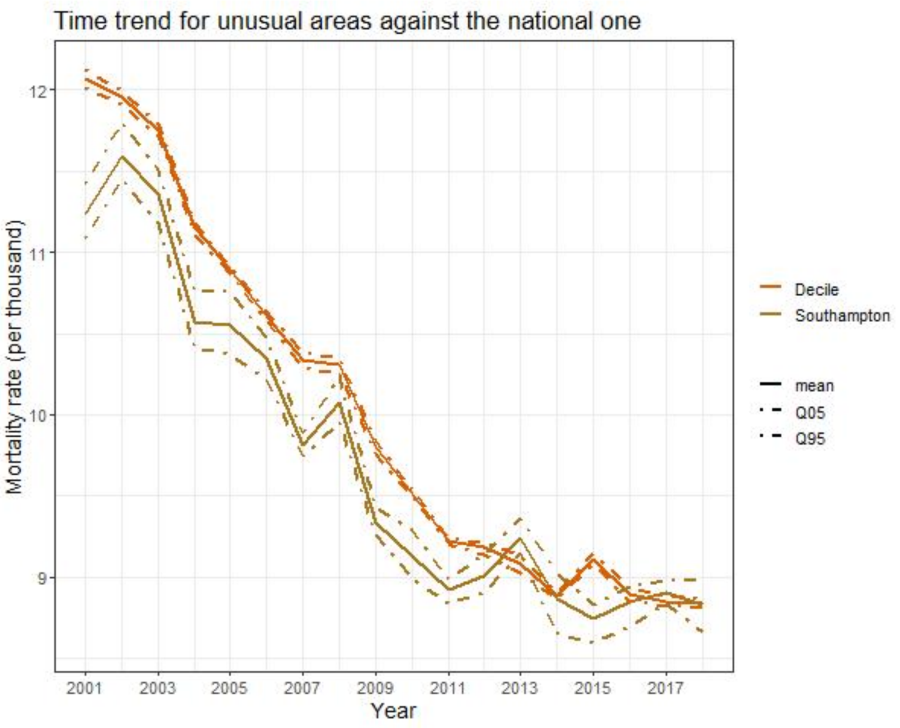

Figure S21: Bristol, males

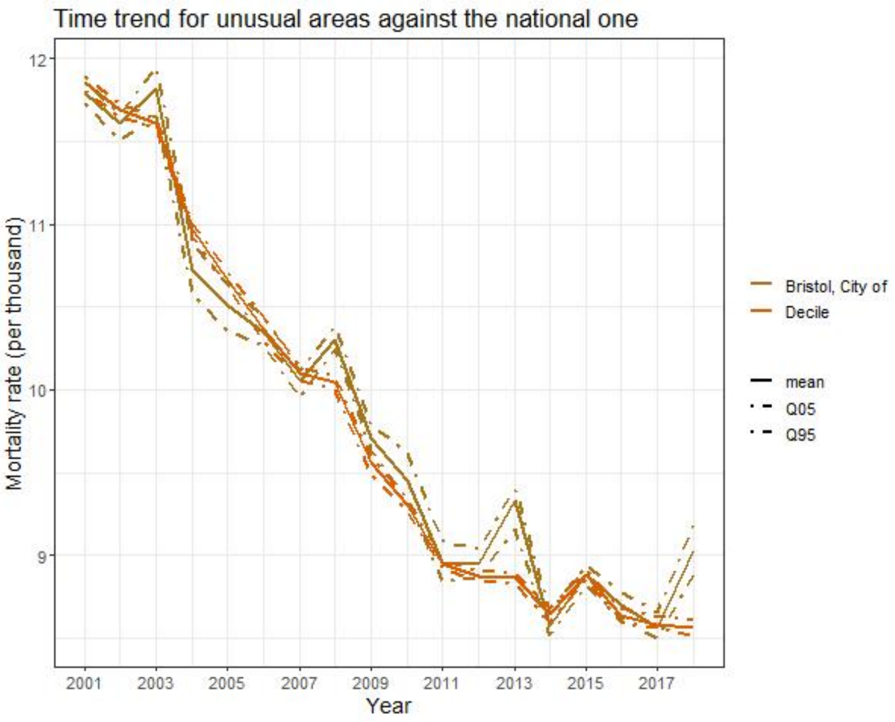

Figure S22: Cornwall including Isles of Scilly, males

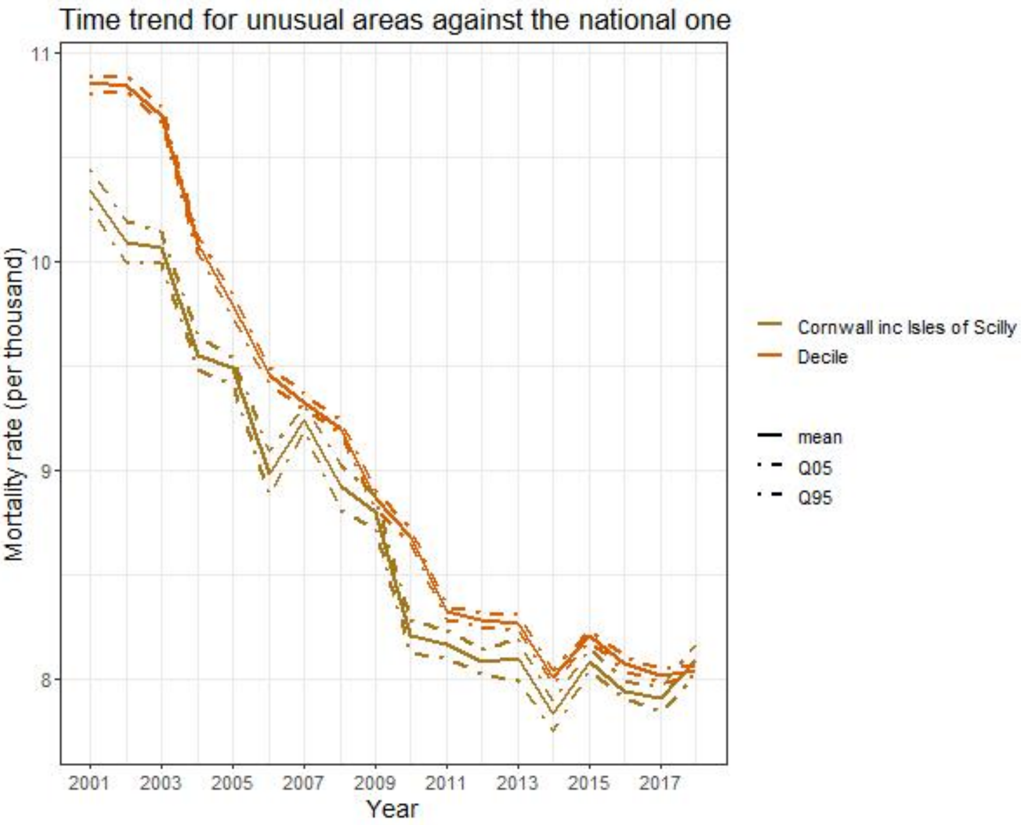

Figure S23: Breckland, males

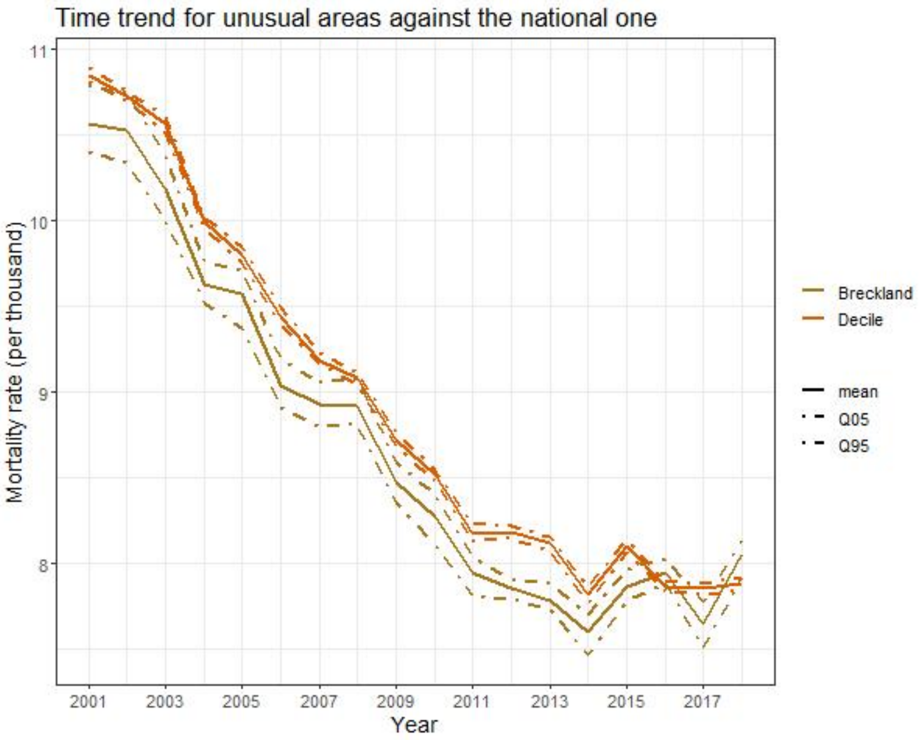

Figure S24: Ashford, males

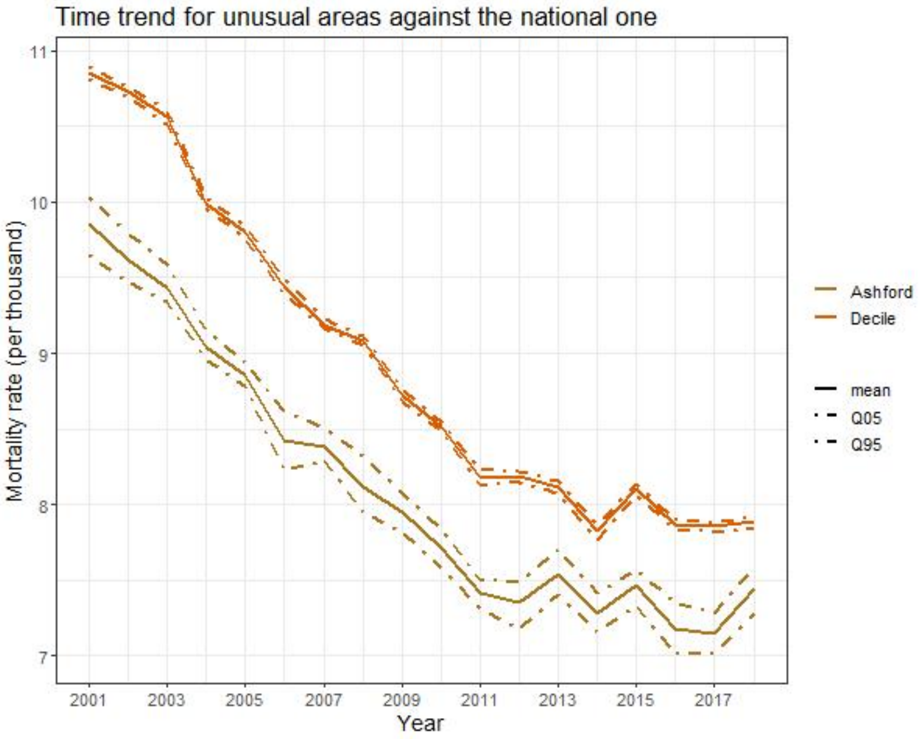

Figure S25: Hillingdon, males

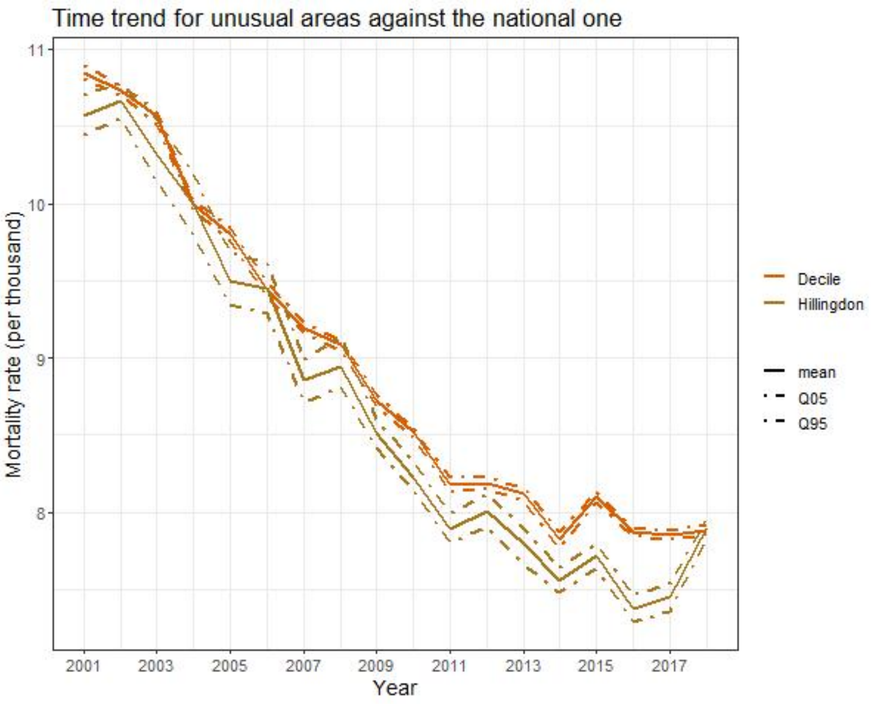

Figure S26: Cheshire West and Chester, males

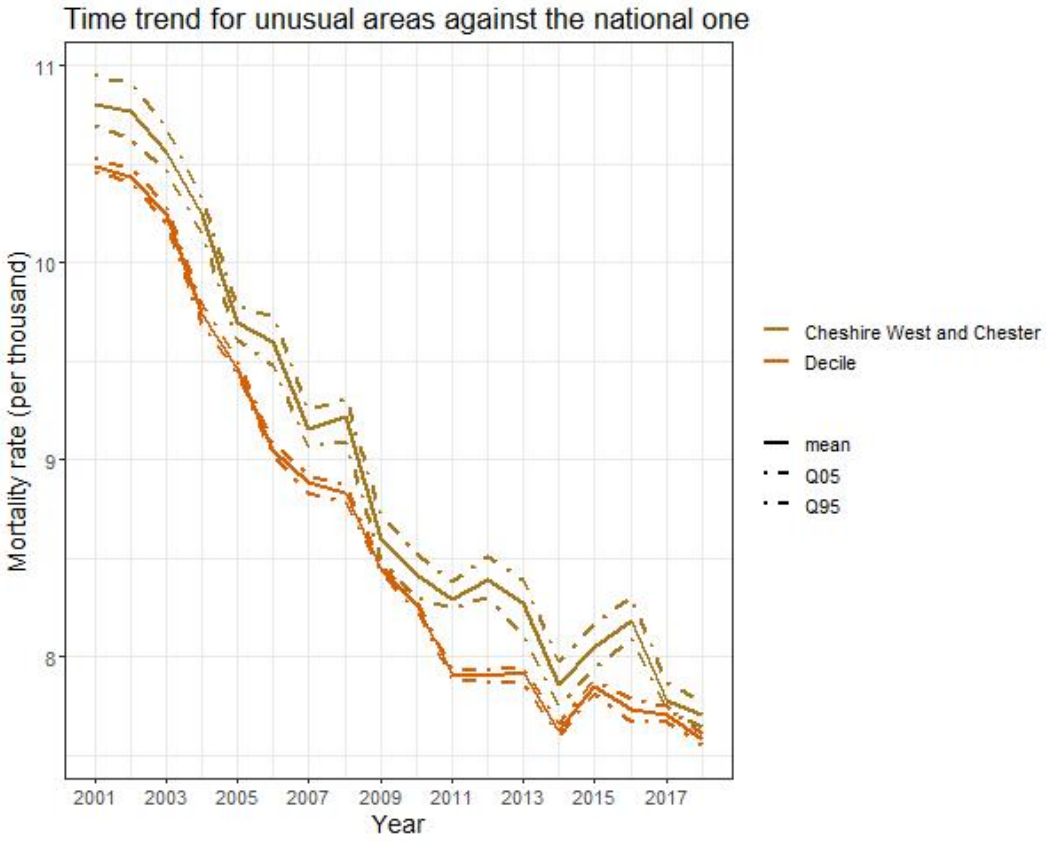

Figure S27: Dorset council, males

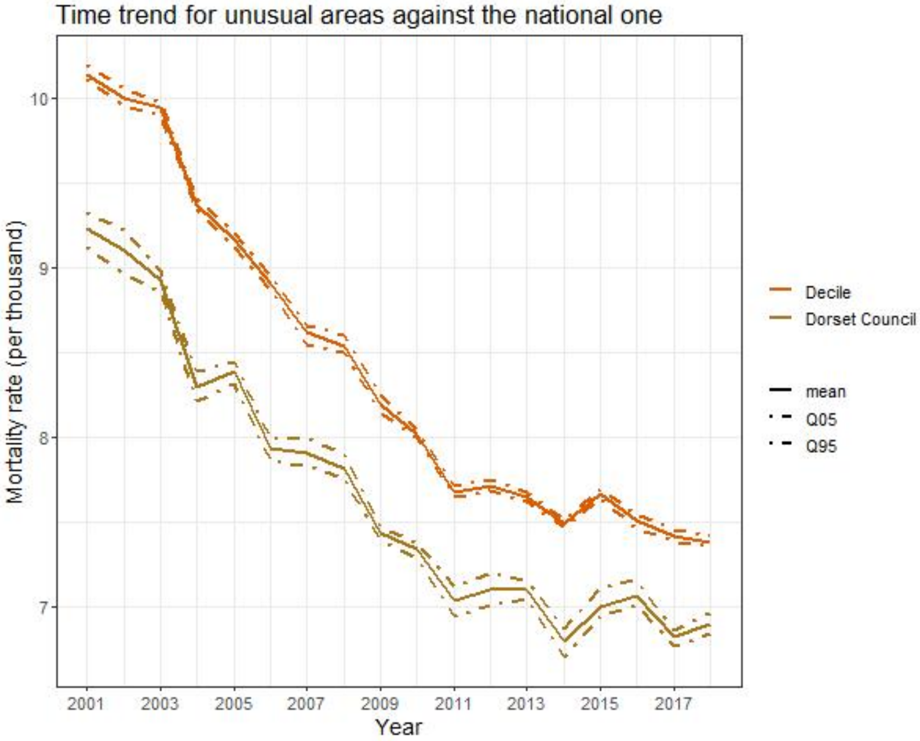

Figure S28: Teignbridge, males

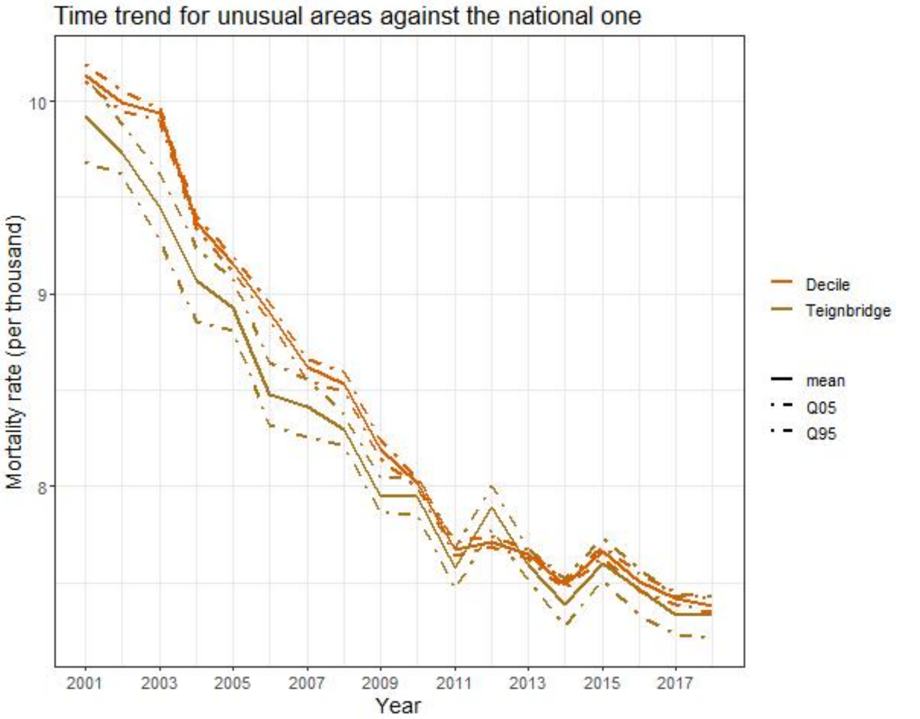

Figure S29: Stroud, males

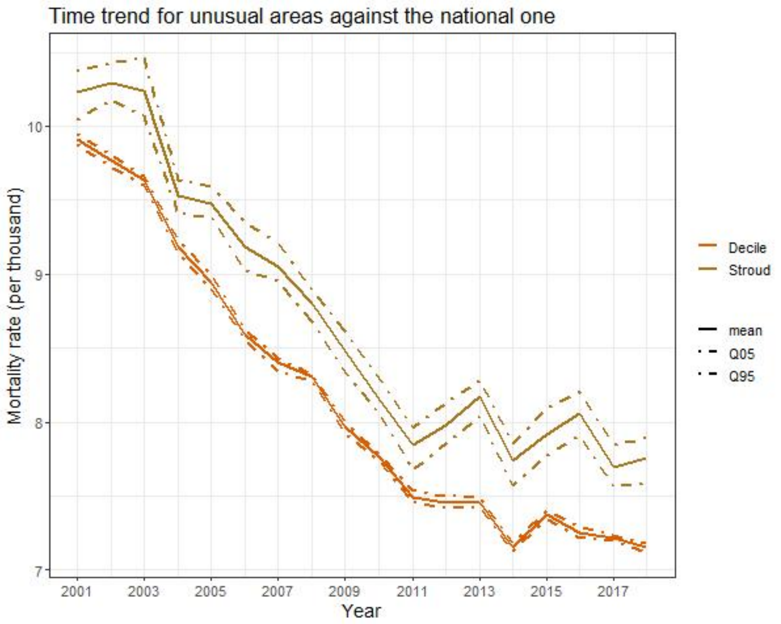

Figure S30: Bath and North East Somerset, males

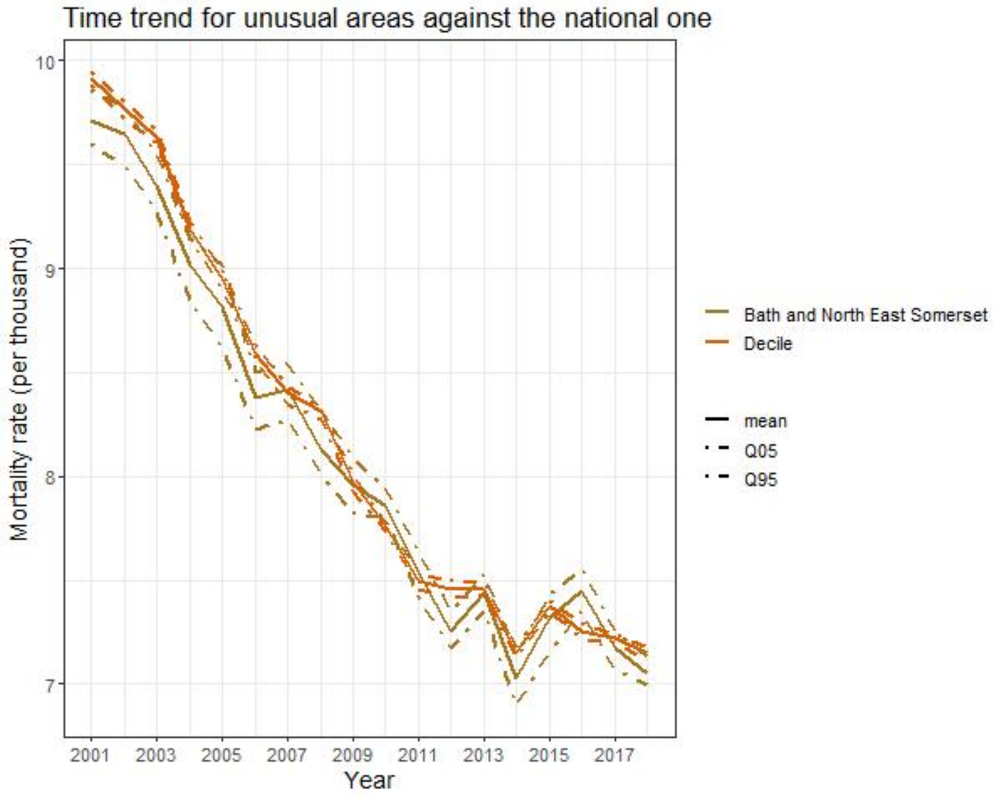

Supplement: Supplementary data [file bmjopen-2020-036855supp001.pdf]
