# Supplementary material for: Spatio-temporal model to estimate life expectancy and to detect unusual trends at the local authority level in England
Source: BMJ Open. 2020 Nov 12;10(11):e036855. doi: 10.1136/bmjopen-2020-036855 (PMC7662413; doi:10.1136/bmjopen-2020-036855)

BMJ Open is committed to open peer review. As part of this commitment we make the peer review history of every article we publish publicly available.

When an article is published we post the peer reviewers' comments and the authors' responses online. We also post the versions of the paper that were used during peer review. These are the versions that the peer review comments apply to.

The versions of the paper that follow are the versions that were submitted during the peer review process. They are not the versions of record or the final published versions. They should not be cited or distributed as the published version of this manuscript.

BMJ Open is an open access journal and the full, final, typeset and author-corrected version of record of the manuscript is available on our site with no access controls, subscription charges or pay-per-view fees (http://bmjopen.bmj.com).

If you have any questions on BMJ Open's open peer review process please email info.bmjopen@bmj.com

BMJ Open

Where is life expectancy stalling? Detecting local authorities in England across deprivation deciles

Journal:	*BMJ Open*
Manuscript ID	bmjopen-2020-036855
Article Type:	Original research
Date Submitted by the Author:	10-Jan-2020
Complete List of Authors:	Boulieri, Areti; Imperial College London, MRC-PHE Centre for Environment and Health, Department of Epidemiology and Biostatistics, School of Public Health, Epidemiology and Biostatistics<br>Blangiardo, Marta; Imperial College London, MRC-PHE Centre for Environment and Health, Department of Epidemiology and Biostatistics, School of Public Health
Keywords:	PUBLIC HEALTH, STATISTICS & RESEARCH METHODS, EPIDEMIOLOGY

SCHOLARONE™
Manuscripts

I, the Submitting Author has the right to grant and does grant on behalf of all authors of the Work (as defined in the below author licence), an exclusive licence and/or a non-exclusive licence for contributions from authors who are: i) UK Crown employees; ii) where BMJ has agreed a CC-BY licence shall apply, and/or iii) in accordance with the terms applicable for US Federal Government officers or employees acting as part of their official duties; on a worldwide, perpetual, irrevocable, royalty-free basis to BMJ Publishing Group Ltd ("BMJ") its licensees and where the relevant Journal is co-owned by BMJ to the co-owners of the Journal, to publish the Work in this journal and any other BMJ products and to exploit all rights, as set out in our licence.

The Submitting Author accepts and understands that any supply made under these terms is made by BMJ to the Submitting Author unless you are acting as an employee on behalf of your employer or a postgraduate student of an affiliated institution which is paying any applicable article publishing charge ("APC") for Open Access articles. Where the Submitting Author wishes to make the Work available on an Open Access basis (and intends to pay the relevant APC), the terms of reuse of such Open Access shall be governed by a Creative Commons licence – details of these licences and which Creative Commons licence will apply to this Work are set out in our licence referred to above.

Other than as permitted in any relevant BMJ Author's Self Archiving Policies, I confirm this Work has not been accepted for publication elsewhere, is not being considered for publication elsewhere and does not duplicate material already published. I confirm all authors consent to publication of this Work and authorise the granting of this licence.

For peer review only

Where is life expectancy stalling? Detecting local authorities in England across deprivation deciles

Areti Boulieri[a,*], Marta Blangiardo[a]

[a] Department of Epidemiology and Biostatistics, MRC PHE Centre for Environment and Health, Imperial College London, London, UK

[*] Corresponding author:
Areti Boulieri
Imperial College London, St Mary's Campus,
Norfolk Place, W2 1PG
a.boulieri@imperial.ac.uk

For peer review only

Keywords:
life expectancy, anomaly detection, area-level deprivation

Words: 3462

Abstract

Objectives

To estimate life expectancy at the local authority level and detect those areas that have a substantially low life expectancy after accounting for deprivation.

Design

We used registration data from the Office for National Statistics (ONS) on mortality and population in England, by local authority, age group, and deprivation decile, for both males and females. We used a statistical model within the Bayesian framework to produce robust mortality rates, which were then transformed to life expectancy estimates. A rule based on exceedance probabilities was finally used to detect local authorities characterised by a low life expectancy among areas with a similar deprivation level.

Results

We confirmed previous findings showing differences in the life expectancy gap between the most and least deprived areas from 2012 to 2018. We found variations in life expectancy trends across local authorities, and we detected a number of those with a low life expectancy when compared to others of a similar deprivation level.

Conclusions

There are factors other than deprivation that are responsible for low life expectancy in certain local authorities. Further investigation on the detected areas can help with understanding better the stalling of life expectancy which was observed from 2012 onwards and help with efficient public health policies.

Article summary

Article focus

- Estimate life expectancy in England from 2001 to 2018 for males and females by deprivation decile
- Assess whether the life expectancy gap over the 18 year period varies across deprivation deciles
- Detect local authorities with a low life expectancy compared to others of a similar deprivation level

Key messages

- The overall level of life expectancy is different across deciles, with higher deprivation corresponding to lower life expectancy
- There is a difference of more than 100 days in life expectancy gains for the least deprived decile compared to the most deprived one for both sexes
- There are variations in life expectancy trends across local authorities, and a number of those were detected with a low life expectancy for their deprivation decile; in particular, 10 unique areas were detected for females and 19 for males

For peer review only

Strengths and limitations of this study

- This is the first study of its scale, including more than 8 million records.
- Additional strengths include the methodology which produces robust estimates, as well as the ability to detect specific local authorities in England, and therefore help better understand the stalling of life expectancy and implement efficient public health measures.
- It was not possible to account for population changes due to migration in the model. In addition, the socio-economic indicator that was used is based on data from a specific year, however changes in the deprivation level of local authorities over the years were minimal.

Introduction

The study of life expectancy is of primary interest in public health practice, where it is used to measure the overall health of a population, and also to plan for health and social services. Recently, it has attracted substantial attention, due to the stalling effect that has been observed in several countries, including the USA and the UK.[1,2] That is, while life expectancy has been improving steadily over the last decades, mainly due to changes in behavioural risk factors and healthcare, this phenomenon has started to slow down from 2012 onwards. Possible explanations of this phenomenon include strikes in influenza among older people, potentially due to ineffective vaccines,[3] and excess mortality of dementia and Alzheimer's disease, now that the number of treatable diseases is growing.[4] However, recent studies in the UK found that the excess number of deaths in 2015 were largely driven by the older populations, suggesting potential associations with socioeconomic factors, poor health and social care, which can be linked to reductions in spending across the healthcare system.[5,6,7]

Socio-economic deprivation has been found to be the major determinant of life expectancy by numerous studies over the years, regardless of the measure of deprivation that was used. Geographical patterns of life expectancy in England and Wales were found to be mainly attributable to variations in deprivation status in 1998,[8] while a more recent study found that the elimination of socio-economic differences between areas would increase survival among older adults across a number of European countries, with a higher magnitude in England.[9] The impact of deprivation on overall levels of life expectancy is therefore established. However, the corresponding impact on life expectancy improvements over time is less clear; an early paper found that in most deprived areas improvements in life expectancy were negligible,[10] while a more recent paper showed that the life expectancy gap between the most affluent and most deprived areas increased from 2001 to 2016 for both sexes, with a higher magnitude for females.[11] This finding was not confirmed by later studies where largest improvements in life expectancy were documented in some of the most deprived areas.[12] Recent life expectancy trends have been studied in comparison to socio-economic deprivation mostly at the decile level, i.e. by aggregating areas in England into deciles according to their measure of area deprivation.[11,13,14,15] Only limited research has investigated relevant associations at the smaller area level, including a study in Scotland[16] and one in Sweden.[17]

The majority of previous studies have used a low geographical resolution. It is nevertheless crucial to focus on local authorities and detect those that are most in need, as this would help better understand the life expectancy gap and consequently support governments with evidence for prioritisation and more efficient public health policies. This cannot rely solely on data description, as splitting the number of deaths by a combination of small area, age, gender and year leads to low number of cases and in turn to unstable mortality rates and consequently life expectancy estimates. The need for a modelling approach has been highlighted before to overcome this issue.[18] Additionally, we argue that it is important to establish the role of deprivation in detecting areas

For peer review only

performing poorly in terms of life expectancy, as well as at the same time to detect those areas that differ from others with a similar level of socio-economic deprivation and show unexplained lower life expectancy. These local authorities should be investigated further to understand which factors are driving the stalling of life expectancy, in order to direct policies and intervention measures.

To the best of our knowledge, there has been no study on life expectancy in England focusing on the local authority level, while accounting for deprivation. In this paper, we analyse all-cause mortality counts in England at the local authority level from 2001 to 2018 in order to assess the impact of deprivation on life expectancy improvements over time. Moreover, focusing on 2012-2018 when the stalling effect was observed, we detect areas which have shown lower life expectancy than other areas characterised by a similar level of social deprivation.

Methods

Data

We used mortality counts from 2001 to 2018 at the local authority level in England. In total 315 local authorities were considered. Information on age and sex was available for each record. We considered 20 age groups, each including 5 years except for the first (< 1 year old) and the last group (90 and older) and we analysed males and females separately as these have previously shown to have very different mortality levels and trends.[19] Population data by age and sex, as well as a measure of deprivation for each local authority were also used for the analysis. All data were provided by Public Health England (PHE), originally held by Office for National Statistics (ONS).

Deprivation data were available at the decile level. In this study we used the English index of multiple deprivation (IMD), based on seven different domains of deprivation: i) income deprivation, ii) employment deprivation, iii) education, skills and training deprivation, iv) health deprivation and disability, v) crime, vi) barriers to housing and services, and vii) living environment deprivation. Each local authority was allocated to a deprivation decile (D1, …, D10) with D1 being the most deprived and D10 the least deprived decile based on the IMD 2019.[20]

Patient and public involvement

As this is secondary research based on aggregated data that have already been collected, no patients or the public were involved in designing the study, nor were they involved in the analysis or implementation of the study. Dissemination to study participants or patient organisations is not applicable.

Statistical analysis

We developed a statistical model to analyse mortality counts by age group, local authority, calendar year, and deprivation decile. Mortality counts were modelled assuming a Poisson distribution, and the population at risk was included as the denominator, so that the model yields estimates of mortality rates.

The model was designed to incorporate assumptions regarding the structure of the mortality counts. It is expected that mortality rates vary with age, time, and across space and their trends tend to have a smooth pattern. We therefore allowed different trends by age, and across time and space, and assumed that units that are close to each other (in terms of age groups, local authorities, or years) are likely to have more similar levels of mortality than those farther away. Pairwise interactions between age and space, age and time, and space and time were also included in the model to increase the level of flexibility of the model.

Deciles of deprivation were included in the model, as well as an interaction term between decile and calendar year, assuming a similar behaviour in time for neighbouring deciles.

For peer review only

A rule to detect local authorities characterised by lower life expectancy was specified based on the space-time interaction term of the model, that, if above zero, would suggest a local increase of mortality rates. We estimated the probability that the space-time interaction term is above zero for all space-time combinations and we selected the local authorities with such probability above 0.95. We restricted the detection on 2012-2018 to focus on the period characterised by the stalling of life expectancy.

The model was implemented within the Bayesian framework which naturally caters for dependence across age, time and space. Results are presented as mean estimates and 95% credible intervals (CI). Mortality rates were transformed to life expectancy estimates for each local authority using life tables methods.[21]

Results

Life expectancy at birth in England was estimated to be 79.6 years for men and 83.2 years for women in 2018. Over the period 2001 to 2018, life expectancy followed an overall increasing trend, with a flatter pattern from 2012 onwards for both genders, with the gap between women and men decreasing over time. Figure 1 shows the time trends of life expectancy by deprivation decile group for men and women respectively. It is observed that the overall level of life expectancy is different across deciles, with higher deprivation corresponding to lower life expectancy. Additionally, it can be noted that life expectancy follows an overall increasing trend for all deciles, however more deprived groups are characterised by flatter patterns compared to less deprived groups.

Focusing on 2012-2018, Figure 2 shows the estimates of life expectancy and their corresponding 95% CI's. Looking at the life expectancy gap between the beginning and the end of the time period across deprivation deciles, there is a pronounced difference of more than 100 days in life expectancy gains for the least deprived decile compared to the most deprived one for both sexes, while it is worth noting the large uncertainty across all the deciles (95% credibility intervals depicted by the error bars). When comparing males and females, we observe bigger gains for males across most deprivation deciles, however there is an overlap in the uncertainties of those two; these findings are in line with reports from ONS and PHE.[14,19]

We found variations in life expectancy trends across local authorities, and a number of those were detected as atypical (i.e. showing lower life expectancy than in the corresponding deprivation decile) from 2012 to 2018. The maps in Figures 3 and 4 show the detected local authorities for males and females respectively with the corresponding number of times that these were picked up out of the 7 year period. In particular, 10 unique areas were detected for females and 19 areas for males (Tables 1 and 2). Looking at the maps, we do not observe any specific spatial pattern in the detected local authorities, as these are randomly scattered across England. From the tables it can be noted that roughly half of the detected local authorities belong to the two most deprived deciles (D1 and D2) for both sexes. Additionally, most local authorities were detected in 1 year only during the 7 year period, with Leeds standing out as it was detected in 2 years for females and 4 years for males.

Discussion

In this analysis we have investigated the life expectancy gap between the most and least deprived local authorities in England in order to inform policy implementation and prioritisation. Most importantly, we have proposed a statistical tool to look at the life expectancy trends across local authorities and to detect those characterised by lower levels which should be further investigated in order to provide targeted policy measures.

Principal findings

For peer review only - http://bmjopen.bmj.com/site/about/guidelines.xhtml

Our analyses suggest differences in the life expectancy gap between the most and least deprived areas from 2012 to 2018, for both males and females, confirming previous findings.[11,19] Particularly, the difference in life expectancy improvements in the wealthiest areas (decile 10) compared to the poorest areas (decile 1) in England was more than 100 days for males, and roughly 80 days for females. The corresponding differences among deprivation deciles 2 to 9 were less clear considering the uncertainty around the estimates.

We found that the life expectancy trends did not follow the same pattern across all local authorities for both males and females between 2012 and 2018. More specifically, we detected the local authorities which depicted a lower life expectancy compared to what would have been expected given their deprivation level. This suggests factors other than deprivation that are potentially responsible for the poor performance of the specific local authorities, and would call for further investigation by public health bodies and therefore prioritisation for interventions.

We note here that our study is descriptive as it does not formally assess potential causes in the detected areas. Nevertheless, we expect that it will inform the evidence around life expectancy gaps and the role of deprivation; this should be used as the basis for future studies targeting the detected local authorities to investigate potential responsible factors, to be conducted in collaboration with epidemiologists and public health researchers.

The follow up study on the detected areas should consider disentangling the different domains of socio-economic deprivation, such as barriers to housing and services, or health deprivation and disability, to see if any of these domains is dragging the life expectancy of an otherwise non-deprived local authority down. Austerity has been linked to the stalling of life expectancy;[22] this is another aspect which should be further investigated, particularly looking at how different cities have implemented specific local policies related to austerity which might have contributed to the different life expectancy trends. Furthermore, it would worth investigating the different causes of death separately to help with hypothesis making; for example, if the low life expectancy of an area is attributed to teenage suicides, this might indicate unprotected environments in specific schools of that area. Finally, any events or changes in a neighbourhood should be explored to assess any potential impact these might have on their life expectancy; as an example, if a hospital that provides long-term care is built in an area, that area will experience unusually low life expectancy.

Comparison to previous studies

Our life expectancy estimates are in line with the ONS estimates[19] for both males and females. In addition, our findings that deprivation has an impact not only on the overall life expectancy estimates but also on life expectancy improvements has been previously shown.[14] This is in line with an earlier study investigating the impact of a health inequalities intervention, implemented by the UK government between 1997 and 2010; the authors showed that the life expectancy gap between the population that lives in the 20% most deprived local authorities and the rest of the population declined during the intervention, while this trend was reversed after the intervention ended in 2010.[23]

Differently from Bennett and others[11] who run the analyses at the deprivation decile, the novelty of our study lies on the analysis of life expectancy at the local authority level, while accounting for deprivation. This allows us to propose a mechanism to detect local authorities in England with lower life expectancy for their deprivation level and therefore provide a first step in the investigation and understanding of factors other than deprivation that might be responsible in order to develop targeted interventions.

Strengths and limitations

Our analysis of mortality registration data in England from 2001 to 2018 has a number of advantages. Firstly, to our knowledge, it is the first study of its scale, investigating life expectancy

For peer review only

at the local authority level in the whole of England over an 18-year period, including more than 8 million records. Second, the methodology that we use, appropriate for the small numbers that arise when mortality counts are considered by local authority, year, and age group, produces robust estimates, unlike the standard statistical techniques frequently used for this type of analysis.[24] Third, we are able to detect specific local authorities in England which are most left behind in terms of life expectancy improvements, addressing an important gap in the literature. We finally propose a statistical tool that can be used on a routine basis for detection of life expectancy anomalies in England while accounting for deprivation. While a segment tool is available by PHE to provide information on the life expectancy gap at the local authority level,[25] this is based on crude mortality rates and therefore less stable compared to the smoothed ones produced through statistical modelling, it provides mean estimates over 2015-2017 and not annual estimates, and most importantly it is not able to automatically flag areas which would require further investigation, but one should rather examine these individually.

Limitations of our study include population changes because of migration, both within the country and overseas, which are not accounted for in the model. In addition, the allocation of local authorities into discrete groups of deprivation, will inevitably yield some variation within local authorities, since for example not all people can be of the same socio-economic status, which is a common issue in ecological studies. Lastly, the socio-economic indicator that we use is based on data from a specific year, and does not account for changes in the local authorities between deprivation deciles. We need to note however that although there have been changes in the ranking order of a few local authorities from 2015, when the previous deprivation data were released, to 2019, which we are currently using, these were not large enough to impact the corresponding deprivation decile group. Besides, comparison of data between the years shows a very high association.[20]

Conclusions

Much research has recently focused on life expectancy to understand the underlying causes of the time trends observed in England. Although these shed light into potential factors that are driving these, we believe it is of high importance to use a detection mechanism as part of a routine monitoring surveillance in order to focus on specific areas that are most in need.

Detection of specific local authorities in England, while accounting for socio-economic deprivation, would highlight those areas that perform worse compared to others of a similar deprivation level. Our study contributes to the state of the art by proposing a statistical tool to perform this type of anomaly detection.

Footnotes

Contributors

All authors conceived and designed the study. AB ran the statistical analysis. All authors contributed to interpreting the results, and revising the manuscript. The corresponding author attests that all listed authors meet authorship criteria and that no others meeting the criteria have been omitted.

Acknowledgements

The authors would like to thank Garyfallos Konstantinoudis, Monica Pirani and Chiara Forlani from Imperial College London and Allan Baker, Paul Fryers from PHE for their constructive comments which helped improve the paper. Areti Boulieri acknowledges support from an MRC Early Career Research Fellowship (MR/M501669/1). Marta Blangiardo acknowledges support by Public Health England award titled “Assessment of Trends in Life Expectancy in England and its Upper-tier Local authorities”.

For peer review only - http://bmjopen.bmj.com/site/about/guidelines.xhtml

Competing interests

None.

Ethical approval

Not required.

Data sharing

The data used for the study were provided by Public Health England (PHE) and cannot be shared online due to confidentiality.

Transparency declaration

The lead author (AB) affirms that the manuscript is an honest, accurate, and transparent account of the study being reported; that no important aspects of the study have been omitted; and that any discrepancies from the study as planned have been explained.

Local authority	Decile	Year
Kingston upon Hull, City of	D1	2017
Stoke-on-Trent	D1	2018
Tameside	D1	2018
Leeds	D2	2015, 2018
Newcastle upon Tyne	D2	2015
Plymouth	D2	2017
Derby	D3	2018
Cornwall inc Isles of Scilly	D4	2016
Bournemouth, Christchurch and Poole	D6	2015, 2017
Sutton	D8	2014

Table 1: Local authorities with a low life expectancy for their deprivation level for females

For peer review only

Local authority	Decile	Year
Wolverhampton	D1	2018
Kingston upon Hull, City of	D1	2017
Birmingham	D1	2014
Blackpool	D1	2015
Middlesbrough	D1	2017
Tendring	D2	2017
Leeds	D2	2012, 2015, 2017, 2018
Newcastle upon Tyne	D2	2014
Southampton	D2	2013
Bristol, City of	D3	2013, 2018
Cornwall inc Isles of Scilly	D4	2018
Breckland	D5	2018
Ashford	D5	2018
Hillingdon	D5	2018
Cheshire West and Chester	D6	2016
Dorset Council	D7	2016
Teignbridge	D7	2012
Stroud	D9	2016
Bath and North East Somerset	D9	2016

Table 2: Local authorities with a low life expectancy for their deprivation level for males

References

[1] Olshansky, S Jay, Douglas J Passaro, Ronald C Hershow, Jennifer Layden, Bruce A Carnes, Jacob Brody, Leonard Hayflick, Robert N Butler, David B Allison, and David S Ludwig. 2005. “A

For peer review only

Potential Decline in Life Expectancy in the United States in the 21st Century." *New England Journal of Medicine* 352 (11). Mass Medical Soc: 1138–45.

[2] Hiam, Lucinda, Dominic Harrison, Martin McKee, and Danny Dorling. 2018. "Why Is Life Expectancy in England and Wales 'Stalling'?" *J Epidemiol Community Health* 72 (5). BMJ Publishing Group Ltd: 404–8.

[3] Pebody, RG, HK Green, F Warburton, M Sinnathamby, J Ellis, K Mølbak, J Nielsen, S de Lusignan, and N Andrews. 2018. "Significant Spike in Excess Mortality in England in Winter 2014/15–Influenza the Likely Culprit." *Epidemiology & Infection*. Cambridge University Press, 1–8.

[4] Liddell, Christine, Chris Morris, Barbara Gray, Anna Czerwinska, and Ben Thomas. 2016. "Excess Winter Mortality Associated with Alzheimer's Disease and Related Dementias in the Uk: A Case for Energy Justice." *Energy Research & Social Science* 11. Elsevier: 256–62.

[5] Hiam, Lucinda, Danny Dorling, Dominic Harrison, and Martin McKee. 2017a. "What Caused the Spike in Mortality in England and Wales in January 2015?" *Journal of the Royal Society of Medicine* 110 (4). SAGE Publications Sage UK: London, England: 131–37.

[6] Hiam, Lucinda, Danny Dorling, Dominic Harrison, and Martin McKee. 2017b. "Why Has Mortality in England and Wales Been Increasing? An Iterative Demographic Analysis." *Journal of the Royal Society of Medicine* 110 (4). SAGE Publications Sage UK: London, England: 153–62.

[7] Watkins, Johnathan, Wahyu Wulaningsih, Charlie Da Zhou, Dominic C Marshall, Guia DC Sylianteng, Phyllis G Dela Rosa, Viveka A Miguel, Rosalind Raine, Lawrence P King, and Mahiben Maruthappu. 2017. "Effects of Health and Social Care Spending Constraints on Mortality in England: A Time Trend Analysis." *BMJ Open* 7 (11). British Medical Journal Publishing Group: e017722.

[8] Woods, Laura M, Bernard Rachet, Michael Riga, Noell Stone, Anjali Shah, and Michel P Coleman. 2005. "Geographical Variation in Life Expectancy at Birth in England and Wales Is Largely Explained by Deprivation." *Journal of Epidemiology & Community Health* 59 (2). BMJ Publishing Group Ltd: 115–20.

[9] Ribeiro, Ana Isabel, Elias Teixeira Krainski, Marilia Sá Carvalho, Guy Launoy, Carole Pornet, and Maria de Fátima de Pina. 2018. "Does Community Deprivation Determine Longevity After the Age of 75? A Cross-National Analysis." *International Journal of Public Health* 63 (4). Springer: 469–79.

[10] Raleigh, Veena Soni, and Victor A Kiri. 1997. "Life Expectancy in England: Variations and Trends by Gender, Health Authority, and Level of Deprivation." *Journal of Epidemiology & Community Health* 51 (6). BMJ Publishing Group Ltd: 649–58.

[11] Bennett, James E, Jonathan Pearson-Stuttard, Vasilis Kontis, Simon Capewell, Ingrid Wolfe, and Majid Ezzati. 2018. "Contributions of Diseases and Injuries to Widening Life Expectancy Inequalities in England from 2001 to 2016: A Population-Based Analysis of Vital Registration Data." *The Lancet Public Health* 3 (12). Elsevier: e586–e597.

[12] Public Health England. 2019a. "Recent Trends in Mortality in England: Review and Data Packs. A Report on Recent Trends in Life Expectancy and Mortality in England." https://www.gov.uk/government/publications/recent-trends-in-mortality-in-england-review-and-data-packs.

[13] Mayhew, Les, Gillian Harper, and Andrés M Villegas. 2019. "An Investigation into the Impact of Deprivation on Demographic Inequalities in Adults." *Annals of Actuarial Science*. Cambridge

For peer review only

University Press (CUP).

[14] Public Health England. 2018. "Health Profile for England: 2018." https://www.gov.uk/government/publications/health-profile-for-england-2018.

[15] Iacobucci, Gareth. 2019. "Life Expectancy Gap Between Rich and Poor in England Widens." British Medical Journal Publishing Group.

[16] Seaman, Rosie, Tim Riffe, Alastair H Leyland, Frank Popham, and Alyson van Raalte. 2019. "The Increasing Lifespan Variation Gradient by Area-Level Deprivation: A Decomposition Analysis of Scotland 1981–2011." *Social Science & Medicine* 230. Elsevier: 147–57.

[17] Åström, Daniel Oudin, Paul W Franks, and Kristina Sundquist. 2018. "Neighborhoods and Mortality in Sweden: Is Deprivation Best Assessed Nationally or Regionally?" *Demographic Research* 38. JSTOR: 429–50.

[18] Newton, John N. 2018. "Dissecting the Life Expectancy Gap in England Provides Clues on How to Reduce It." *The Lancet Public Health* 3 (12). Elsevier: e560–e561.

[19] Office for National Statistics. 2019a. "National Life Tables, Uk: 2016 to 2018." https://www.ons.gov.uk/releases/nationallifetablesuk2016to2018.

[20] Ministry of Housing, Communities, & Local Government. 2019. "National Statistics: The English Indices of Deprivation 2019 (Iod2019)."

[21] Office for National Statistics. 2019b. "Data: National Life Tables, England." https://www.ons.gov.uk/peoplepopulationandcommunity/birthsdeathsandmarriages/lifeexpectancies/datasets/nationallifetablesenglandreferencetables.

[22] Christie, Bryan. 2019. "Study Cites Austerity as Factor in Stalling of Life Expectancy in Rich Countries." *BMJ: British Medical Journal (Online)* 364. BMJ Publishing Group LTD.

[23] Barr, Ben, James Higgerson, and Margaret Whitehead. 2017. "Investigating the Impact of the English Health Inequalities Strategy: Time Trend Analysis." *bmj* 358. British Medical Journal Publishing Group: j3310.

[24] White, Chris. 2015. "Trend in Life Expectancy at Birth and at Age 65 by Socio-Economic Position Based on the National Statistics Socio-Economic Classification, England and Wales: 1982-1986 to 2007-2011." *Statistical Bulletin*.

[25] Public Health England. 2019b. "Segment Tool: 2019 Data Update."https://www.gov.uk/government/statistics/segment-tool-2019-data-update.

For peer review only

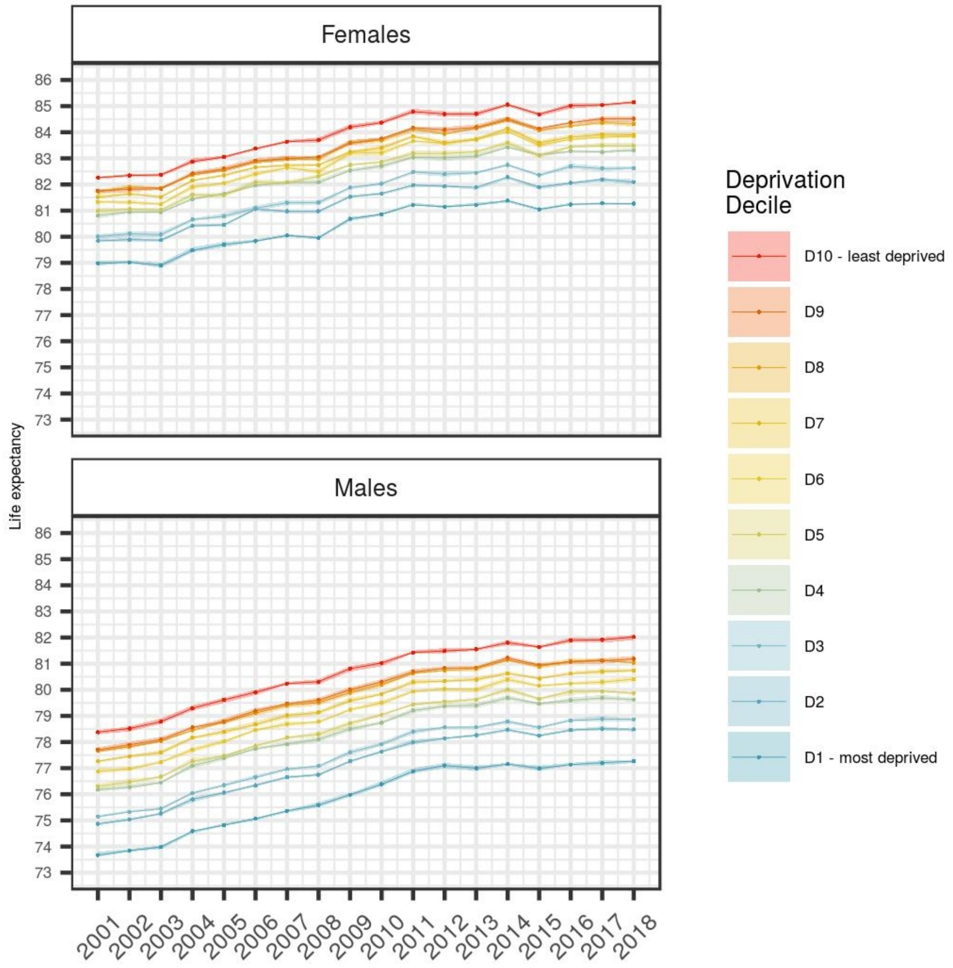

Figure 1: Life expectancy estimates from 2001 to 2018 across deprivation deciles; the shaded areas correspond to 95% CI's

89x89mm (300 x 300 DPI)

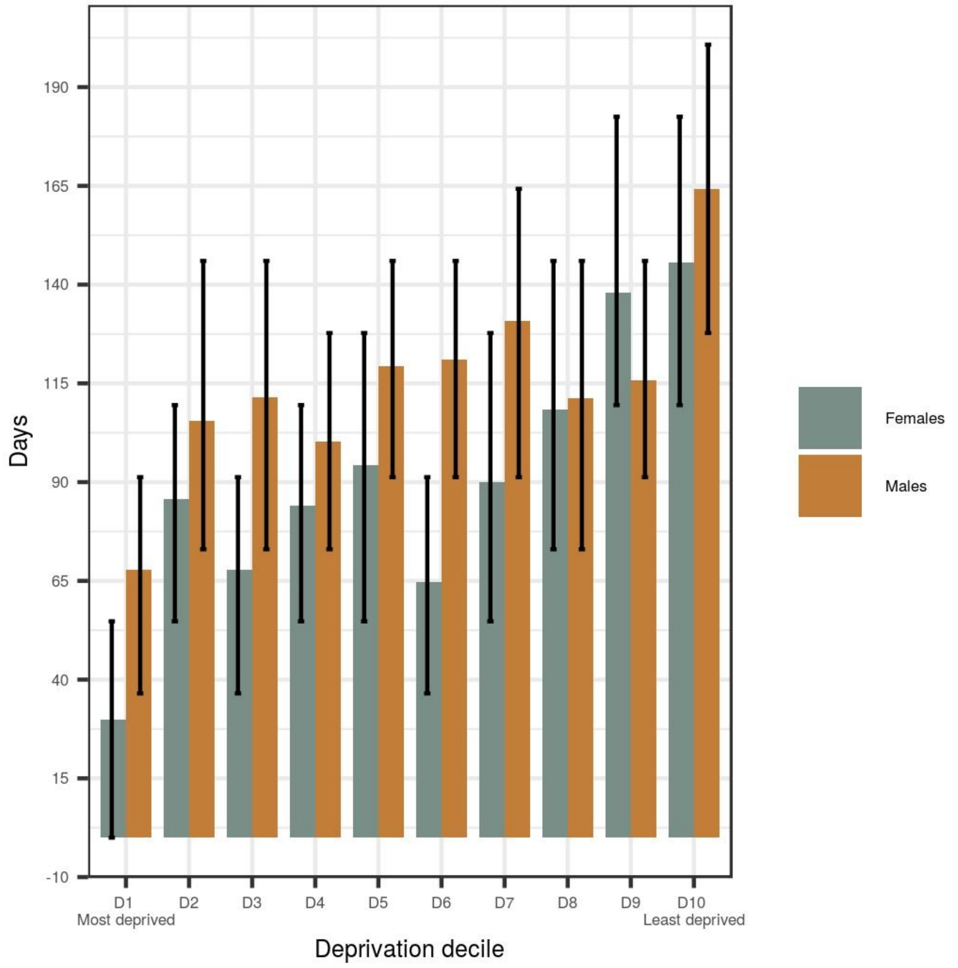

Figure 2: Life expectancy gap between 2012-2013 and 2017-2018; the error bars correspond to 95% CI's

89x89mm (300 x 300 DPI)

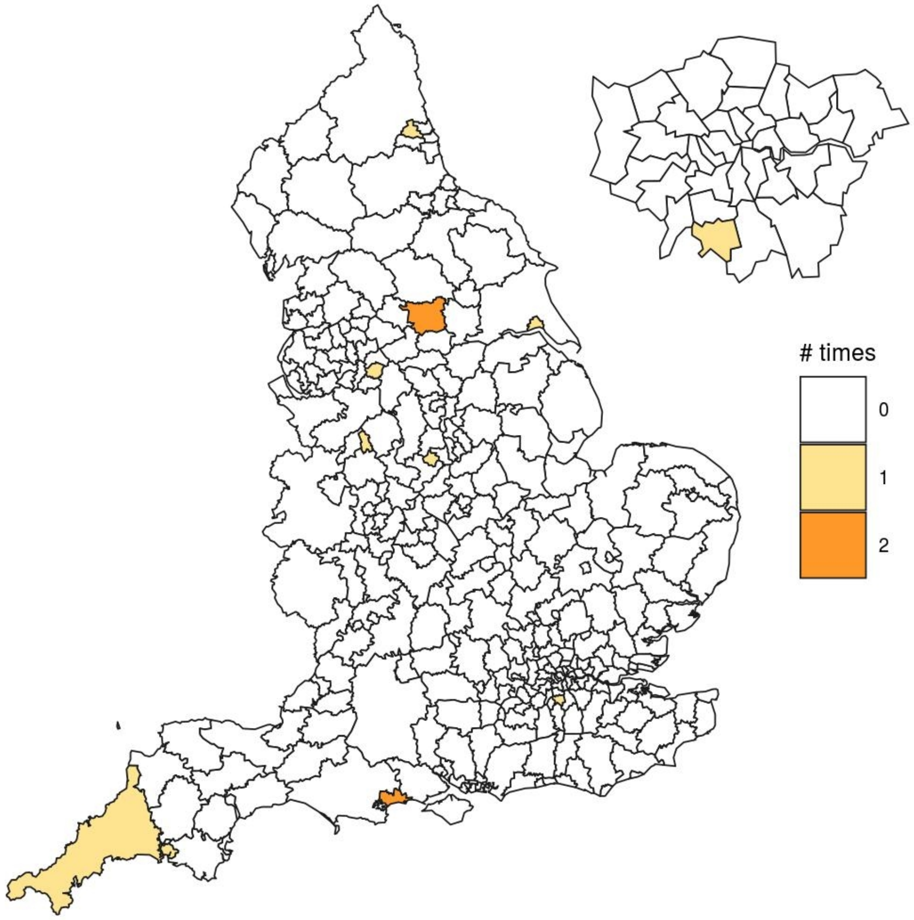

Figure 3: Local authorities detected with a lower life expectancy than others with a similar deprivation level for females

89x89mm (300 x 300 DPI)

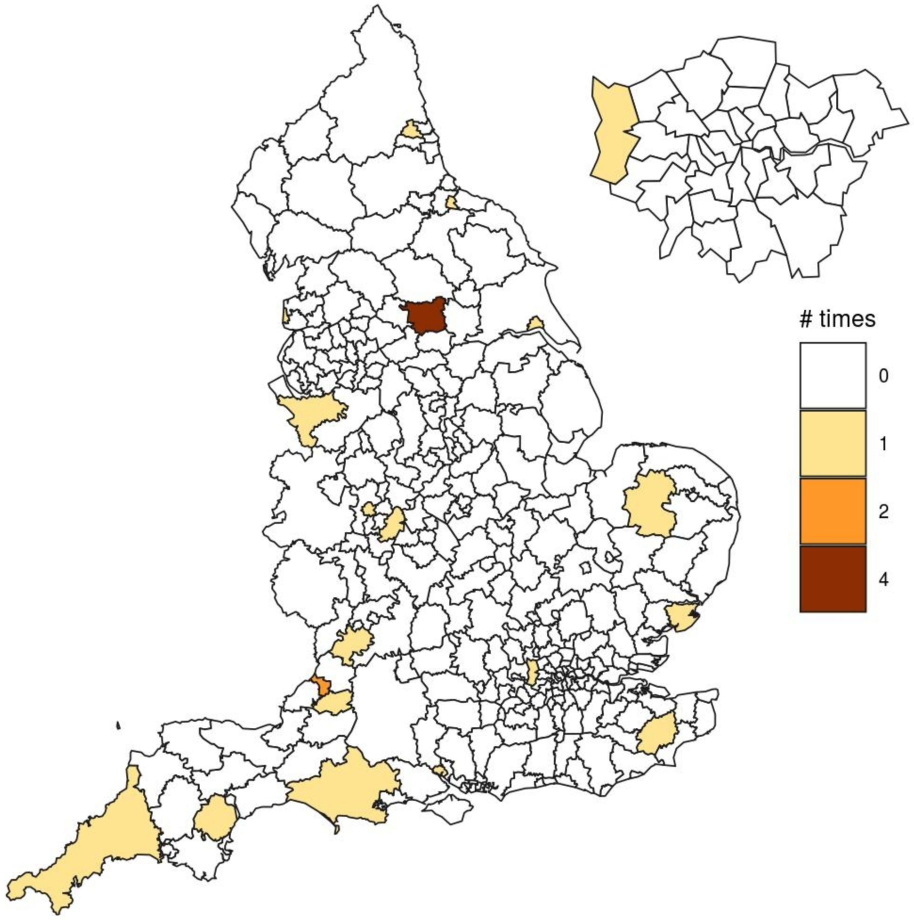

Figure 4: Local authorities detected with a lower life expectancy than others with a similar deprivation level for males

89x89mm (300 x 300 DPI)

BMJ Open

A spatio-temporal model to estimate life expectancy and to detect unusual trends at the local authority level in England

Journal:	*BMJ Open*
Manuscript ID	bmjopen-2020-036855.R1
Article Type:	Original research
Date Submitted by the Author:	24-Jul-2020
Complete List of Authors:	Boulieri, Areti; Imperial College London, MRC-PHE Centre for Environment and Health, Department of Epidemiology and Biostatistics, School of Public Health, Epidemiology and Biostatistics<br>Blangiardo, Marta; Imperial College London, MRC-PHE Centre for Environment and Health, Department of Epidemiology and Biostatistics, School of Public Health
**Primary Subject Heading**:	Epidemiology
Secondary Subject Heading:	Public health
Keywords:	PUBLIC HEALTH, STATISTICS & RESEARCH METHODS, EPIDEMIOLOGY

SCHOLARONE™
Manuscripts

I, the Submitting Author has the right to grant and does grant on behalf of all authors of the Work (as defined in the below author licence), an exclusive licence and/or a non-exclusive licence for contributions from authors who are: i) UK Crown employees; ii) where BMJ has agreed a CC-BY licence shall apply, and/or iii) in accordance with the terms applicable for US Federal Government officers or employees acting as part of their official duties; on a worldwide, perpetual, irrevocable, royalty-free basis to BMJ Publishing Group Ltd ("BMJ") its licensees and where the relevant Journal is co-owned by BMJ to the co-owners of the Journal, to publish the Work in this journal and any other BMJ products and to exploit all rights, as set out in our licence.

The Submitting Author accepts and understands that any supply made under these terms is made by BMJ to the Submitting Author unless you are acting as an employee on behalf of your employer or a postgraduate student of an affiliated institution which is paying any applicable article publishing charge ("APC") for Open Access articles. Where the Submitting Author wishes to make the Work available on an Open Access basis (and intends to pay the relevant APC), the terms of reuse of such Open Access shall be governed by a Creative Commons licence – details of these licences and which Creative Commons licence will apply to this Work are set out in our licence referred to above.

Other than as permitted in any relevant BMJ Author's Self Archiving Policies, I confirm this Work has not been accepted for publication elsewhere, is not being considered for publication elsewhere and does not duplicate material already published. I confirm all authors consent to publication of this Work and authorise the granting of this licence.

For peer review only

A spatio-temporal model to estimate life expectancy and to detect unusual trends at the local authority level in England

Areti Boulieri[a,], Marta Blangiardo[a*]

[a] Department of Epidemiology and Biostatistics, MRC Centre for Environment and Health, Imperial College London, London, UK

[*] Corresponding author:
Marta Blangiardo
Imperial College London, St Mary's Campus,
Norfolk Place, W2 1PG
m.blangiardo@imperial.ac.uk

Keywords:
life expectancy, anomaly detection, area-level deprivation, spatio-temporal Bayesian model

Words: 3192

For peer review only

Abstract

Objectives

To estimate life expectancy at the local authority level and detect those areas that have a substantially low life expectancy after accounting for deprivation.

Design

We used registration data from the Office for National Statistics (ONS) on mortality and population in England, by local authority, age group, and deprivation decile, for both males and females over the period 2001-2018. We used a statistical model within the Bayesian framework to produce robust mortality rates, which were then transformed to life expectancy estimates. A rule based on exceedance probabilities was used to detect local authorities characterised by a low life expectancy among areas with a similar deprivation level from 2012 onwards.

Results

We confirmed previous findings showing differences in the life expectancy gap between the most and least deprived areas from 2012 to 2018. We found variations in life expectancy trends across local authorities, and we detected a number of those with a low life expectancy when compared to others of a similar deprivation level.

Conclusions

There are factors other than deprivation that are responsible for low life expectancy in certain local authorities. Further investigation on the detected areas can help with understanding better the stalling of life expectancy which was observed from 2012 onwards and help with efficient public health policies.

Strengths and limitations of this study

- This is the first study of its scale, including more than 8 million records.
- The methodology produces robust estimates via the Bayesian hierarchical framework used.
- Through the detection of local authorities that deviate from the expected trend, our framework help better understand the stalling of life expectancy in order to implement efficient public health measures.
- It was not possible to account for population changes due to migration in the model.
- The socio-economic indicator that was used is based on data from the last available year; we performed sensitivity analysis using the mid-year deprivation indicator for the period considered.

Introduction

The study of life expectancy is of primary interest in public health practice, where it is used to measure the overall health of a population, and also to plan for health and social service provision. While life expectancy has been improving steadily over the last decades, mainly due to changes in behavioural risk factors and healthcare, this phenomenon has started to slow down from 2012 onwards and has become cause of concern in several country, including the USA and the UK.[1,2]

For peer review only

Possible explanations of this include strikes in influenza among older people, potentially due to ineffective vaccines,[3] or increases in mortality for mental health conditions, such as dementia and Alzheimer's disease.[4] Recent studies in the UK found that the excess number of deaths in 2015 were largely driven by the older populations and suggested potential associations with socioeconomic factors, poor health and social care, which can be linked to reductions in spending across the healthcare system.[5,6,7]

Socio-economic deprivation has been found to be the major determinant of life expectancy by numerous studies over the years, regardless of the measure of deprivation that was used. [8, 9, 10, 11, 12, 13] Geographical patterns of life expectancy in England and Wales were found to be mainly attributable to variations in deprivation status in 1998,[8] while a more recent study found that the elimination of socio-economic differences between areas would increase survival among older adults across a number of European countries, with the strongest association in England.[9] The impact of deprivation on overall levels of life expectancy is therefore established. However, the corresponding impact on life expectancy improvements over time is less clear; an early paper found that in most deprived areas improvements in life expectancy were negligible,[10] while a more recent paper showed that the life expectancy gap between the most affluent and most deprived areas increased from 2001 to 2016 for both sexes, with a higher magnitude for females.[11] This finding was not confirmed by later studies where largest improvements in life expectancy were documented in some of the most deprived areas.[12] Recent life expectancy trends have been studied in comparison to socio-economic deprivation, mostly at the decile level, by aggregating areas in England into deciles according to their measure of area deprivation.[11,15,16,17] Only limited research has investigated relevant associations at the smaller area level, including a study in Scotland[18] and one in Sweden.[19]

The majority of previous studies have used a low geographical resolution. It is nevertheless crucial to focus on small regions and detect those that are most in need, as this would help better understand the life expectancy gap and consequently support governments with evidence for prioritisation and more efficient public health policies. This cannot rely solely on data description, as splitting the number of deaths by a combination of small area, age, gender and year leads to low number of cases and consequently to unstable mortality rates and life expectancy estimates. The need for a modelling approach has been highlighted before to overcome this issue.[20] Additionally, we argue that it is important to establish the role of deprivation in detecting areas performing poorly in terms of life expectancy, as well as to detect those areas that differ from others with a similar level of socio-economic deprivation and show unexplained lower life expectancy. These regions should be investigated further to understand which factors are driving the stalling of life expectancy, in order to direct policies and intervention measures.

In this paper, we analyse all-cause mortality counts in England at the local authority level from 2001 to 2018 in order to assess the impact of deprivation on life expectancy improvements over time. Moreover, focusing on 2012-2018 when the stalling effect was observed, we detect areas which have shown lower life expectancy than other areas characterised by a similar level of social deprivation.

Methods

Data

We used mortality counts from 2001 to 2018 at the local authority level in England. The local authority level is an administrative subdivision of England with population size ranging from 2.3 thousand (Isle of Scilly) to 1.6 million (Kent County Council). In total 315 local authorities were considered. Information on age and sex was available for each record. We considered 20 age groups, each including 5 years except for the first (< 1 year old) and the last group (90 and older) and we analysed males and females separately as these have previously shown to have very

For peer review only - http://bmjopen.bmj.com/site/about/guidelines.xhtml

different mortality levels and trends.[21] Population data by age and sex, as well as a measure of deprivation for each local authority were also used for the analysis. All data were provided by Public Health England (PHE), originally held by Office for National Statistics (ONS).

Deprivation data were available at the decile level. In this study we used the English index of multiple deprivation (IMD), based on seven different domains of deprivation: i) income deprivation, ii) employment deprivation, iii) education, skills and training deprivation, iv) health deprivation and disability, v) crime, vi) barriers to housing and services, and vii) living environment deprivation. Each local authority was allocated to a deprivation decile (D1, …, D10) with D1 being the most deprived and D10 the least deprived decile based on the IMD 2019.[22]

Patient and public involvement

As this is secondary research based on aggregated data that have already been collected, no patients or the public were involved in designing the study, nor were they involved in the analysis or implementation of the study. Dissemination to study participants or patient organisations is not applicable.

Statistical analysis

We developed a statistical model within a Bayesian hierarchical framework; we specified a Poisson distribution for the mortality counts (y_{ijtd}) by age group (*j=1,…,20*), local authority (*i=1,…,315*), calendar year (*t=1,…,18*), and deprivation decile (*d=1,…,10*):

$$y_{ijtd} \sim \text{Poisson}\ (\lambda_{ijtd} \times Pop_{ijt})$$

where Pop_{ijt} is the population and λ_{ijtd} the mortality rate. Borrowing from disease mapping literature, the log transformed rate is then modelled as the linear combination of several terms as follows: [23,24]

$$\log(\lambda_{ijtd}) = a_0 + \alpha_j + \gamma_i + \zeta_t + \phi_d + \omega_{it} + \delta_{ij} + \kappa_{jt} + \nu_{dt}$$

where $\alpha_j, \gamma_i, \zeta_t$ and ϕ_d are random effects identifying the main effects of age, local authority, year and deprivation deciles. Pairwise interactions between age and space (δ_{ij}), age and time (κ_{jt}), space and time (ω_{it}), and deprivation and time (ν_{dt}) are also included to allow for deviation from the main effects and to increase the level of flexibility of the model.

The Bayesian framework allows to incorporate assumptions regarding the structure of the mortality rates via prior distributions on the parameters. It is known that mortality rates vary slowly with age, time, and to some extent across space and it is reasonable to expect that their trends have a smooth pattern. We therefore specify a random walk prior of order 1 (RW1) for the age-, time-, and deprivation-specific components α_j, ζ_t and ϕ_d, assuming that units that are consecutive in terms of age, time or deprivation decile have similar mortality rates. The spatial component γ_i is assigned a conditional autoregressive (CAR) prior, assuming that areas neighboring each other share similar characteristics in terms of mortality rates.[25] We assume similarity across time and decile of deprivation for each age group, specifying a RW1 on the corresponding interaction terms, κ_{jt} and ν_{dt}. On the other hand, the interaction of local authority-age, and local authority- year are left unstructured and data driven, through an exchangeable Normal prior on δ_{ij} and ω_{it}. The overall intercept a_0 is assigned a noninformative Normal prior.

For peer review only

A rule to detect local authorities characterised by lower life expectancy is specified based on the space-time interaction term of the model ω_{it}, that, if above zero, would suggest a local increase of mortality rates. We estimate the probability that the space-time interaction term is above zero for all space-time combinations and we select the local authorities with such probability above 0.95. We restrict the detection on 2012-2018 to focus on the period characterised by the stalling of life expectancy. Further details on the model and the code for running it are available at https://github.com/aretib/LifeExpectancyDetect.

Results are presented as mean estimates and 95% credible intervals (CI). Mortality rates were transformed to life expectancy estimates for each local authority using life tables methods.[21] More details about the procedure to go from mortality rates to life expectancy are reported in https://github.com/aretib/LifeExpectancyDetect.

To evaluate the impact that a change in the definition of the deprivation index has on the detection, we present the results of the same model, where deprivation deciles are based on IMD 2010 (Supplementary Material).

Results

Life expectancy at birth in England was estimated to be 79.6 years for men and 83.2 years for women in 2018. Over the period 2001 to 2018, life expectancy followed an overall increasing trend, with a flatter pattern from 2012 onwards for both genders, with the gap between women and men decreasing over time. Figure 1 shows the time trends of life expectancy by deprivation decile group for men and women respectively. It is observed that the overall level of life expectancy is different across deciles, with higher deprivation corresponding to lower life expectancy. Additionally, it can be noted that life expectancy follows an overall increasing trend for all deciles, however more deprived groups are characterised by flatter patterns compared to less deprived groups.

Focusing on 2012-2018, Figure 2 shows the gap in life expectancy between the beginning and the end of the period and their corresponding 95% CI's. There is a difference of more than 100 days in life expectancy gains for the least deprived decile compared to the most deprived one for both sexes, while it is worth noting the large uncertainty across all the deciles (95% credibility intervals depicted by the error bars). When comparing males and females, we observe bigger gains for males across most deprivation deciles, however there is an overlap in the uncertainties of those two; these findings are in line with reports from ONS and PHE.[16,21]

We found variations in life expectancy trends across local authorities, and a number of those were detected as atypical (i.e. showing lower life expectancy than in the corresponding deprivation decile) from 2012 to 2018. The maps in Figures 3 and 4 show the detected local authorities for males and females respectively with the corresponding number of times that these were picked up out of the 7 year period. In particular, 12 unique areas were detected for females and 18 areas for males (Tables 1 and 2). Looking at the maps, we do not observe any specific spatial pattern in the detected local authorities, as these are randomly scattered across England. From the tables it can be noted that roughly half of the detected local authorities belong to the two most deprived deciles (D1 and D2) for both sexes. Additionally, most local authorities were detected in 1 year only during the 7 year period, with Leeds standing out as it was detected in 2 years for females and 4 years for males. For each detected area, we show mortality rates temporal trends compared those of the corresponding deprivation decile in Supplementary Material (Figures S1-S30).

When running the sensitivity analysis considering the deprivation score from 2010 rather than 2019, we found that the detected areas increase to 31 and females and 25 for males and include all those detected in the main model for both genders. We present the additional areas detected in Table S1

For peer review only

and S2 in Supplementary Material, where we include the posterior probability that ω_{it} is above 0 under the two models. For all the areas, these probabilities are similar and when an area is not detected under the model with IMD 2019, the posterior probability that ω_{it} is above 0 is not far from the 0.95 threshold, which suggests the results are robust. The 19 additional areas detected for females ranges across deprivation deciles and 7 move from a less to a more deprived decile between 2010 and 2019; for males all the additional areas 5 belong to the most deprived deciles and the remaining 2 move from a less to a more deprived deciles between 2010 and 2019.

Discussion

In this analysis we investigated the life expectancy gap between the most and least deprived local authorities in England in order to inform policy implementation and prioritisation. We proposed a statistical tool to look at the life expectancy trends across local authorities and to detect those characterised by lower levels which should be further investigated in order to provide targeted policy measures.

Principal findings

Our analyses suggest that the increase in life expectancy in England from 2012 to 2018 has been greatest in areas with low levels of deprivation, with more modest increases in life expectancy in areas with high levels of deprivation.[13,21] More specifically, the gain in life expectancy in the wealthiest areas (decile 10) compared to the poorest areas (decile 1) in England was more than 100 days for males, and roughly 80 days for females. The corresponding differences among deprivation deciles 2 to 9 were less clear considering the uncertainty around the estimates.

We found that the life expectancy trends did not follow the same pattern across all local authorities for both males and females between 2012 and 2018. More specifically, we detected the local authorities which depicted a lower life expectancy compared to what would have been expected given their deprivation level. This suggests factors other than deprivation that are potentially responsible for the poor performance of the specific local authorities and would call for further investigation by public health bodies and therefore prioritisation for interventions.

We note here that our study is descriptive as it does not formally assess potential causes in the detected areas. Nevertheless, we expect that it will inform the evidence around life expectancy gaps and the role of deprivation; this should be used as the basis for future studies targeting the detected local authorities to investigate potential responsible factors, to be conducted in collaboration with epidemiologists and public health researchers.

The follow up study on the detected areas should consider disentangling the different domains of socio-economic deprivation, such as barriers to housing and services, or health deprivation and disability, to see if any of these domains is dragging the life expectancy of an otherwise non-deprived local authority down. Austerity has been linked to the stalling of life expectancy;[26] this is another aspect which should be further investigated, particularly looking at how different cities have implemented specific local policies related to austerity which might have contributed to the different life expectancy trends. Furthermore, it would worth investigating the different causes of death separately to help with hypothesis making; for example, if the low life expectancy of an area is attributed to teenage suicides, this might indicate unprotected environments in specific schools of that area. Finally, any events or changes in a neighbourhood should be explored to assess any potential impact these might have on their life expectancy; as an example, if a hospital that provides long-term care is built in an area, that area will experience unusually low life expectancy.

Comparison to previous studies

Our life expectancy estimates are in line with the ONS estimates for both males and females. [21] In

For peer review only

addition, we show that deprivation has an impact not only on the overall life expectancy estimates but also on life expectancy improvements.[16] This is in line with an earlier study investigating the impact of a health inequalities intervention, implemented by the UK government between 1997 and 2010; the authors find that the life expectancy gap between the population that lives in the 20% most deprived local authorities and the rest of the population declined during the intervention, while this trend is reversed after the intervention ended in 2010.[27]

Differently from Bennett and others who run the analyses at the deprivation decile, the novelty of our study lies on the analysis of life expectancy at the local authority level, while accounting for deprivation. [13] This allows us to propose a mechanism to detect local authorities in England with lower life expectancy for their deprivation level and therefore provide a first step in the investigation and understanding of factors other than deprivation that might be responsible in order to develop targeted interventions.

Strengths and limitations

Our analysis of mortality registration data in England from 2001 to 2018 has a number of advantages. Firstly, to our knowledge, it is the first study of its scale, investigating life expectancy at the local authority level in the whole of England over an 18-year period, including more than 8 million records. Second, the methodology that we use, appropriate for the small numbers that arise when mortality counts are considered by local authority, year, and age group, produces robust estimates, unlike the standard statistical techniques frequently used for this type of analysis.[28] Third, we are able to detect specific local authorities in England which are most left behind in terms of life expectancy improvements, addressing an important gap in the literature. We finally propose a statistical tool that can be used on a routine basis for detection of life expectancy anomalies in England while accounting for deprivation. While a segment tool is available by PHE to provide information on the life expectancy gap at the local authority level,[29] this is based on crude mortality rates and therefore less stable compared to the smoothed ones produced through statistical modelling, it provides mean estimates over 2015-2017 and not annual estimates, and most importantly it is not able to automatically flag areas which would require further investigation, but one should rather examine these individually.

Limitations of our study include population changes because of migration, both within the country and overseas, which are not accounted for in the model. In addition, the allocation of local authorities into discrete groups of deprivation, will inevitably yield some variation within local authorities, since for example not all people can be of the same socio-economic status, which is a common issue in ecological studies. Lastly, the socio-economic indicator that we use is based on data from a specific year and does not account for changes in the local authorities between deprivation deciles over the period under study. We chose the deprivation index at 2019 as it provides the latest estimate of deprivation at small area. Additionally, as the data used to construct the index are related to the period 2013-2017, it is the best representation of deprivation in your detection period (2012-2018). Nevertheless, in our sensitivity analysis we were able to detect all the areas originally detected in the main model and we show that the results are robust, when comparing the posterior probability that ω_{it} is above 0.

Conclusions

Much research has recently focused on life expectancy to understand the underlying causes of the time trends observed in England. Although these shed light into potential factors that are driving these, we believe it is of high importance to use a detection mechanism as part of a routine monitoring surveillance in order to focus on specific areas that are most in need.

Detection of specific local authorities in England, while accounting for socio-economic deprivation, would highlight those areas that perform worse compared to others of a similar deprivation level.

For peer review only

Our study contributes to the state of the art by proposing a statistical tool to perform this type of anomaly detection.

Footnotes

Contributors

Both authors contributed to the design of the study, statistical analyses, interpretation and manuscript writing. The corresponding author attests that all listed authors meet authorship criteria and that no others meeting the criteria have been omitted.

Acknowledgements

The authors would like to thank Garyfallos Konstantinoudis, Monica Pirani and Chiara Forlani from Imperial College London and Allan Baker, Paul Fryers from PHE for their constructive comments which helped improve the paper. Areti Boulieri acknowledges support from an MRC Early Career Research Fellowship (MR/M501669/1). Marta Blangiardo acknowledges support by Public Health England award titled "Assessment of Trends in Life Expectancy in England and its Upper-tier Local authorities".

Competing interests

None.

Ethical approval

Not required.

Data sharing

The data used for the study were provided by Public Health England (PHE) and cannot be shared online due to confidentiality.

Transparency declaration

The lead author (AB) affirms that the manuscript is an honest, accurate, and transparent account of the study being reported; that no important aspects of the study have been omitted; and that any discrepancies from the study as planned have been explained.

For peer review only

327 **Tables**

328

Local authority	Decile	Year
Kingston upon Hull, City of	D1	2017
Stoke-on-Trent	D1	2018
Tameside	D1	2018
Newcastle upon Tyne	D2	2015
Plymouth	D2	2017
Leeds	D2	2015, 2018
Derby	D3	2018
County Durham	D4	2013
Cornwall inc Isles of Scilly	D4	2016
Bournemouth, Christchurch and Poole	D6	2015, 2017
Stroud	D9	2013
Sutton	D8	2014

329

330 *Table 1: Local authorities with a low life expectancy for their deprivation level for females*

331

332

For peer review only

Local authority	Decile	Year
Middlesbrough	D1	2017
Wolverhampton	D1	2018
Kingston upon Hull, City of	D1	2017
Birmingham	D1	2014
Tendring	D2	2017
Leeds	D2	2012, 2015, 2017, 2018
Newcastle upon Tyne	D2	2014
Southampton	D2	2013
Bristol, City of	D3	2013, 2018
Cornwall inc Isles of Scilly	D4	2018
Breckland	D5	2018
Ashford	D5	2018
Hillingdon	D5	2018
Cheshire West and Chester	D6	2016
Dorset Council	D7	2016
Teignbridge	D7	2012
Stroud	D9	2016
Bath and North East Somerset	D9	2016

Table 2: Local authorities with a low life expectancy for their deprivation level for males

For peer review only

Figure legends

Figure 1: Life expectancy estimates from 2001 to 2018 across deprivation deciles; the shaded areas correspond to 95% CI's

Figure 2: Life expectancy gap between 2012-2013 and 2017-2018; the error bars correspond to 95% CI's

Figure 3: Local authorities detected with a lower life expectancy than others with a similar deprivation level for females

Figure 4: Local authorities detected with a lower life expectancy than others with a similar deprivation level for males

For peer review only

References

[1] Olshansky, S Jay, Douglas J Passaro, Ronald C Hershow, Jennifer Layden, Bruce A Carnes, Jacob Brody, Leonard Hayflick, Robert N Butler, David B Allison, and David S Ludwig. 2005. "A Potential Decline in Life Expectancy in the United States in the 21st Century." *New England Journal of Medicine* 352 (11): 1138–45.

[2] Hiam, Lucinda, Dominic Harrison, Martin McKee, and Danny Dorling. 2018. "Why Is Life Expectancy in England and Wales 'Stalling'?" *Journal of Epidemiology and Community Health* 72 (5): 404–8.

[3] Pebody, RG, HK Green, F Warburton, M Sinnathamby, J Ellis, K Mølbak, J Nielsen, S de Lusignan, and N Andrews. 2018. "Significant Spike in Excess Mortality in England in Winter 2014/15–Influenza the Likely Culprit." *Epidemiology & Infection*, 1–8.

[4] Liddell, Christine, Chris Morris, Barbara Gray, Anna Czerwinska, and Ben Thomas. 2016. "Excess Winter Mortality Associated with Alzheimer's Disease and Related Dementias in the Uk: A Case for Energy Justice." *Energy Research & Social Science* 11: 256–62.

[5] Hiam, Lucinda, Danny Dorling, Dominic Harrison, and Martin McKee. 2017a. "What Caused the Spike in Mortality in England and Wales in January 2015?" *Journal of the Royal Society of Medicine* 110 (4): 131–37.

[6] Hiam, Lucinda, Danny Dorling, Dominic Harrison, and Martin McKee. 2017b. "Why Has Mortality in England and Wales Been Increasing? An Iterative Demographic Analysis." *Journal of the Royal Society of Medicine* 110 (4): 153–62.

[7] Watkins, Johnathan, Wahyu Wulaningsih, Charlie Da Zhou, Dominic C Marshall, Guia DC Sylianteng, Phyllis G Dela Rosa, Viveka A Miguel, Rosalind Raine, Lawrence P King, and Mahiben Maruthappu. 2017. "Effects of Health and Social Care Spending Constraints on Mortality in England: A Time Trend Analysis." *BMJ Open* 7 (11): e017722.

[8] Woods, Laura M, Bernard Rachet, Michael Riga, Noell Stone, Anjali Shah, and Michel P Coleman. 2005. "Geographical Variation in Life Expectancy at Birth in England and Wales Is Largely Explained by Deprivation." *Journal of Epidemiology & Community Health* 59 (2): 115–20.

[9] Ribeiro, Ana Isabel, Elias Teixeira Krainski, Marilia Sá Carvalho, Guy Launoy, Carole Pornet, and Maria de Fátima de Pina. 2018. "Does Community Deprivation Determine Longevity After the Age of 75? A Cross-National Analysis." *International Journal of Public Health* 63 (4): 469–79.

[10] Raleigh, Veena Soni, and Victor A Kiri. 1997. "Life Expectancy in England: Variations and Trends by Gender, Health Authority, and Level of Deprivation." *Journal of Epidemiology & Community Health* 51 (6): 649–58.

[11] Bennett, James E, Jonathan Pearson-Stuttard, Vasilis Kontis, Simon Capewell, Ingrid Wolfe, and Majid Ezzati. 2018. "Contributions of Diseases and Injuries to Widening Life Expectancy Inequalities in England from 2001 to 2016: A Population-Based Analysis of Vital Registration Data." *The Lancet Public Health* 3 (12): e586–e597.

[12] Kim, J.I. and Kim, G. 2018. "Effects on inequality in life expectancy from a social ecology perspective." *BMC public health*, *18*(1):243.

[13] Kim, J.I. and Kim, G. 2014. Factors affecting the survival probability of becoming a centenarian for those aged 70, based on the human mortality database: income, health expenditure, telephone, and sanitation. *BMC geriatrics*, *14*(1):113.

[14] Public Health England. 2019a. "Recent Trends in Mortality in England: Review and Data

Packs. A Report on Recent Trends in Life Expectancy and Mortality in England." https://www.gov.uk/government/publications/recent-trends-in-mortality-in-england-review-and-data-packs.

[15] Mayhew, Les, Gillian Harper, and Andrés M Villegas. 2020. "An Investigation into the Impact of Deprivation on Demographic Inequalities in Adults." *Annals of Actuarial Science*, doi: 10.1017/S1748499520000068.

[16] Public Health England. 2018. "Health Profile for England: 2018." https://www.gov.uk/government/publications/health-profile-for-england-2018.

[17] Iacobucci, Gareth. 2019. "Life Expectancy Gap Between Rich and Poor in England Widens." *British Medical Journal*, 364:l1492.

[18] Seaman, Rosie, Tim Riffe, Alastair H Leyland, Frank Popham, and Alyson van Raalte. 2019. "The Increasing Lifespan Variation Gradient by Area-Level Deprivation: A Decomposition Analysis of Scotland 1981–2011." *Social Science & Medicine* 230: 147–57.

[19] Åström, Daniel Oudin, Paul W Franks, and Kristina Sundquist. 2018. "Neighborhoods and Mortality in Sweden: Is Deprivation Best Assessed Nationally or Regionally?" *Demographic Research* 38: 429–50.

[20] Newton, John N. 2018. "Dissecting the Life Expectancy Gap in England Provides Clues on How to Reduce It." *The Lancet Public Health* 3 (12): e560–e561.

[21] Office for National Statistics. 2019a. "National Life Tables, UK: 2016 to 2018." https://www.ons.gov.uk/releases/nationallifetablesuk2016to2018.

[22] Office for National Statistics. 2019b. "Data: National Life Tables, England." https://www.ons.gov.uk/peoplepopulationandcommunity/birthsdeathsandmarriages/lifeexpectancies/datasets/nationallifetablesenglandreferencetables.

[23] Lawson A, Lee D. 2017 Bayesian disease mapping for public health. In: *Handbook of statistics*. Elsevier. 443–81.

[24] Best N, Richardson S, Thomson A. 2005. A comparison of Bayesian spatial models for disease mapping. *Statistical Methods in Medical Research*; 14:35–59.

[25] Besag J, York J, Mollié A. 1991. Bayesian image restoration, with two applications in spatial statistics. *Annals of the Institute of Statistical Mathematics*; 43:1–20.

[26] Christie, Bryan. 2019. "Study Cites Austerity as Factor in Stalling of Life Expectancy in Rich Countries." *British Medical Journal* 364:l645.

[27] Barr, Ben, James Higgerson, and Margaret Whitehead. 2017. "Investigating the Impact of the English Health Inequalities Strategy: Time Trend Analysis." *British Medical Journal* 358: j3310.

[28] White, Chris. 2015. "Trend in Life Expectancy at Birth and at Age 65 by Socio-Economic Position Based on the National Statistics Socio-Economic Classification, England and Wales: 1982-1986 to 2007-2011." *Statistical Bulletin*.

[29] Public Health England. 2019b. "Segment Tool: 2019 Data Update."https://www.gov.uk/government/statistics/segment-tool-2019-data-update.

For peer review only

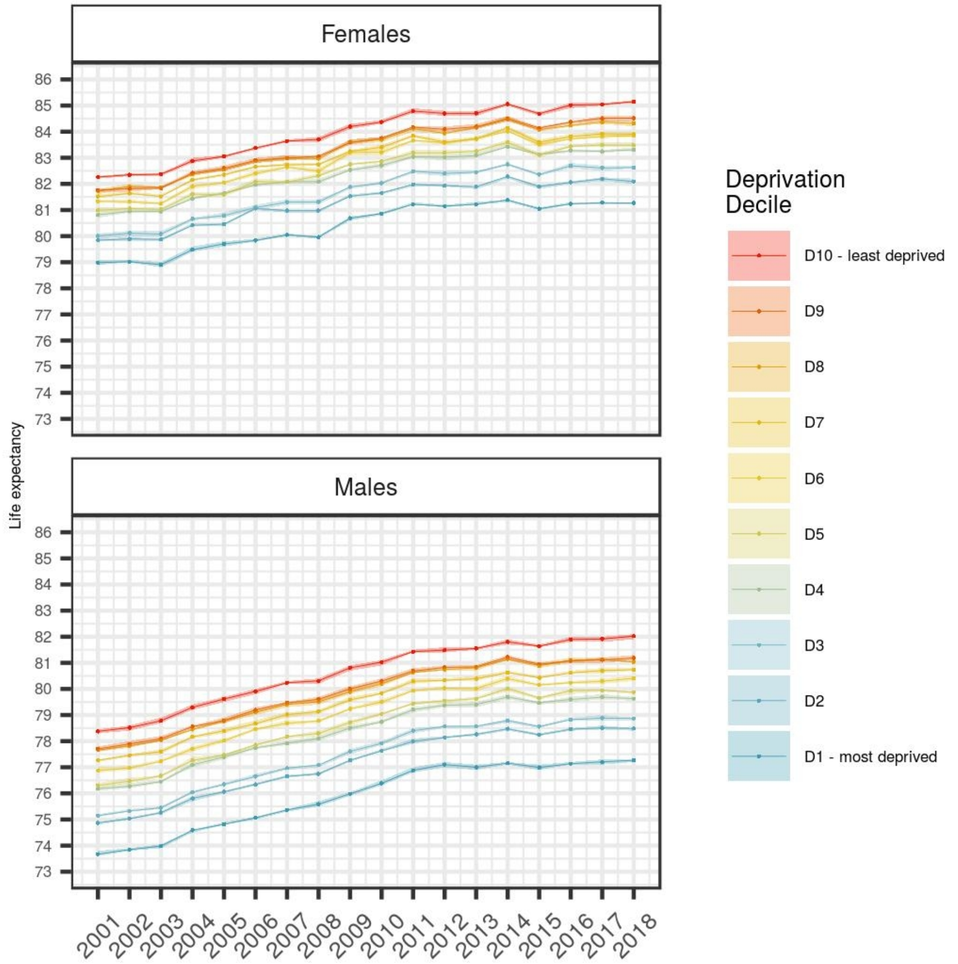

Figure 1: Life expectancy estimates from 2001 to 2018 across deprivation deciles; the shaded areas correspond to 95% CI's

89x89mm (300 x 300 DPI)

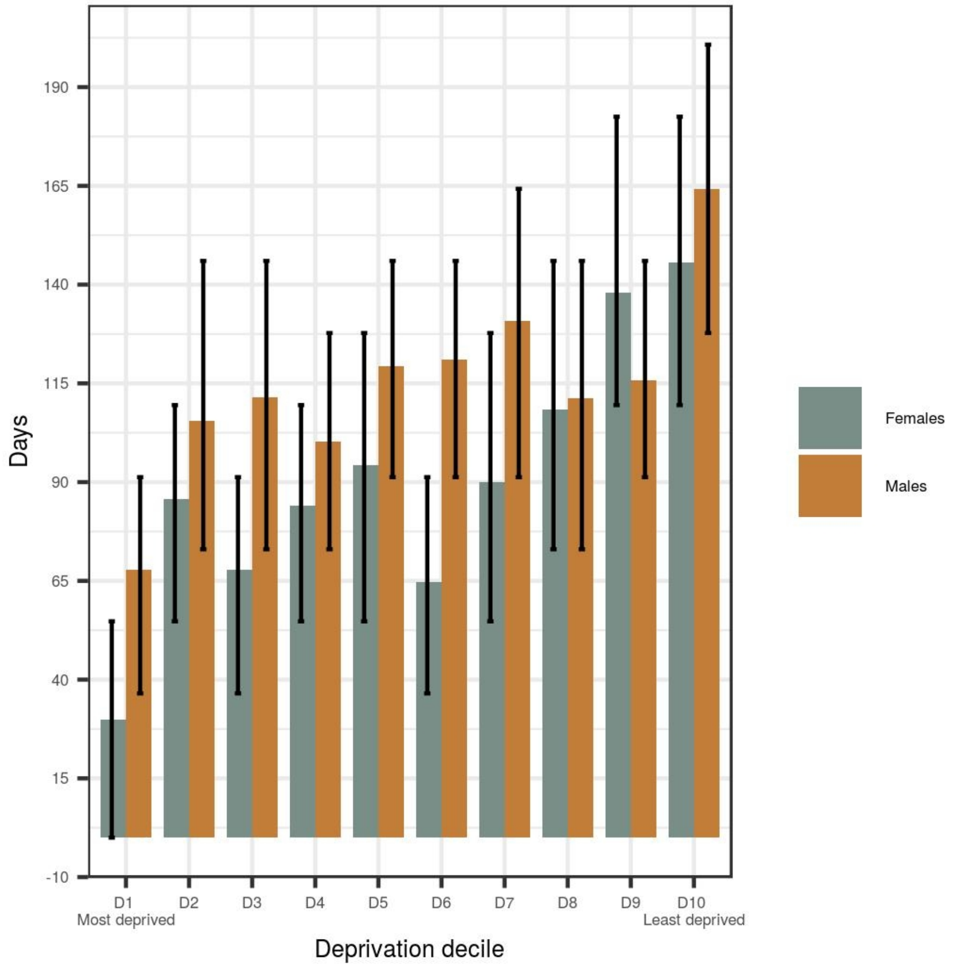

Figure 2: Life expectancy gap between 2012-2013 and 2017-2018; the error bars correspond to 95% CI's

89x89mm (300 x 300 DPI)

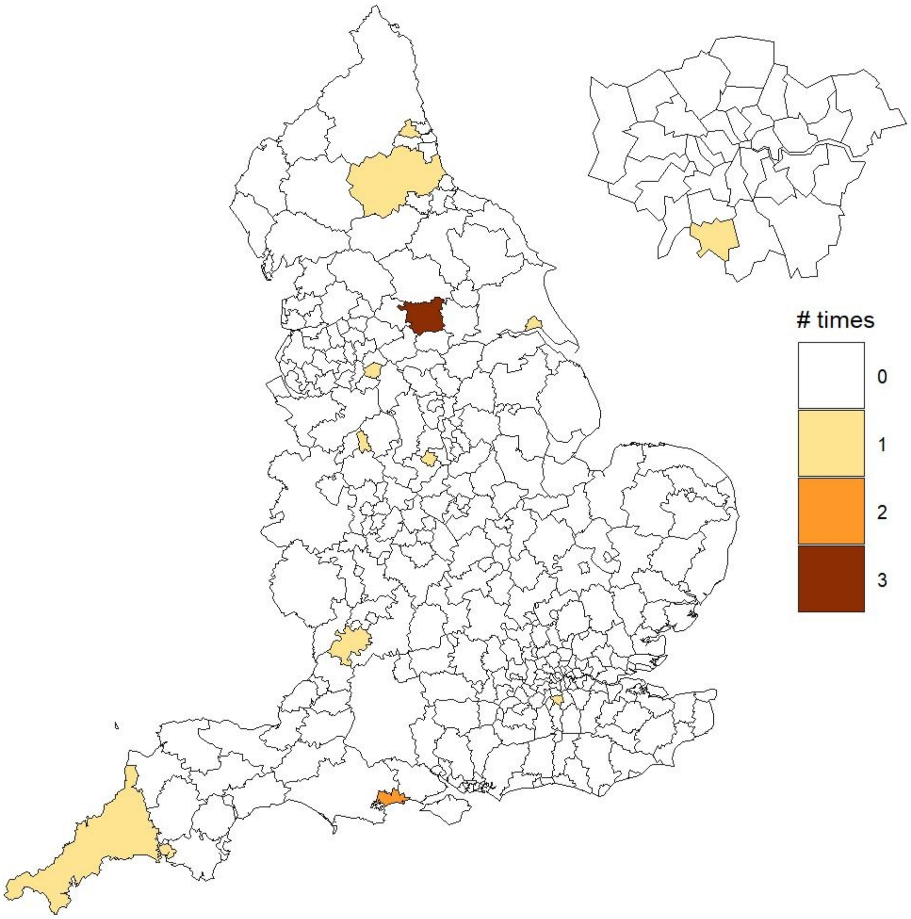

Caption : Figure 3: Local authorities detected with a lower life expectancy than others with a similar deprivation level for females

89x89mm (300 x 300 DPI)

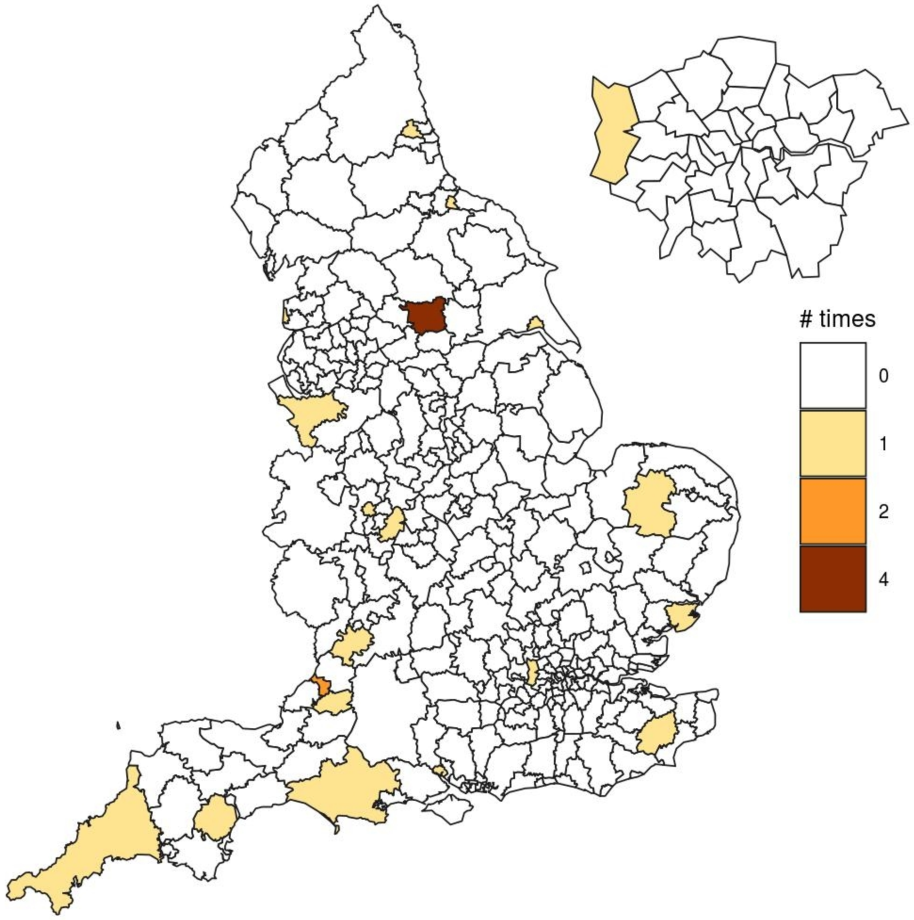

Figure 4: Local authorities detected with a lower life expectancy than others with a similar deprivation level for males

89x89mm (300 x 300 DPI)

Supplementary Material: A spatio-temporal model to estimate life expectancy and to detect unusual trends at the local authority level in England

Areti Boulieri[a], Marta Blangiardo[a*]

[a] Department of Epidemiology and Biostatistics, MRC Centre for Environment and Health, Imperial College London, London, UK

* Corresponding author:
Marta Blangiardo
Imperial College London, St Mary's Campus,
Norfolk Place, W2 1PG
m.blangiardo@imperial.ac.uk

For peer review only

Table S1: : *Local authorities with a low life expectancy for their deprivation level for females under the model with deprivation index at 2010. The areas detected under the model with deprivation index at 2019 are a subset (in bold). We present the posterior probability under the two models.*

		IMD2010		IMD2019	
Local Authority	Year	Decile	Pr(ω_{it}>0.95)	Decile	Pr(ω_{it}>0.95)
Blackpool	2017	D1	0.976	D1	0.912
Blackpool	2018	D1	0.959	D1	0.871
Bradford	2013	D1	0.981	D1	0.941
Kingston upon Hull, City of	2014	D1	0.951	D1	0.881
Kingston upon Hull, City of	2015	D1	0.972	D1	0.914
**Kingston upon Hull, City of**	**2017**	**D1**	**0.997**	**D1**	**0.977**
Kingston upon Hull, City of	2018	D1	0.981	D1	0.915
Manchester	2014	D1	0.960	D1	0.894
Oldham	2014	D1	0.980	D1	0.949
Stoke-on-Trent	2017	D1	0.980	D1	0.913
**Stoke-on-Trent**	**2018**	**D1**	**1.000**	**D1**	**0.999**
**Tameside**	**2018**	**D1**	**0.993**	**D1**	**0.969**
Wolverhampton	2017	D1	0.961	D1	0.864
Wolverhampton	2018	D1	0.991	D1	0.941
**Leeds**	**2013**	**D3**	**0.999**	**D2**	**0.997**
**Leeds**	**2015**	**D3**	**0.981**	**D2**	**0.958**
**Leeds**	**2018**	**D3**	**1.000**	**D2**	**1.000**
**Newcastle upon Tyne**	**2015**	**D2**	**0.974**	**D2**	**0.951**
Mansfield	2017	D2	0.962	D2	0.919
**Plymouth**	**2017**	**D3**	**0.986**	**D2**	**0.970**
King's Lynn and West Norfolk	2017	D3	0.951	D3	0.922
Medway	2014	D3	0.952	D3	0.930
**Derby**	**2018**	**D4**	**0.954**	**D3**	**0.956**
**County Durham**	**2013**	**D3**	**0.989**	**D4**	**0.978**
Cornwall inc Isles of Scilly	2013	D3	0.956	D4	0.923
**Cornwall inc Isles of Scilly**	**2016**	**D3**	**0.974**	**D4**	**0.964**
North Tyneside	2017	D4	0.954	D4	0.925
Breckland	2015	D5	0.956	D5	0.912
Stockport	2013	D5	0.967	D5	0.942
**Bournemouth, Christchurch and Poole**	**2015**	**D3**	**0.998**	**D6**	**0.996**
Bournemouth, Christchurch and Poole	2016	D3	0.959	D6	0.941
**Bournemouth, Christchurch and Poole**	**2017**	**D3**	**0.975**	**D6**	**0.963**
Colchester	2014	D7	0.959	D6	0.925
Colchester	2017	D7	0.970	D6	0.939
East Riding of Yorkshire	2012	D7	0.967	D7	0.931

For peer review only

Epping Forest	2014	D7	0.955	D7	0.905
Dorset Council	2016	D7	0.982	D7	0.946
North Somerset	2015	D7	0.967	D7	0.920
Chelmsford	2017	D6	0.965	D8	0.908
**Sutton**	**2014**	**D6**	**0.974**	**D8**	**0.954**
Tonbridge and Malling	2018	D8	0.954	D8	0.889
**Stroud**	**2013**	**D8**	**0.977**	**D9**	**0.954**
Stroud	2017	D8	0.952	D9	0.932
Harrogate	2014	D9	0.951	D9	0.901

For peer review only

Table S2: *Local authorities with a low life expectancy for their deprivation level for males under the model with deprivation index at 2010. The areas detected under the model with deprivation index at 2019 are a subset (in bold). We present the posterior probability under the two models.*

		IMD2010		IMD2019	
Local Authority	Year	Decile	Pr(ω_{it}>0.95)	Decile	Pr(ω_{it}>0.95)
Middlesbrough	2014	D1	0.962	D1	0.944
**Middlesbrough**	**2017**	**D1**	**0.989**	**D1**	**0.976**
**Blackpool**	**2015**	**D1**	**0.993**	**D1**	**0.985**
Blackpool	2018	D1	0.952	D1	0.896
Kingston upon Hull, City of	2016	D1	0.955	D1	0.892
**Kingston upon Hull, City of**	**2017**	**D1**	**1.000**	**D1**	**0.998**
Leicester	2015	D1	0.953	D1	0.902
Leicester	2017	D1	0.960	D1	0.891
Manchester	2014	D1	0.970	D1	0.933
Stoke-on-Trent	2018	D1	0.974	D1	0.918
South Tyneside	2018	D2	0.967	D1	0.945
**Birmingham**	**2014**	**D1**	**1.000**	**D1**	**0.996**
Walsall	2015	D2	0.970	D1	0.933
Walsall	2017	D2	0.974	D1	0.920
**Wolverhampton**	**2018**	**D1**	**0.995**	**D1**	**0.978**
Bradford	2016	D1	0.965	D1	0.870
**Leeds**	**2015**	**D3**	**0.980**	**D2**	**0.985**
**Newcastle upon Tyne**	**2014**	**D2**	**0.967**	**D2**	**0.970**
**Southampton**	**2013**	**D3**	**0.974**	**D2**	**0.977**
**Tendring**	**2017**	**D3**	**0.961**	**D2**	**0.967**
**Bristol, City of**	**2013**	**D3**	**0.975**	**D3**	**0.985**
**Bristol, City of**	**2018**	**D3**	**0.977**	**D3**	**0.990**
**Cornwall inc Isles of Scilly**	**2018**	**D3**	**0.995**	**D4**	**0.993**
**Ashford**	**2018**	**D6**	**0.957**	**D5**	**0.958**
**Breckland**	**2018**	**D6**	**0.995**	**D5**	**0.995**
**Hillingdon**	**2018**	**D4**	**0.951**	**D5**	**0.959**
Cherwell	2014	D8	0.952	D7	0.942
**Dorset Council**	**2016**	**D7**	**0.963**	**D7**	**0.965**
**Teignbridge**	**2012**	**D6**	**0.965**	**D7**	**0.964**
**Stroud**	**2016**	**D8**	**0.975**	**D9**	**0.976**
**Bath and North East Somerset**	**2016**	**D9**	**0.966**	**D9**	**0.967**

For peer review only

Mortality rates for the detected areas under the main model and comparison against the corresponding decile (IMD2019), females

Figure S1: Kingston upon Hull, females

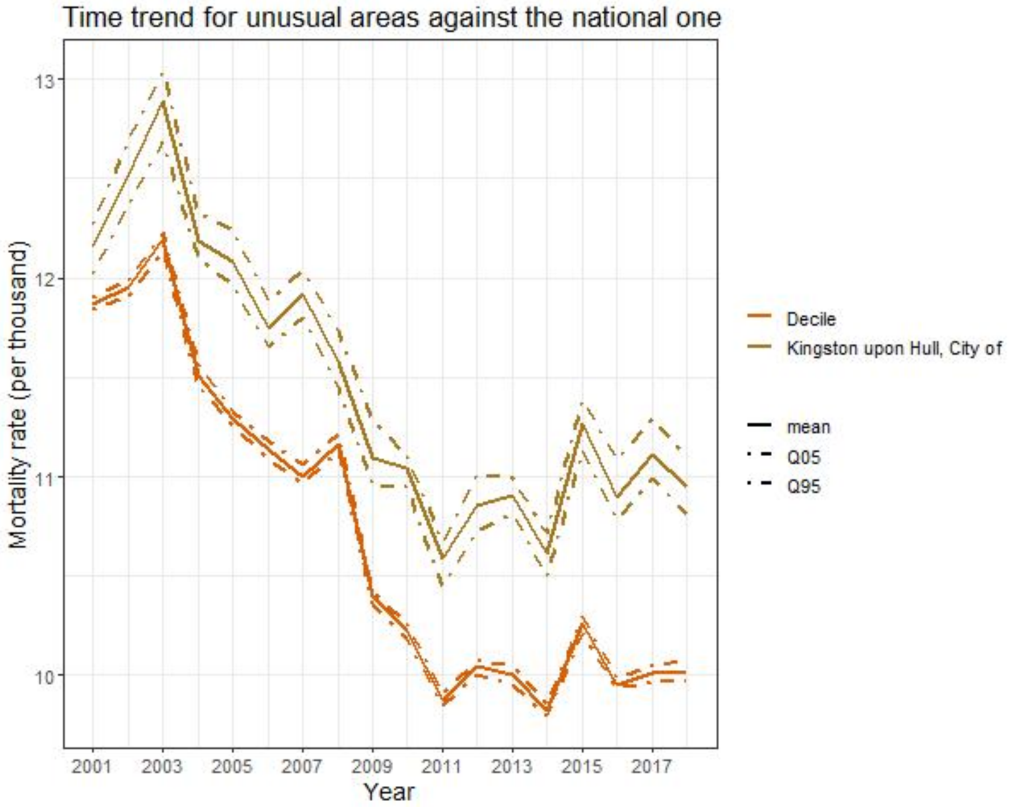

Figure S2: Stoke-on-Trent, females

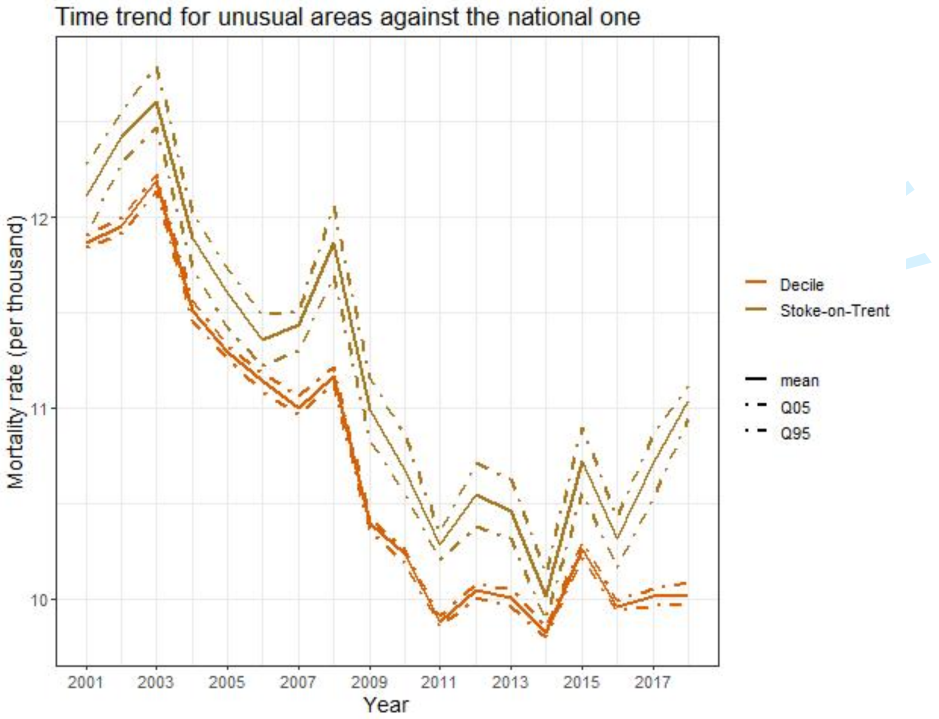

Figure S3: Tameside, females

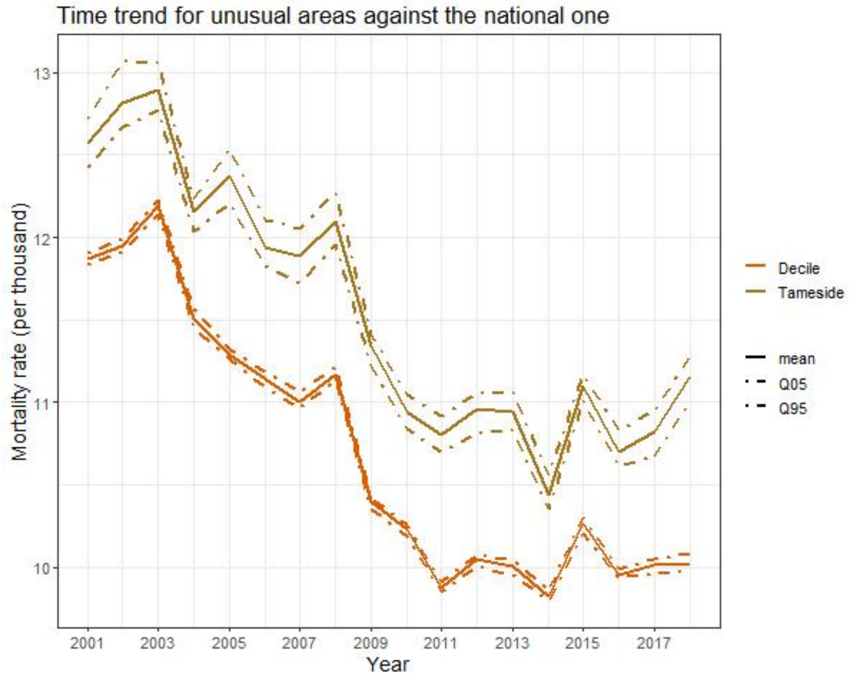

Figure S4: Newcastle upon Tyne, females

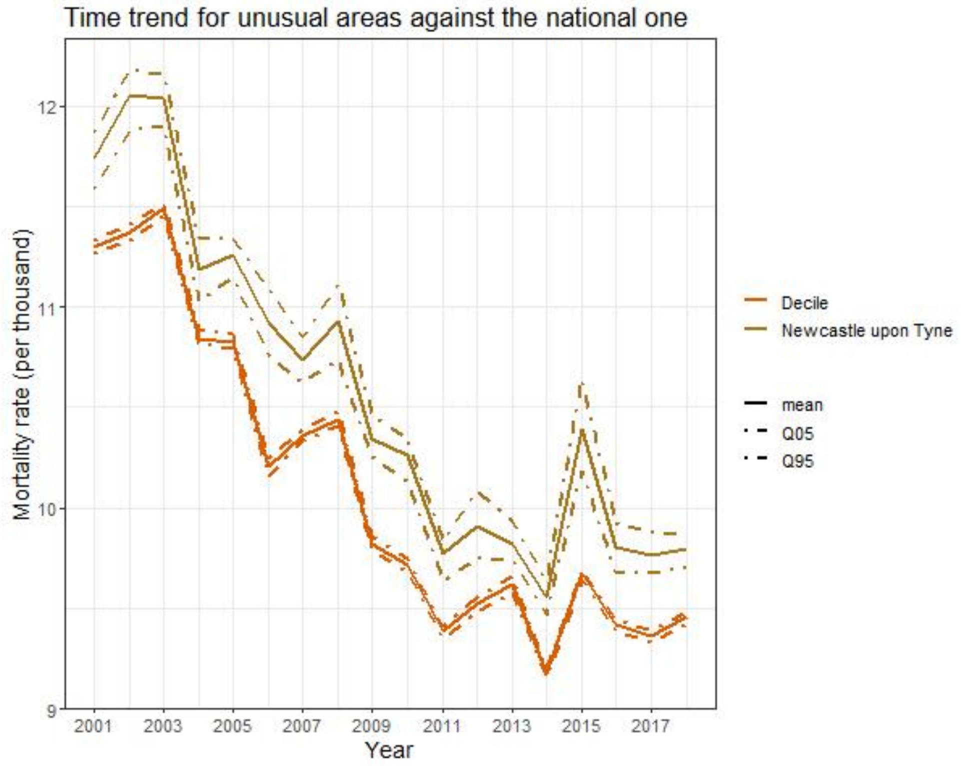

Figure S5: Plymouth, females

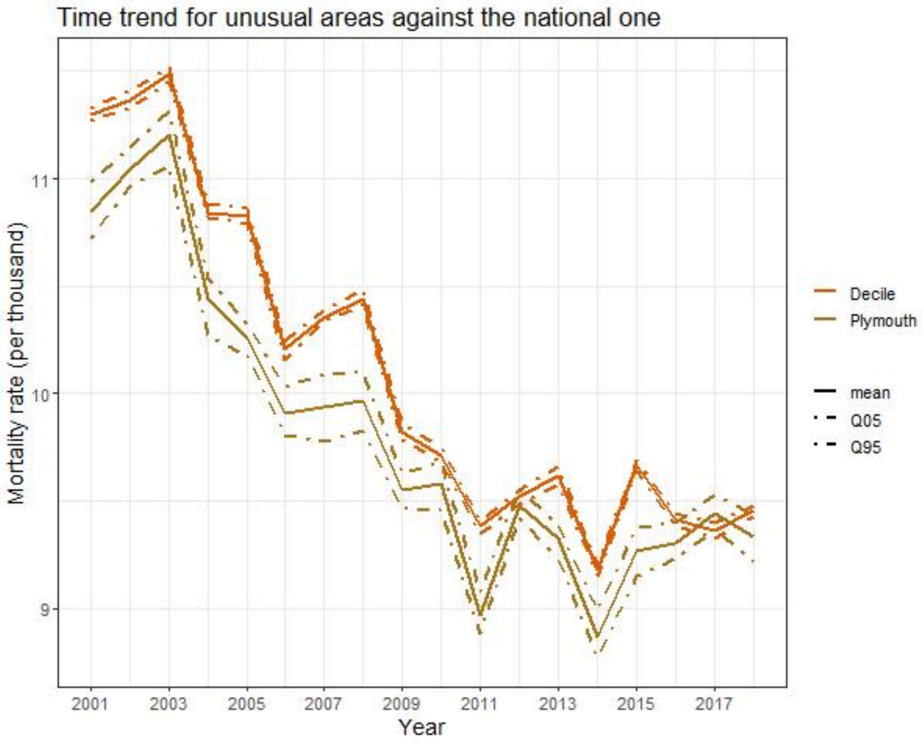

Figure S6: Leeds, females

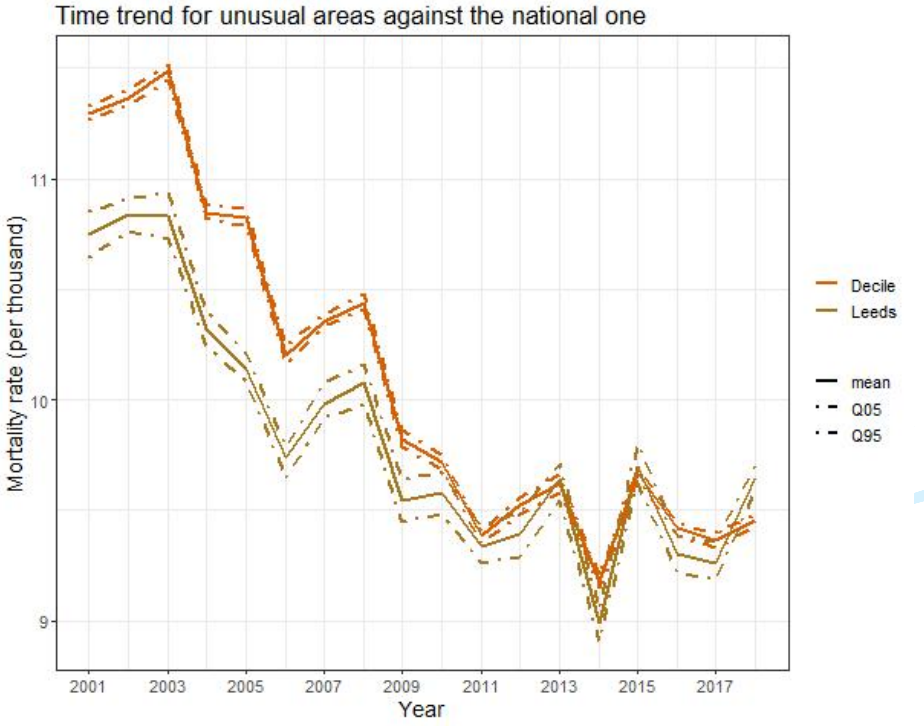

Figure S7: Derby, females

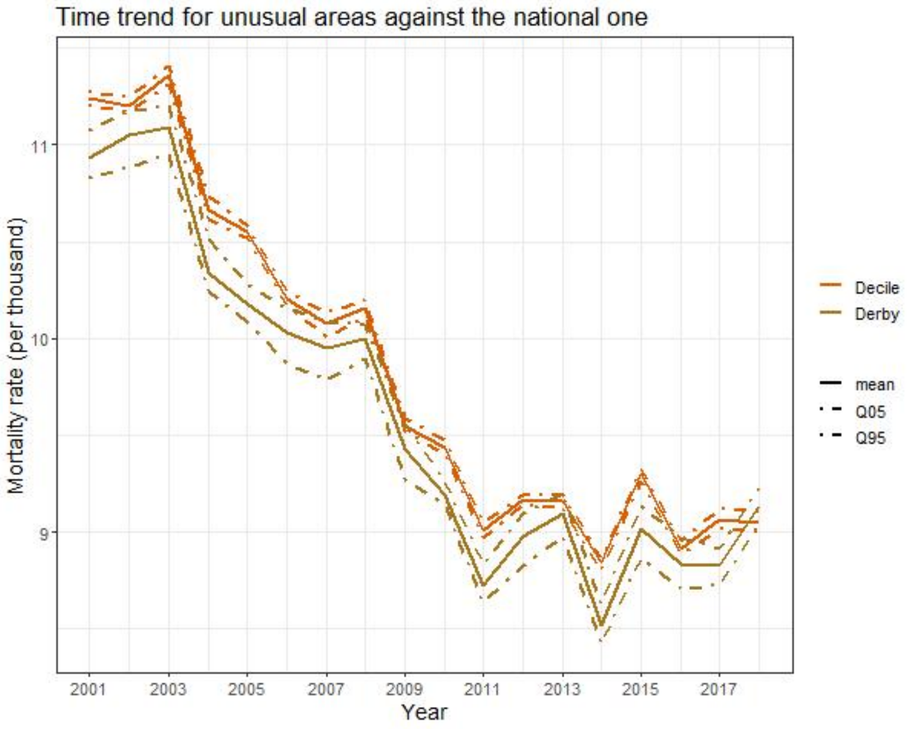

Figure S8: County Durham, females

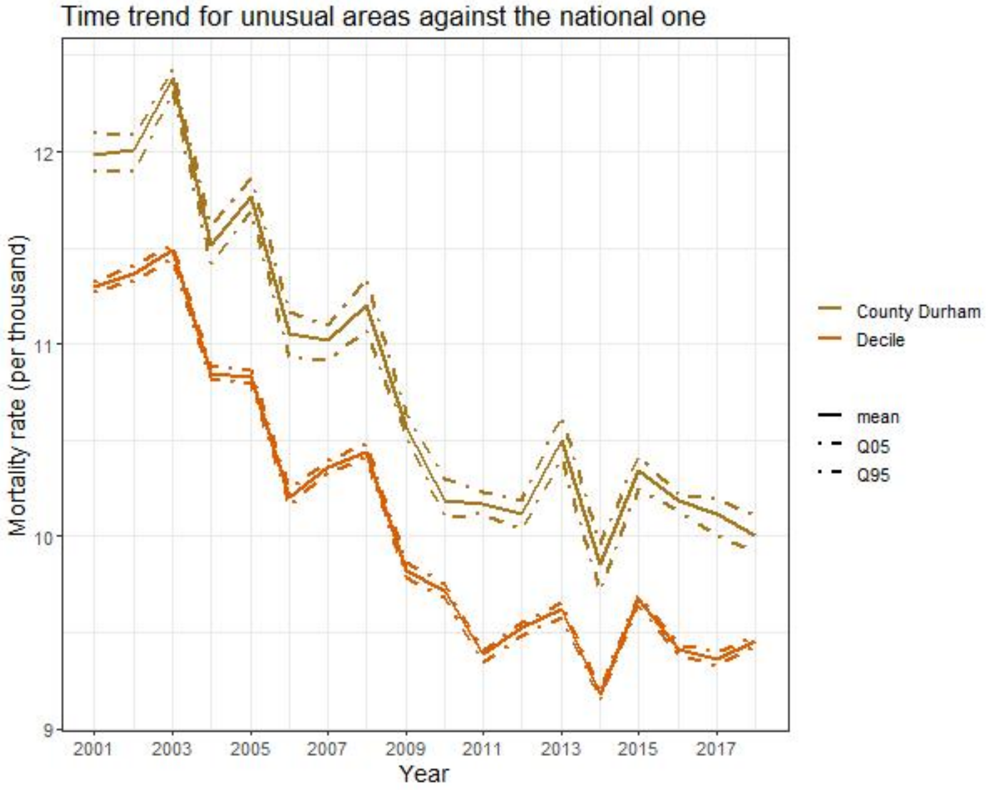

Figure S9: Cornwall including the Isles of Scilly, females

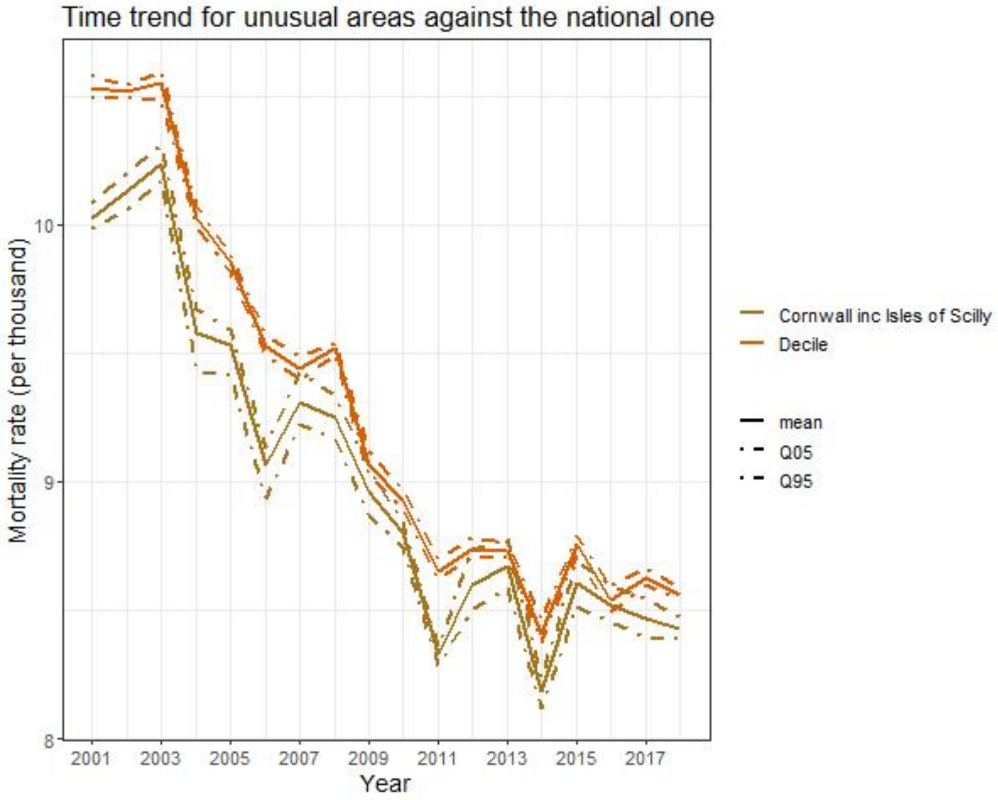

Figure S10: Bournemouth, Christchurch and Poole, Females

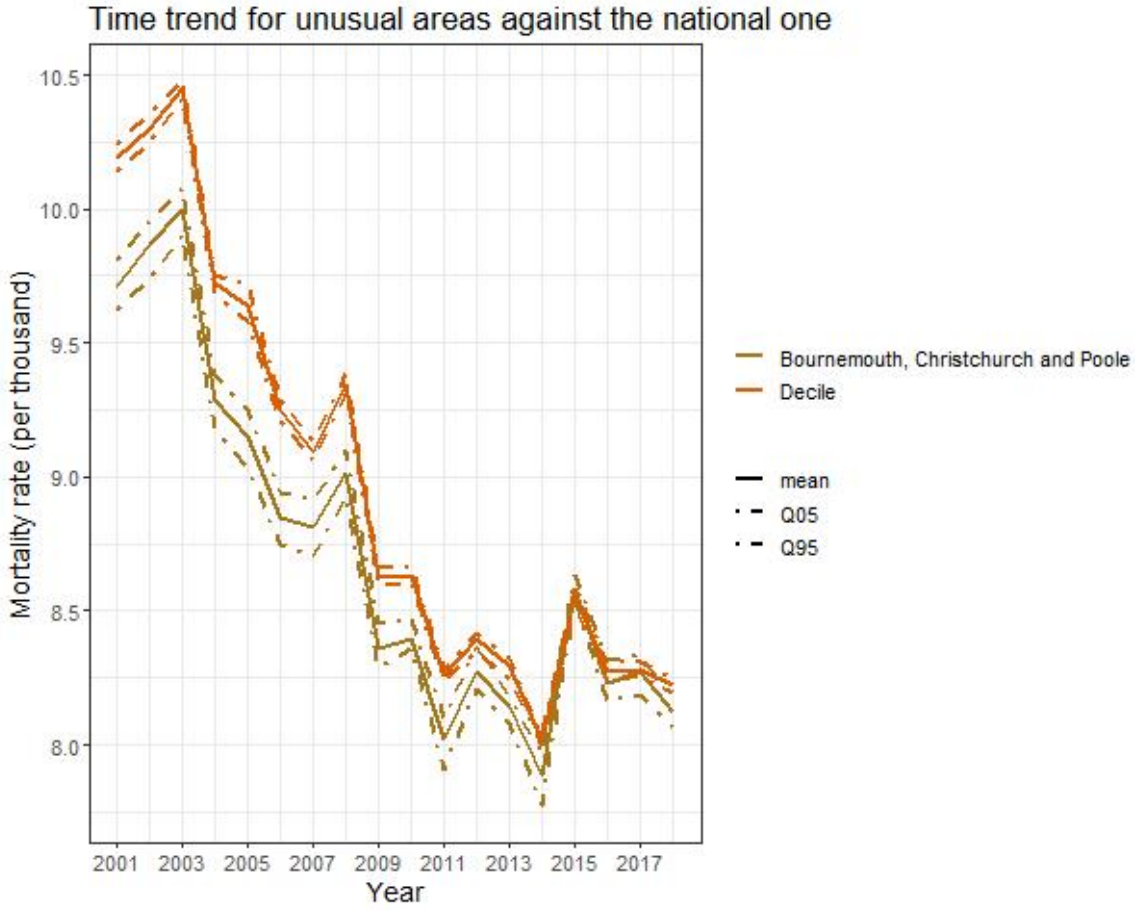

Figure S11: Stroud, females

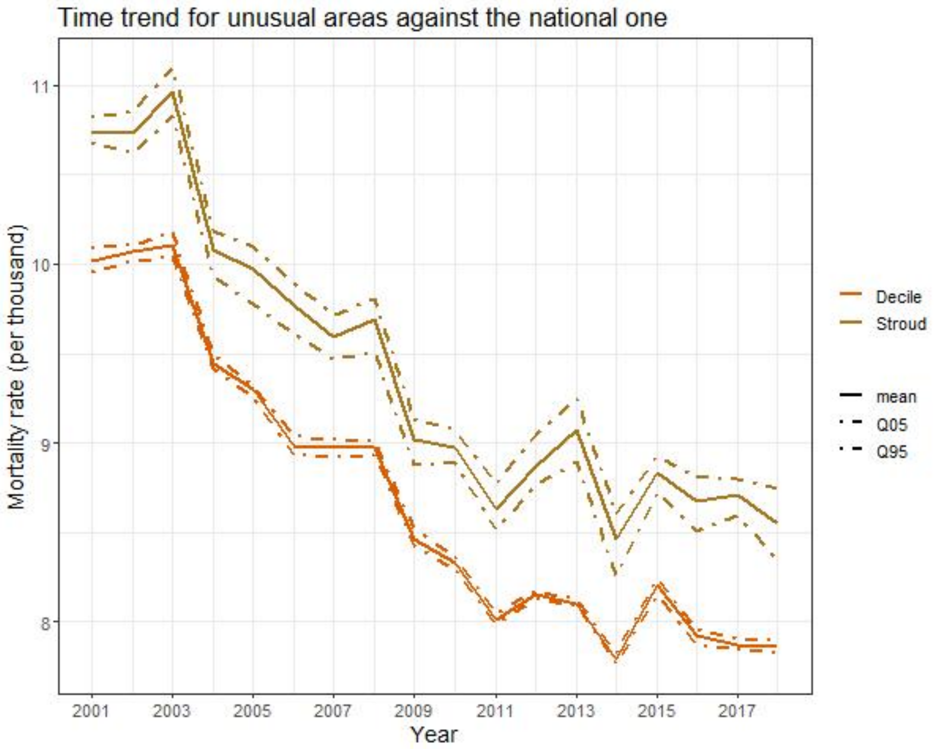

Figure S12: Sutton, females

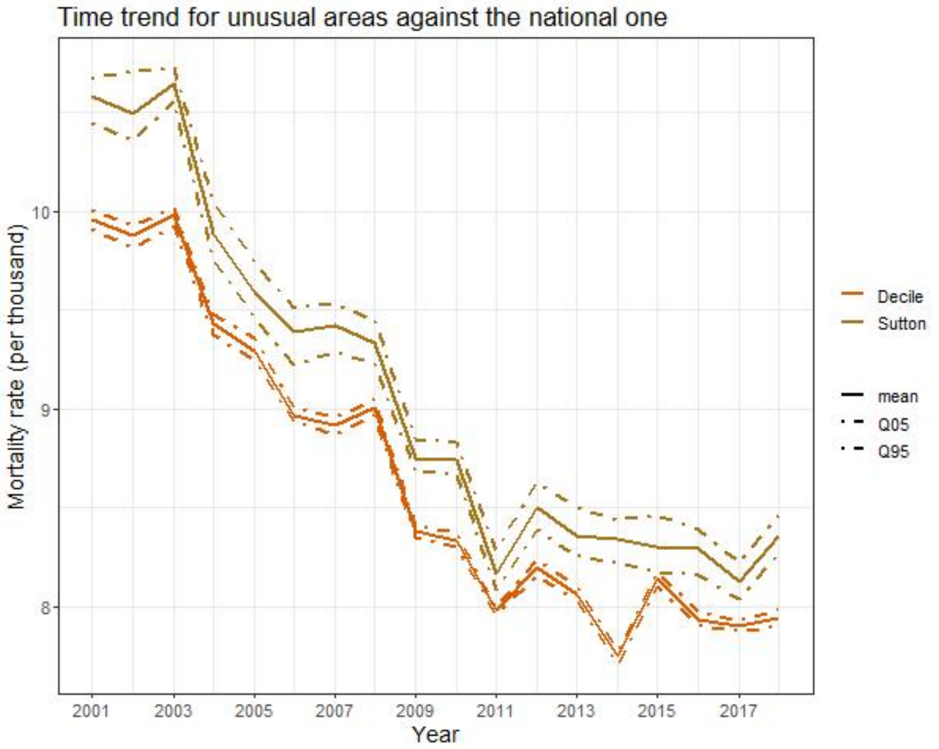

Mortality rates for the detected areas under the main model and comparison against the corresponding decile (IMD2019), males

Figure S13: Middlesbrough, males

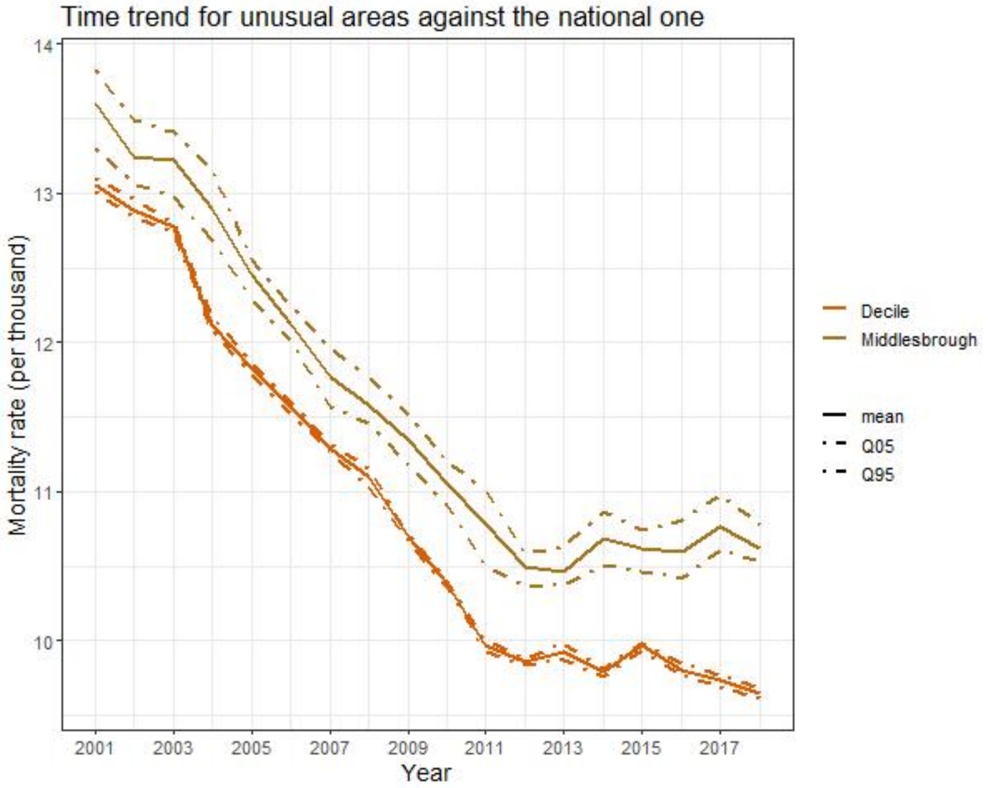

Figure S14: Wolverhampton, males

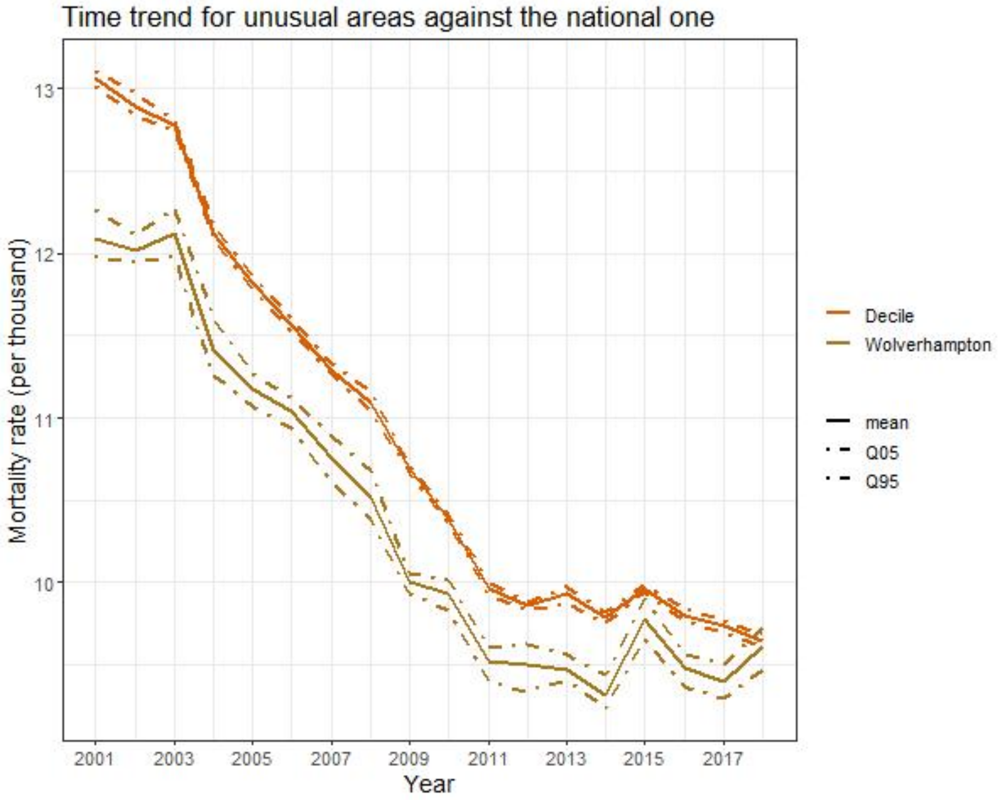

Figure S15: Kingston upon Hull, males

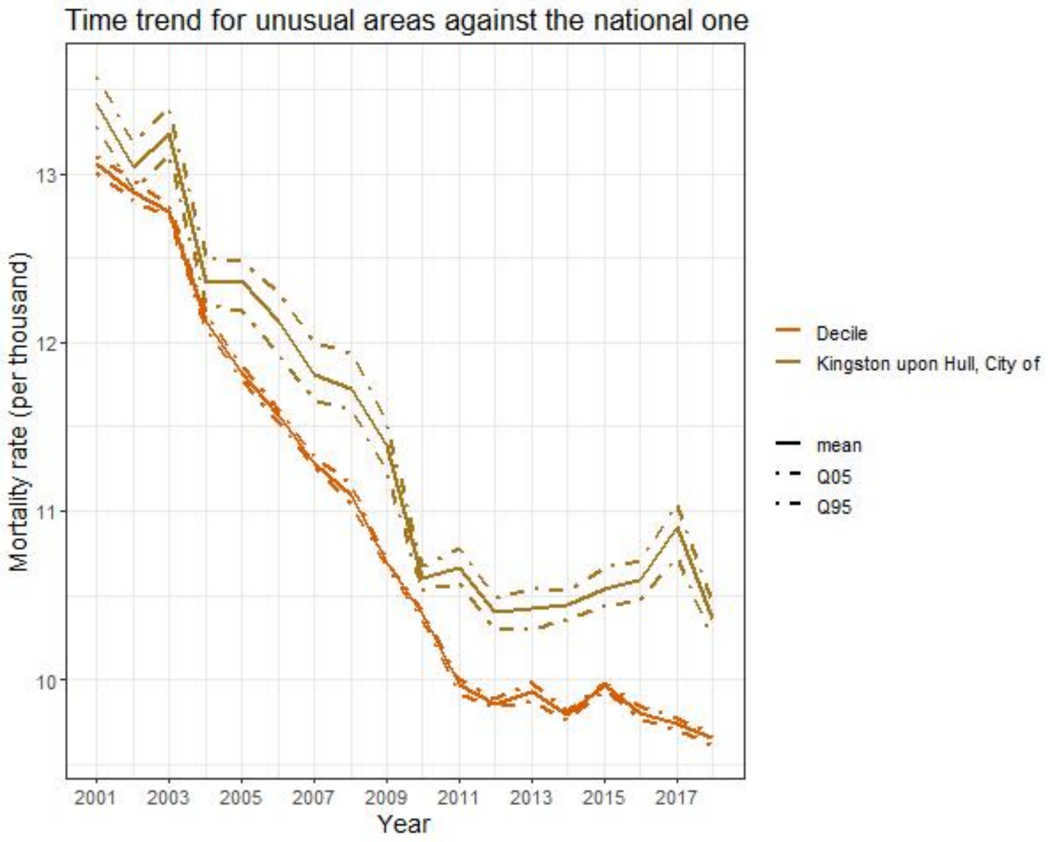

Figure S16: Birmingham, males

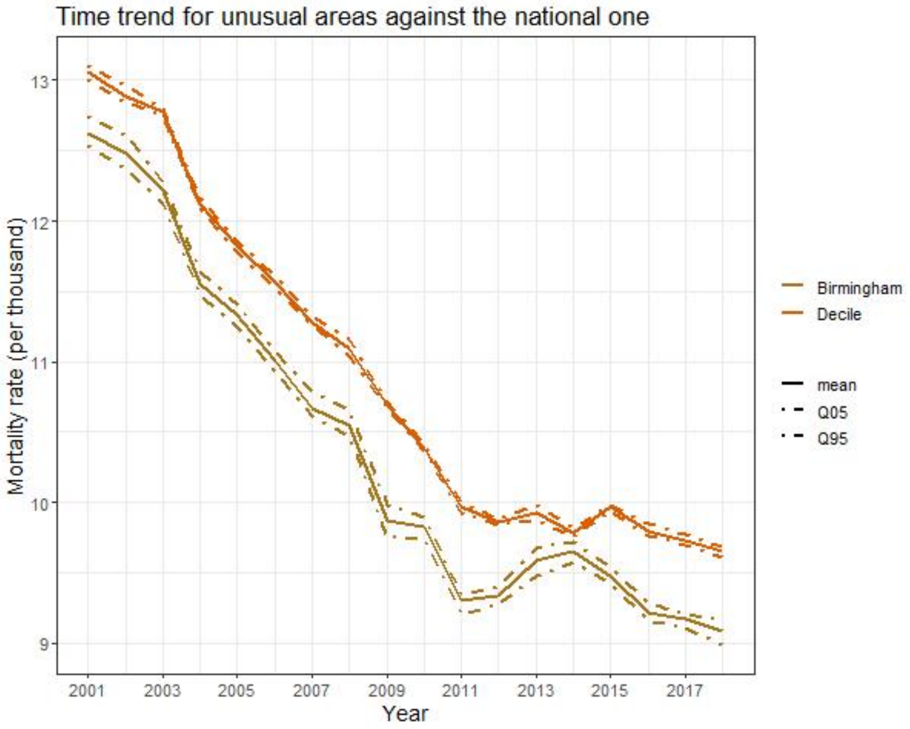

Figure S17: Tendring, males

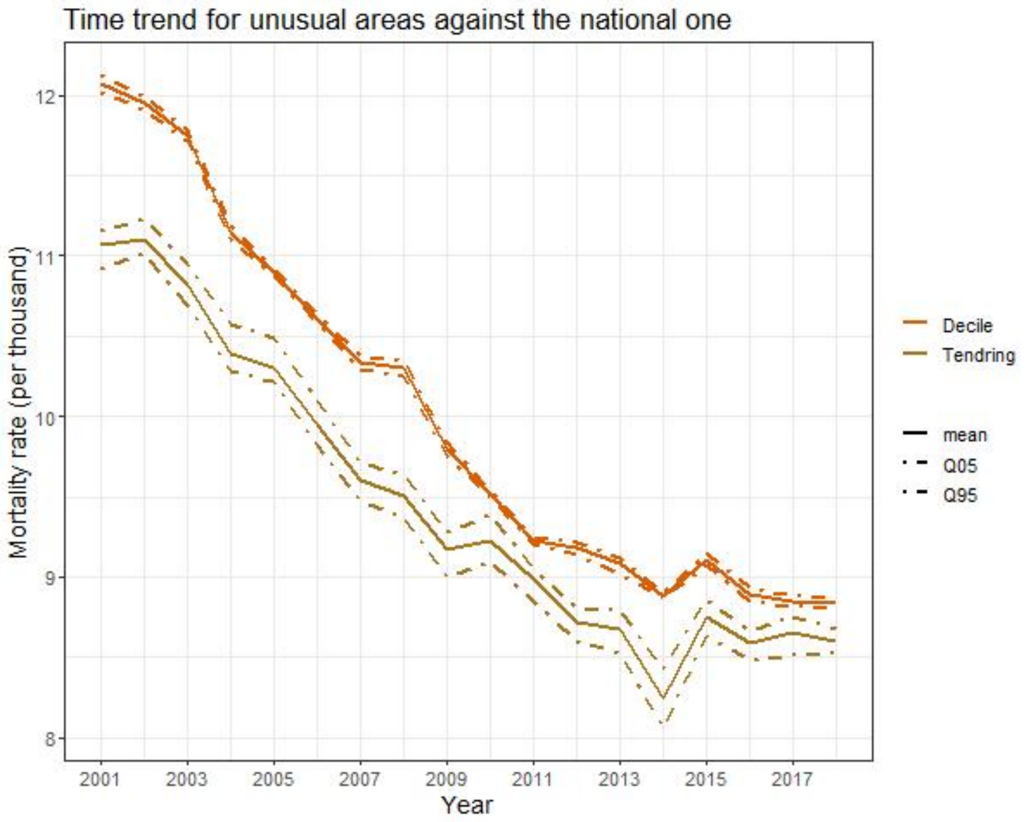

Figure S18: Leeds, males

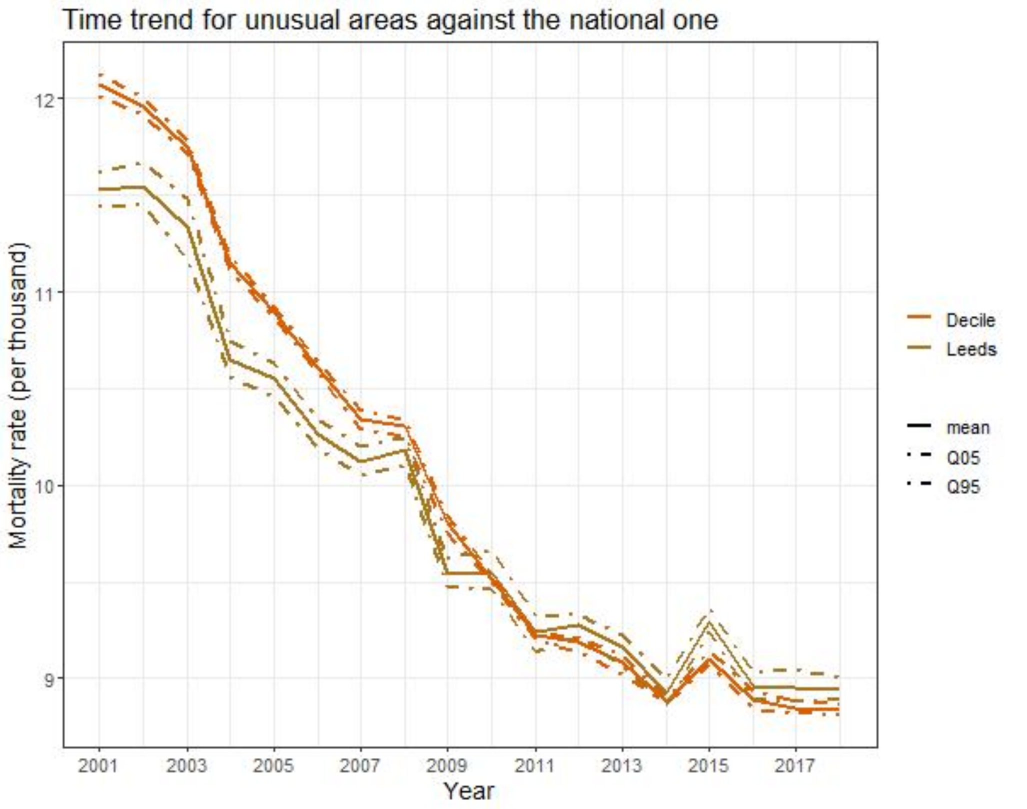

Figure S19: Newcastle upon Tyne, males

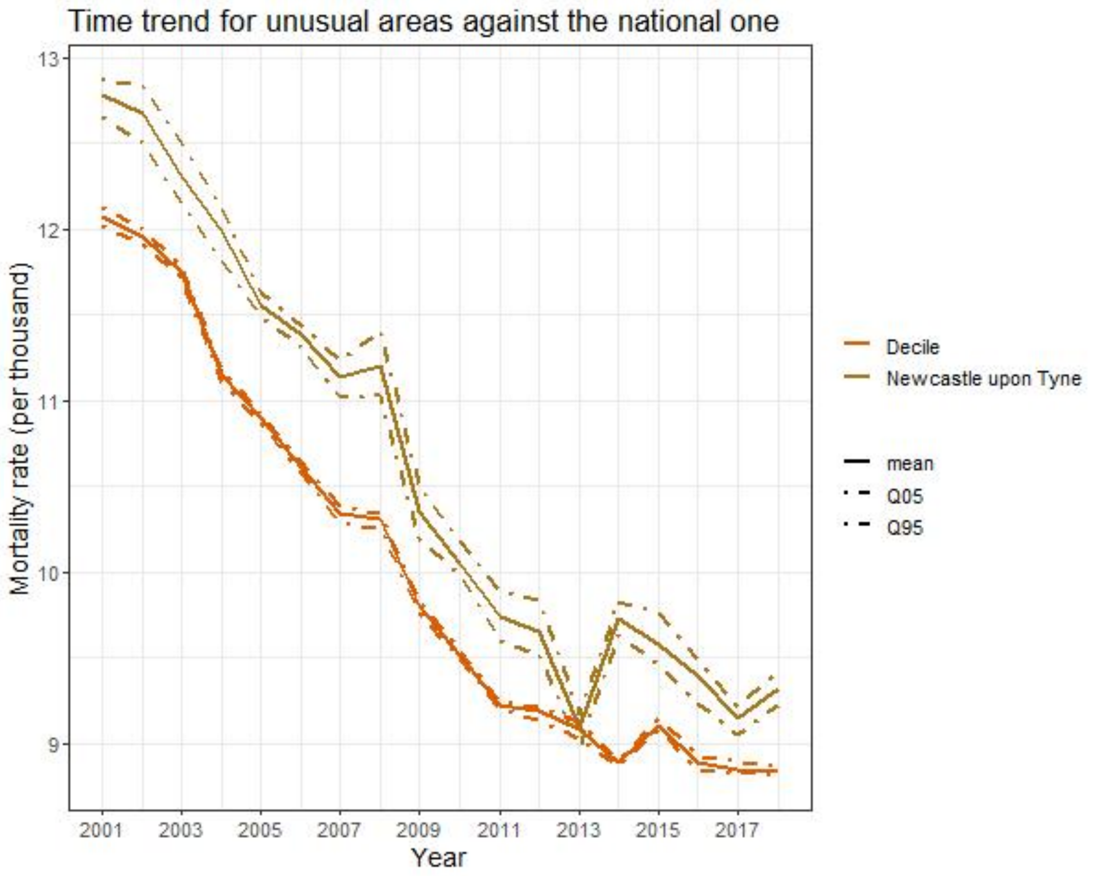

Figure S20: Southampton, males

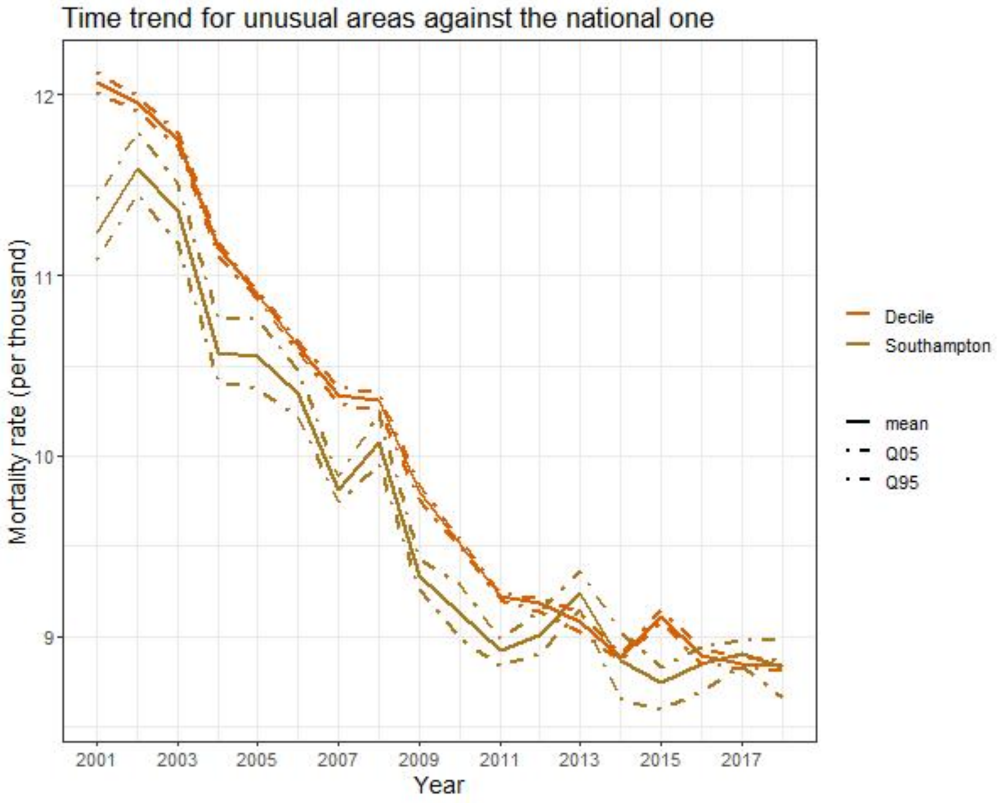

Figure S21: Bristol, males

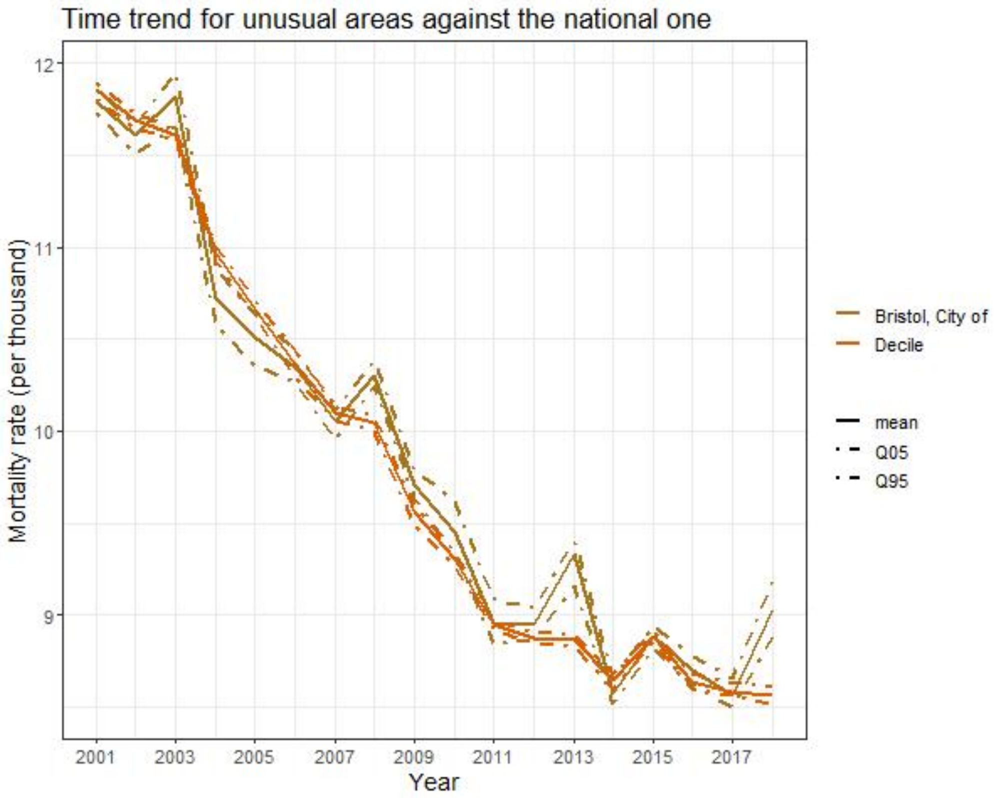

Figure S22: Cornwall including Isles of Scilly, males

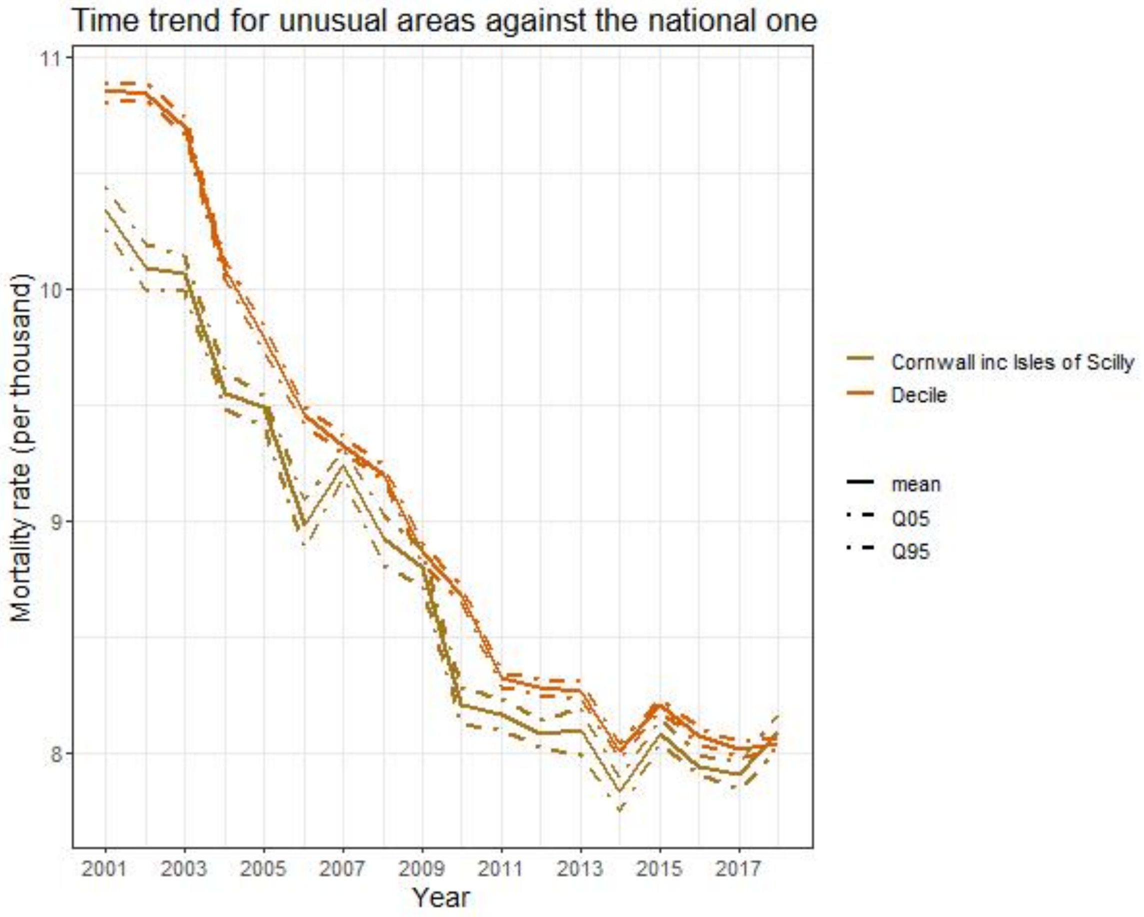

Figure S23: Breckland, males

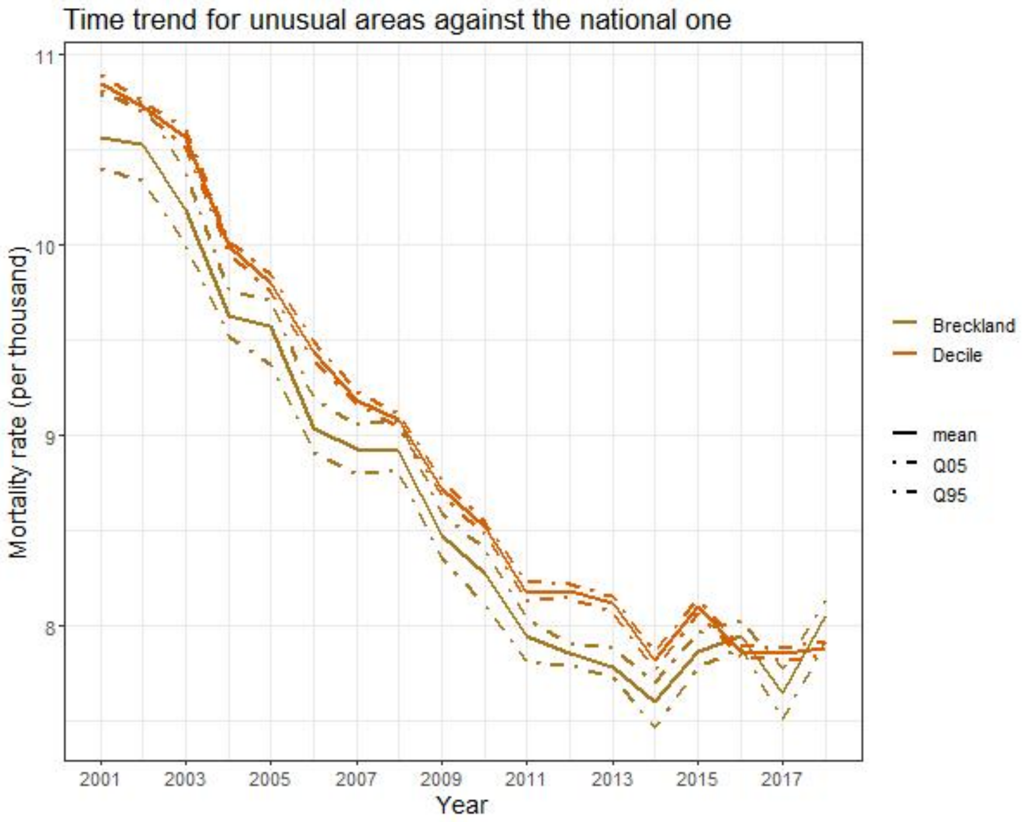

Figure S24: Ashford, males

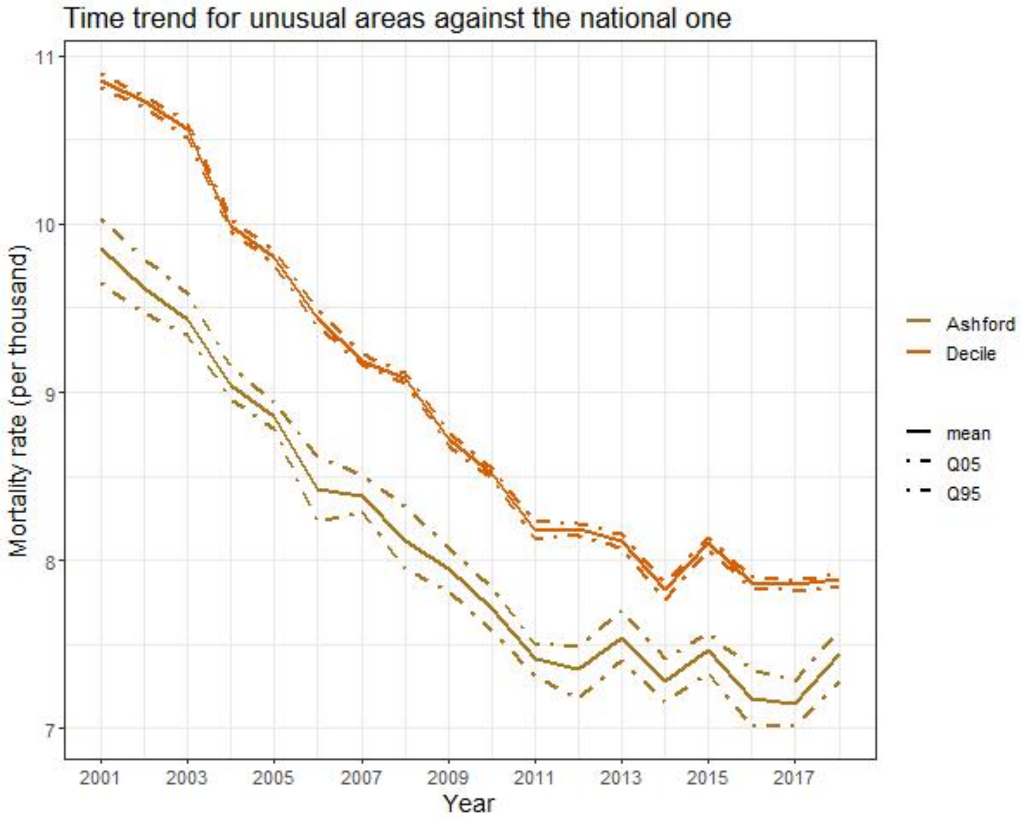

Figure S25: Hillingdon, males

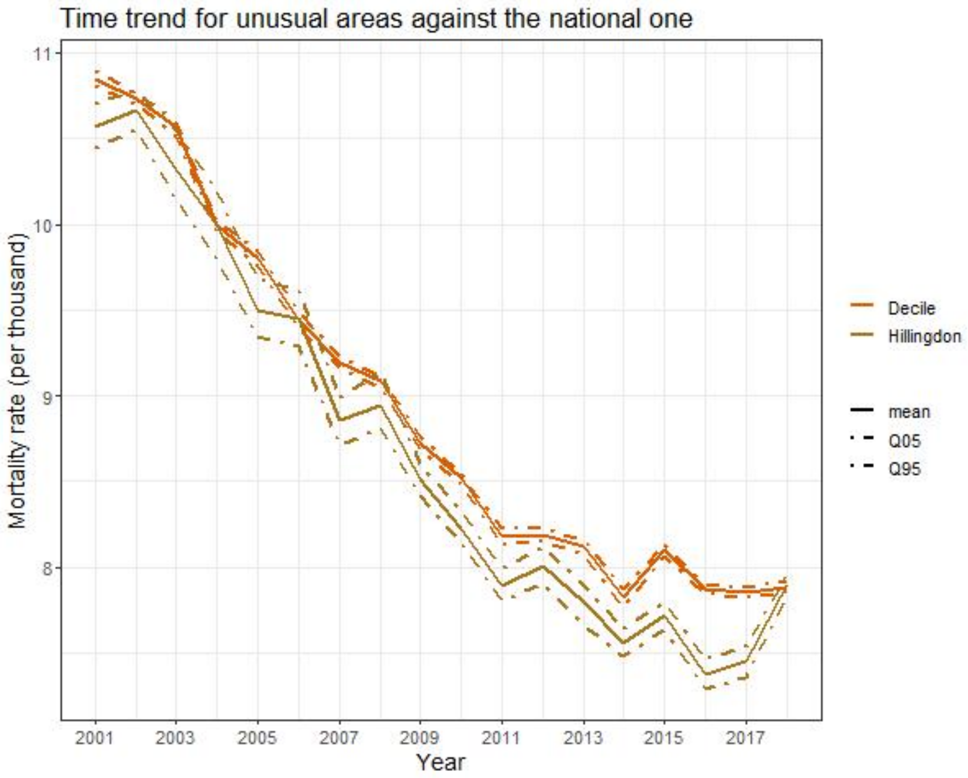

Figure S26: Cheshire West and Chester, males

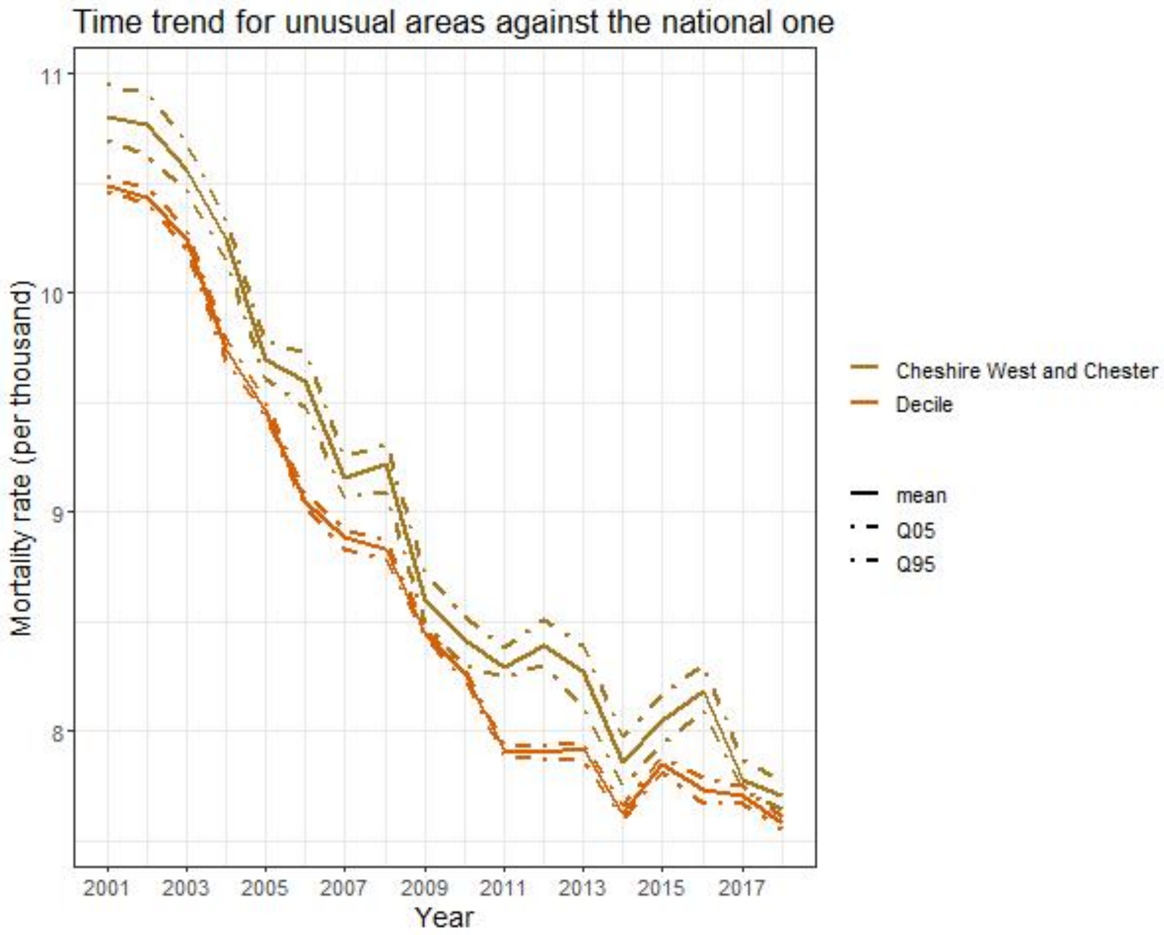

Figure S27: Dorset council, males

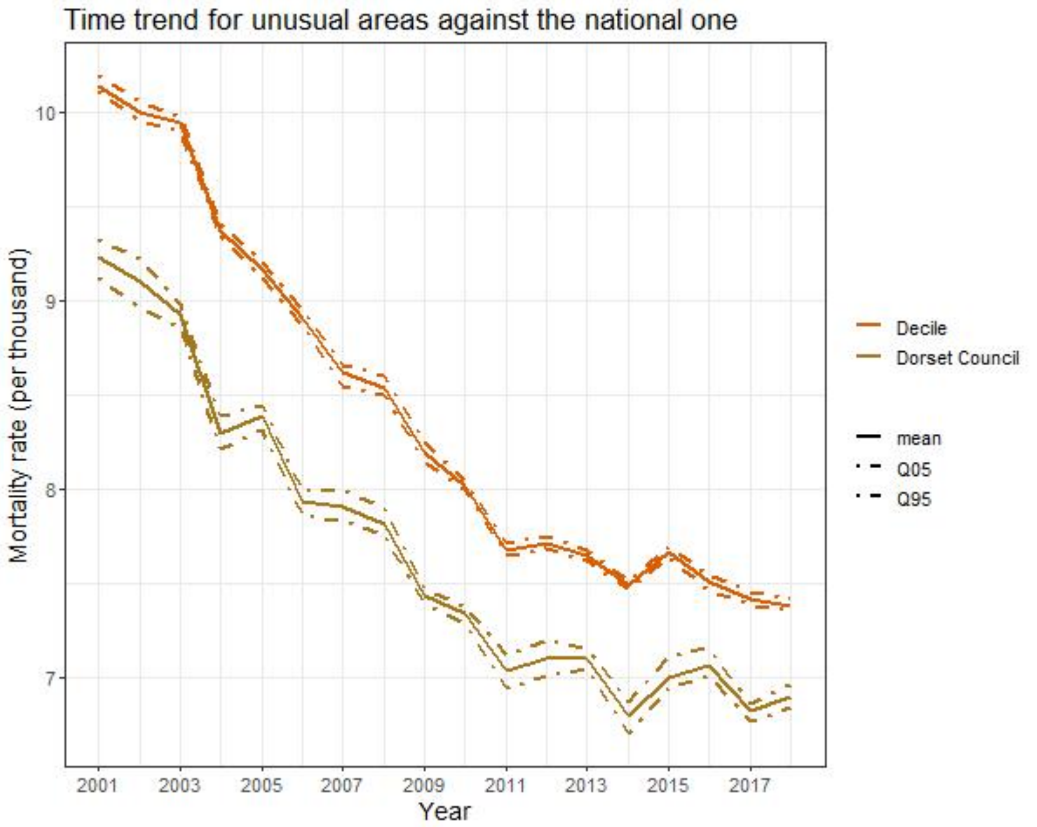

Figure S28: Teignbridge, males

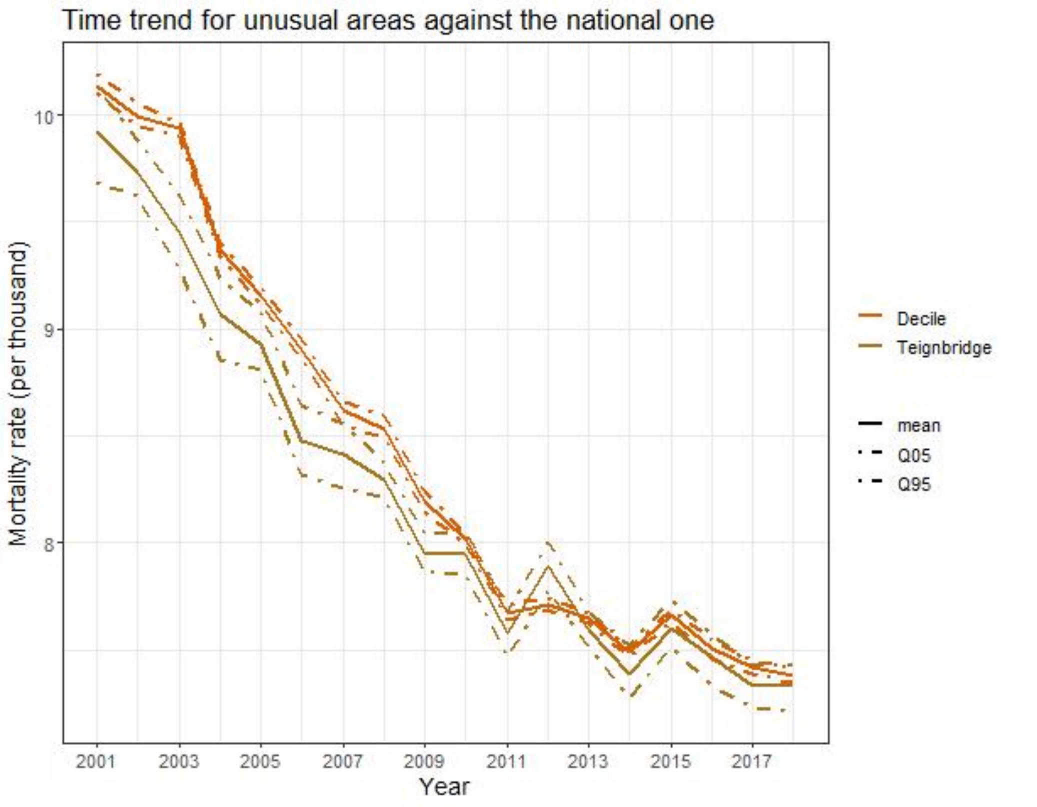

Figure S29: Stroud, males

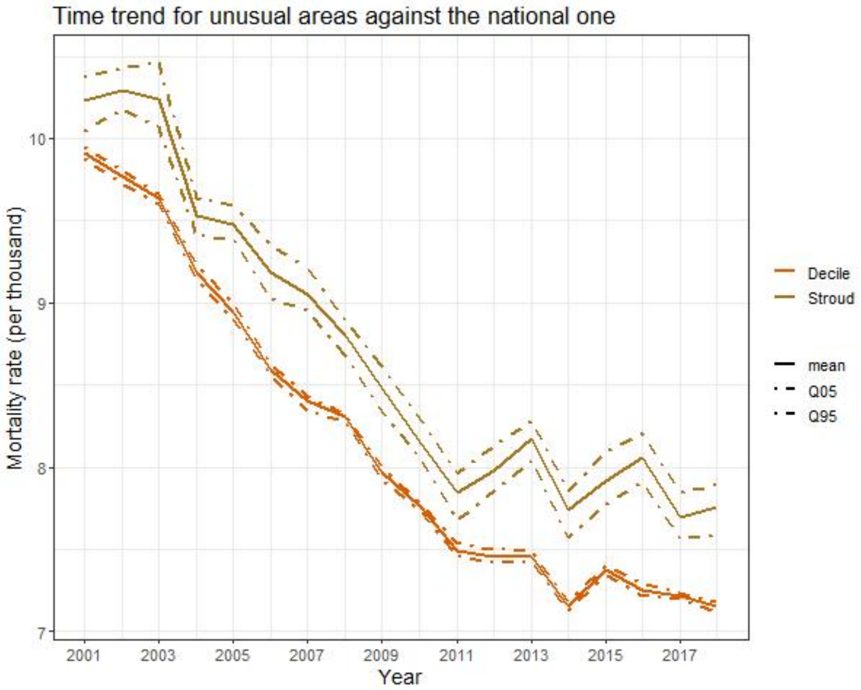

Figure S30: Bath and North East Somerset, males

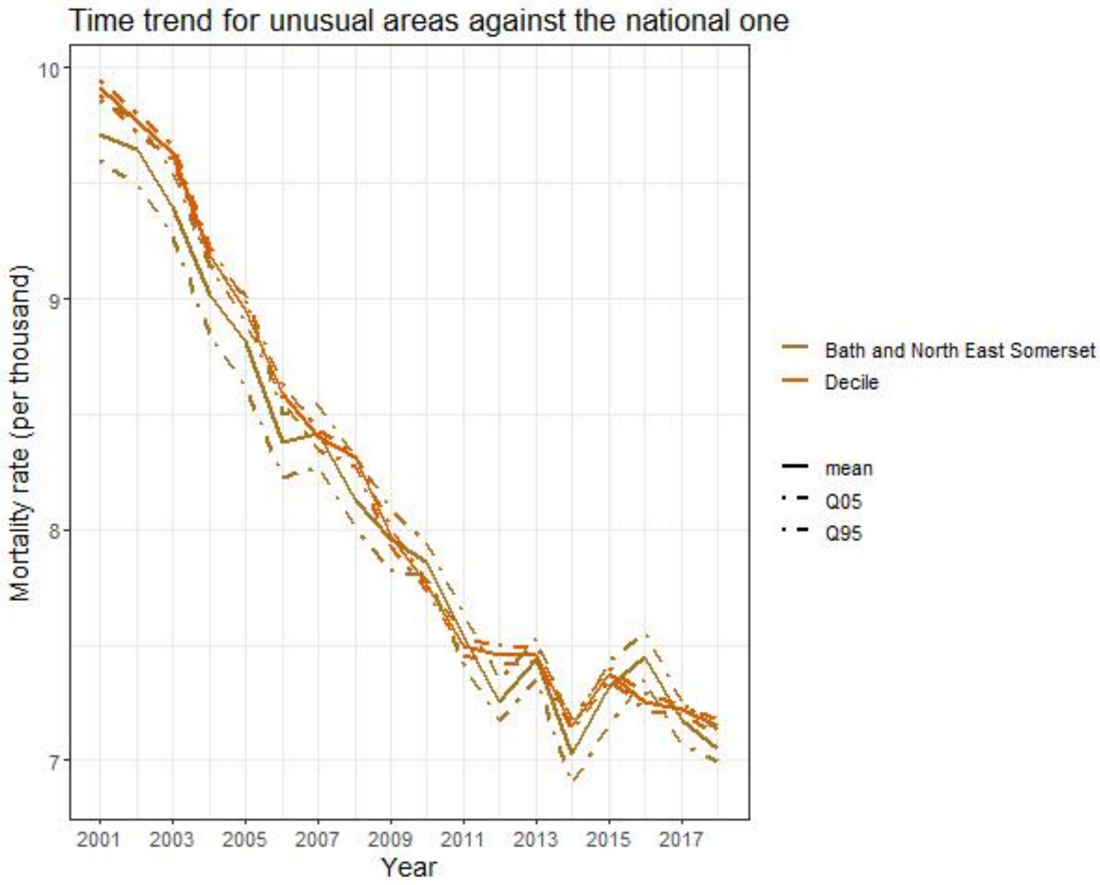

BMJ Open

A spatio-temporal model to estimate life expectancy and to detect unusual trends at the local authority level in England

Journal:	*BMJ Open*
Manuscript ID	bmjopen-2020-036855.R2
Article Type:	Original research
Date Submitted by the Author:	29-Sep-2020
Complete List of Authors:	Boulieri, Areti; Imperial College London School of Public Health, Department of Epidemiology and Biostatistics<br>Blangiardo, Marta; Imperial College London School of Public Health, Department of Epidemiology and Biostatistics
**Primary Subject Heading**:	Epidemiology
Secondary Subject Heading:	Public health
Keywords:	PUBLIC HEALTH, STATISTICS & RESEARCH METHODS, EPIDEMIOLOGY

SCHOLARONE™
Manuscripts

I, the Submitting Author has the right to grant and does grant on behalf of all authors of the Work (as defined in the below author licence), an exclusive licence and/or a non-exclusive licence for contributions from authors who are: i) UK Crown employees; ii) where BMJ has agreed a CC-BY licence shall apply, and/or iii) in accordance with the terms applicable for US Federal Government officers or employees acting as part of their official duties; on a worldwide, perpetual, irrevocable, royalty-free basis to BMJ Publishing Group Ltd ("BMJ") its licensees and where the relevant Journal is co-owned by BMJ to the co-owners of the Journal, to publish the Work in this journal and any other BMJ products and to exploit all rights, as set out in our licence.

The Submitting Author accepts and understands that any supply made under these terms is made by BMJ to the Submitting Author unless you are acting as an employee on behalf of your employer or a postgraduate student of an affiliated institution which is paying any applicable article publishing charge ("APC") for Open Access articles. Where the Submitting Author wishes to make the Work available on an Open Access basis (and intends to pay the relevant APC), the terms of reuse of such Open Access shall be governed by a Creative Commons licence – details of these licences and which Creative Commons licence will apply to this Work are set out in our licence referred to above.

Other than as permitted in any relevant BMJ Author's Self Archiving Policies, I confirm this Work has not been accepted for publication elsewhere, is not being considered for publication elsewhere and does not duplicate material already published. I confirm all authors consent to publication of this Work and authorise the granting of this licence.

For peer review only

A spatio-temporal model to estimate life expectancy and to detect unusual trends at the local authority level in England

Areti Boulieri[a], Marta Blangiardo[a*]

[a] Department of Epidemiology and Biostatistics, School of Public Health, Imperial College London, London, UK

[*] Corresponding author:
Marta Blangiardo
Imperial College London, St Mary's Campus,
Norfolk Place, W2 1PG
m.blangiardo@imperial.ac.uk

Keywords:
life expectancy, anomaly detection, area-level deprivation, spatio-temporal Bayesian model

Words: 3111

For peer review only

Abstract

Objectives

To estimate life expectancy at the local authority level and detect those areas that have a substantially low life expectancy after accounting for deprivation.

Design

We used registration data from the Office for National Statistics (ONS) on mortality and population in England, by local authority, age group, and socio-economic deprivation decile, for both males and females over the period 2001-2018. We used a statistical model within the Bayesian framework to produce robust mortality rates, which were then transformed to life expectancy estimates. A rule based on exceedance probabilities was used to detect local authorities characterised by a low life expectancy among areas with a similar deprivation level from 2012 onwards.

Results

We confirmed previous findings showing differences in the life expectancy gap between the most and least deprived areas from 2012 to 2018. We found variations in life expectancy trends across local authorities, and we detected a number of those with a low life expectancy when compared to others of a similar deprivation level.

Conclusions

There are factors other than deprivation that are responsible for low life expectancy in certain local authorities. Further investigation on the detected areas can help understand better the stalling of life expectancy which was observed from 2012 onwards and plan efficient public health policies.

Strengths and limitations of this study

- This is the first study of its scale, including more than 8 million records.
- The methodology produces robust estimates via the Bayesian hierarchical framework used.
- Through the detection of local authorities that deviate from the expected trend, our framework helps better understand the stalling of life expectancy in order to implement efficient public health measures.
- In the model, it was not possible to account for population changes due to migration.
- The socio-economic indicator used was based on data from the last available year; we performed a sensitivity analysis using the mid-year deprivation indicator for the period considered.

Introduction

The study of life expectancy is of primary interest in public health practice, where it is used to measure the overall health of a population, and also to plan for health and social service provision. Life expectancy has been improving steadily over the last decades, mainly due to changes in behavioural risk factors and healthcare. This phenomenon started to slow down from 2012 onwards and has become cause for concern in several country, including the USA and the UK.[1,2] Possible explanations of this include strikes in influenza among older people, potentially due to ineffective

For peer review only

vaccines,[3] or increases in mortality for mental health conditions, such as cognitive impairment.[4] Recent studies in the UK found that the excess number of deaths in 2015 was largely driven by the older populations and suggested potential associations with socioeconomic factors, poor health and social care, which can be linked to austerity measures across the health and social care system .[5,6,7]

Socio-economic deprivation has been found to be the major determinant of life expectancy by numerous studies over the years, regardless of the measure of deprivation that was used. [8, 9, 10, 11, 12, 13] Geographical patterns of life expectancy in England and Wales were found to be mainly attributable to variations in deprivation status in 1998.[8] A more recent study found that the elimination of socio-economic differences between areas would increase survival among older adults across a number of European countries, with the strongest association in England.[9] The impact of deprivation on overall levels of life expectancy is therefore established. However, the corresponding impact on life expectancy improvements over time is less clear. An early paper found that in most deprived areas improvements in life expectancy were negligible.[10] A more recent paper showed that the life expectancy gap between the most affluent and most deprived areas increased from 2001 to 2016 for both sexes, particularly for females.[11] This finding was not confirmed by later studies where largest improvements in life expectancy were documented in some of the most deprived areas.[14] Recent life expectancy trends by socio-economic deprivation have mostly been studied by aggregating areas in England into deciles of deprivation at area level.[11,15,16,17] Limited research has investigated the association between life expectancy and socio-economic deprivation at the smaller area level, including a study in Scotland[18] and one in Sweden.[19]

It is crucial to focus on small regions and detect those that are most in need. This would help better understand the life expectancy gap and support governments with evidence for public health prioritisation. This cannot be achieved with data description, as splitting the number of deaths by a combination of small area, age, gender and year leads to low number of cases and unstable mortality rates and life expectancy estimates. The need for a modelling approach has been highlighted to overcome this issue.[20] Additionally, we argue that it is important to 1) establish the role of socio-economic deprivation in detecting areas performing poorly in terms of life expectancy; and 2) detect those areas that differ from others with a similar level of socio-economic deprivation and show unexplained lower life expectancy. These regions should be investigated further to understand which factors, in addition to deprivation, are driving the stalling of life expectancy.

In this paper, we analysed all-cause mortality counts in England at the local authority level from 2001 to 2018 in order to assess the impact of deprivation on life expectancy improvements over time. Focusing on 2012-2018 when the stalling effect was observed, we identified areas which had lower life expectancy than other areas characterised by a similar level of deprivation.

Methods

Data

We used mortality counts from 2001 to 2018 at the local authority level in England. The local authority level is an administrative subdivision of England with population size ranging from 2.3 thousand (Isle of Scilly) to 1.6 million (Kent County Council). In total 315 local authorities were considered, after excluding the Isle of Scilly and the City of London due to their small population. Information on age and sex was available for each record. We considered 20 age groups, each including 5 years except for the first (< 1 year old) and the last group (90 and older) and we analysed males and females separately as these have been previously shown to have very different mortality levels and trends.[21] Population data by age and sex, as well as a measure of deprivation for each local authority were also used for the analysis. All data, originally held by the Office for National Statistics (ONS), were provided to us by Public Health England (PHE).

For peer review only

Deprivation data were available at the decile level. In this study we used the English index of multiple deprivation (IMD), based on seven different domains of deprivation: i) income deprivation, ii) employment deprivation, iii) education, skills and training deprivation, iv) health deprivation and disability, v) crime, vi) barriers to housing and services, and vii) living environment deprivation. Each local authority was allocated to a deprivation decile (D1, …, D10) with D1 being the most deprived and D10 the least deprived decile based on the IMD 2019.[22]

Patient and public involvement

As this was secondary research based on aggregated data that had already been collected, no patients or the public were involved in designing the study, nor were they involved in the analysis or implementation of the study. Dissemination to study participants or patient organisations was not applicable.

Statistical analysis

For peer review only

We developed a statistical model within a Bayesian hierarchical framework; we specified a Poisson distribution for the mortality counts (y_{ijtd}) by age group (*j=1,…,20*), local authority (*i=1,…,315*), calendar year (*t=1,…,18*), and deprivation decile (*d=1,…,10*):

$$y_{ijtd} \sim \text{Poisson}\,(\lambda_{ijtd} \times Pop_{ijt})$$

where Pop_{ijt} is the population and λ_{ijtd} the mortality rate. Borrowing from the disease mapping literature, the log transformed rate was then modelled as the linear combination of several terms as follows: [23,24]

$$\log(\lambda_{ijtd}) = a_0 + \alpha_j + \gamma_i + \zeta_t + \phi_d + \omega_{it} + \delta_{ij} + \kappa_{jt} + \nu_{dt}$$

where α_j, γ_i, ζ_t and ϕ_d are random effects identifying the main effects of age, local authority, year and deprivation deciles. Pairwise interactions between age and space (δ_{ij}), age and time (κ_{jt}), space and time (ω_{it}), and deprivation and time (ν_{dt}) were also included to allow for deviation from the main effects and to increase the level of flexibility of the model.

The Bayesian framework allows the incorporation of assumptions regarding the structure of the mortality rates via prior distributions on the parameters. It is known that mortality rates vary slowly with age, time, and to some extent across space and it is reasonable to expect that their trends have a smooth pattern. We therefore specified a random walk prior of order 1 (RW1) for the age-, time-, and deprivation-specific components α_j, ζ_t and ϕ_d, assuming that units that are consecutive in terms of age, time or deprivation decile have similar mortality rates. The spatial component γ_i was assigned a conditional autoregressive (CAR) prior, assuming that neighboring areas share similar characteristics in terms of mortality rates.[25] We assumed similarity across time and decile of deprivation for each age group, specifying a RW1 on the corresponding interaction terms, κ_{jt} and ν_{dt}. On the other hand, the interaction of local authority-age, and local authority- year were left unstructured and data driven, through an exchangeable Normal prior on δ_{ij} and ω_{it}. The overall intercept a_0 was assigned a vague Normal prior.

A rule to detect local authorities characterised by lower life expectancy was specified based on the space-time interaction term of the model ω_{it}, that, if above zero, would suggest a local increase of

mortality rates. We estimated the probability that the space-time interaction term was above zero for all space-time combinations and we selected the local authorities where this probability exceeded the threshold of 0.95. We restricted the detection on 2012-2018 to focus on the period characterised by the stalling of life expectancy. Further details on the model and the code for running it are available at https://github.com/aretib/LifeExpectancyDetect.

Results are presented as mean estimates and 95% credible intervals (CI). Mortality rates were transformed to life expectancy estimates for each local authority using life tables methods.[21] More details about the procedure to go from mortality rates to life expectancy are reported in https://github.com/aretib/LifeExpectancyDetect.

To evaluate the impact that a change in the definition of the deprivation index has on the detection, we present the results of the same model, where deprivation deciles are based on IMD 2010 (Supplementary Material).

Results

Life expectancy at birth in England was estimated to be 79.6 years for men and 83.2 years for women in 2018. Over the period 2001 to 2018, life expectancy followed an overall increasing trend, with a flatter pattern from 2012 onwards for both genders, and the gap between women and men decreasing over time. Figure 1 shows the time trends of life expectancy by deprivation decile group for men and women respectively. It is observed that the overall level of life expectancy is different across deciles, with higher deprivation corresponding to lower life expectancy. Additionally, it can be noted that life expectancy follows an overall increasing trend for all deciles. However, more deprived groups are characterised by flatter patterns compared to less deprived groups.

Focusing on 2012-2018, Figure 2 shows the gap in life expectancy between the beginning and the end of the period and their corresponding 95% interval estimates. There is a difference of more than 100 days in life expectancy gains for the least deprived decile compared to the most deprived decile for both sexes, while it is worth noting the large uncertainty across all the deciles (95% interval estimates depicted by the error bars). When comparing males and females, we observe bigger gains for males across most deprivation deciles. However there is an overlap in the uncertainties for males and females, in line with reports from ONS and PHE.[16,21]

We found variations in life expectancy trends across local authorities, and a number of those were detected as atypical (i.e. showing lower life expectancy than in other local authorities in the same deprivation decile) from 2012 to 2018. The maps in Figures 3 and 4 show the detected local authorities for males and females respectively with the corresponding number of times that these were picked up out of the 7 year period. In particular, 12 unique areas were detected for females and 18 areas for males (Tables 1 and 2). Looking at the maps, we do not observe any specific spatial pattern in the detected local authorities, as these are randomly scattered across England. From the tables it can be noted that roughly half of the detected local authorities belong to the two most deprived deciles (D1 and D2) for both sexes. Additionally, most local authorities were detected in 1 year only during the 7 year period, with Leeds standing out as it was detected in 2 years for females and 4 years for males. For each detected area, we show mortality rates temporal trends compared those of the corresponding deprivation decile in Supplementary Material (Figures S1-S30).

When running the sensitivity analysis considering the deprivation score from 2010 rather than 2019, we found that the detected areas increase to 31 for females and 25 for males; for both genders they include all the areas detected in the main model. We present the additional areas detected in Table S1 and S2 in Supplementary Material, where we include the posterior probability that ω_{it} is above 0 under the two models. For all the areas, these probabilities are similar and when an area is not detected under the model with IMD 2019, the posterior probability that ω_{it} is above 0 is not far from the 0.95 threshold, which suggests the results are robust. The 19 additional areas detected for

For peer review only

females ranges across deprivation deciles and 7 move from a less to a more deprived decile between 2010 and 2019; for males 5 additional areas belong to the most deprived deciles and the remaining 2 move from a less to a more deprived deciles between 2010 and 2019.

Discussion

In this analysis we investigated the life expectancy gap between the most and least deprived local authorities in England. We proposed a statistical tool to look at the life expectancy trends across local authorities and to detect those characterised by lower levels of life expectancy which should be further investigated in order to provide targeted policy measures.

Principal findings

Our analyses suggest that the increase in life expectancy in England from 2012 to 2018 has been greatest in areas with low levels of deprivation, with more modest increases in life expectancy in areas with high levels of deprivation.[13,21] More specifically, the gain in life expectancy in the wealthiest areas (decile 10) compared to the poorest areas (decile 1) in England was over 100 days for males, and roughly 80 days for females. The differences among deprivation deciles 2 to 9 were less clear and there was large uncertainty around the estimates.

We found that the life expectancy trends did not follow the same pattern across all local authorities for both males and females between 2012 and 2018. More specifically, we detected the local authorities which depicted a lower life expectancy compared to what would have been expected given their deprivation level. This suggests that factors other than deprivation are potentially responsible for the poor performance of the specific local authorities.

We note here that our study is descriptive as it does not formally assess potential causes in the detected areas. Nevertheless, we expect that it will inform the evidence around life expectancy gaps and the role of deprivation. This should be used as the basis for future studies targeting the detected local authorities to investigate potential responsible factors.

Follow up research should aim to disentangling the different domains of socio-economic deprivation, such as barriers to housing and services, or health deprivation and disability. This is needed to better identify if specific domains are responsible for the lower life expectancy observed in the detected local authorities. Methodologically, this requires the use of more complex statistical models, able to account for the potential correlation among the different domains.

Austerity has been linked to the stalling of life expectancy. [26,27] This should be further investigated, particularly looking at how different cities have implemented specific local policies related to austerity which might have contributed to the different life expectancy trends. These policies are likely to be targeting specific age groups (e.g. closure of youth centres or cut to the mental health services or pension credit) and a separate cause-specific analysis may help provide a better picture in this context.

Finally, any events or changes in a neighbourhood should be explored to assess any potential impact these might have on their life expectancy; as an example, life expectancy in an area could be lowered following the opening of a long-term care home , as older people from outside that area move in the facility.

Comparison to previous studies

Our life expectancy estimates are in line with the ONS estimates for both males and females. [21] In addition, we showed that deprivation has an impact not only on the overall life expectancy estimates but also on life expectancy improvements.[16] This is in line with an earlier study investigating the impact of a health inequalities intervention, implemented by the UK government between 1997 and

For peer review only

2010. The authors found that the life expectancy gap between the population living in the 20% most deprived local authorities and the rest of the population declined during the intervention, while this trend reversed after the intervention ended in 2010.[28]

In contrast to Bennett et al. who ran the analyses at the deprivation decile, the novelty of our study lies on the analysis of life expectancy at the local authority level, while accounting for deprivation.[13] We propose a mechanism to detect local authorities in England with lower life expectancy for their deprivation level and therefore provide a first step in the investigation and understanding of factors other than deprivation that might be responsible in order to develop targeted interventions.

Strengths and limitations

Our analysis of mortality registration data in England from 2001 to 2018 has a number of advantages. Firstly, to our knowledge, it was the first study of its scale, investigating life expectancy at the local authority level in the whole of England over an 18-year period, including more than 8 million records. Second, the methodology that we used, is appropriate for the small numbers that arise when mortality counts are considered by local authority, year, and age group and it produces robust estimates, unlike the standard statistical techniques frequently used for this type of analysis.[29] Third, we were able to detect specific local authorities in England which are most left behind in terms of life expectancy improvements, addressing an important gap in the literature. Finally, we proposed a statistical tool that can be used on a routine basis for detection of life expectancy anomalies in England while accounting for deprivation. A segment tool is produced by PHE to provide information on the life expectancy gap at the local authority level.[30] However, this is based on crude mortality rates which are less robust compared to the smoothed ones produced through statistical modelling. Additionally, it provides mean estimates over 2015-2017 rather than annual estimates, and it is not able to automatically flag areas which would require further investigation.

Limitations of our study include population changes because of migration, both within the country and overseas, which were not accounted for in the model. In addition, the allocation of local authorities into discrete groups of deprivation, inevitably yields some variation within local authorities, since not all people can be of the same socio-economic status, which is a common issue in ecological studies. Lastly, the socio-economic indicator that we used is based on data from a specific year and does not account for changes in the local authorities between deprivation deciles over the period under study. We chose the deprivation index at 2019 as it provided the most recent estimate of deprivation at small area. Additionally, as the data used to construct the index are related to the period 2013-2017, it is the best representation of deprivation in your detection period (2012-2018). Nevertheless, in our sensitivity analysis we were able to detect all the areas originally detected in the main model and we showed that the results are robust, when comparing the posterior probability that ω_{it} is above 0.

Conclusions

Our study has demonstrated a state of the art statistical tool for detecting specific local authorities in England that have low levels of life expectancy, while accounting for socio-economic deprivation. There is potential to implement this detection mechanism as part of a routine monitoring surveillance, in order to focus on specific areas that are most in need.

Footnotes

Contributors

Areti Boulieri and Marta Blangiardo equally contributed to the design of the study, statistical

For peer review only

analyses, interpretation and manuscript writing. The corresponding author attests that all listed authors meet authorship criteria and that no others meeting the criteria have been omitted.

Acknowledgements

The authors would like to thank Garyfallos Konstantinoudis, Monica Pirani and Chiara Forlani from Imperial College London and Allan Baker, Paul Fryers from PHE for their constructive comments which helped improve the paper.

Funding statement

Areti Boulieri acknowledges support from an MRC Early Career Research Fellowship (MR/M501669/1). Marta Blangiardo acknowledges support by Public Health England award titled "Assessment of Trends in Life Expectancy in England and its Upper-tier Local authorities". Infrastructure support for the Department of Epidemiology and Biostatistics was provided by the NIHR Imperial Biomedical Research Centre (BRC). This work was part supported by the MRC Centre for Environment and Health, which is currently funded by the Medical Research Council (MR/S019669/1).

Competing interests

None.

Ethical approval

Not required.

Data sharing

The data used for the study were provided by Public Health England (PHE) and cannot be shared online due to confidentiality.

Transparency declaration

The lead author (AB) affirms that the manuscript is an honest, accurate, and transparent account of the study being reported; that no important aspects of the study have been omitted; and that any discrepancies from the study as planned have been explained.

For peer review only

328 **Tables**

329

Local authority	Decile	Year
Kingston upon Hull, City of	D1	2017
Stoke-on-Trent	D1	2018
Tameside	D1	2018
Newcastle upon Tyne	D2	2015
Plymouth	D2	2017
Leeds	D2	2015, 2018
Derby	D3	2018
County Durham	D4	2013
Cornwall inc Isles of Scilly	D4	2016
Bournemouth, Christchurch and Poole	D6	2015, 2017
Stroud	D9	2013
Sutton	D8	2014

330

331 *Table 1: Local authorities with a low life expectancy for their deprivation level for females*

332

333

For peer review only

Local authority	Decile	Year
Middlesbrough	D1	2017
Wolverhampton	D1	2018
Kingston upon Hull, City of	D1	2017
Birmingham	D1	2014
Tendring	D2	2017
Leeds	D2	2012, 2015, 2017, 2018
Newcastle upon Tyne	D2	2014
Southampton	D2	2013
Bristol, City of	D3	2013, 2018
Cornwall inc Isles of Scilly	D4	2018
Breckland	D5	2018
Ashford	D5	2018
Hillingdon	D5	2018
Cheshire West and Chester	D6	2016
Dorset Council	D7	2016
Teignbridge	D7	2012
Stroud	D9	2016
Bath and North East Somerset	D9	2016

Table 2: Local authorities with a low life expectancy for their deprivation level for males

For peer review only

Figure legends

Figure 1: Life expectancy estimates from 2001 to 2018 across deprivation deciles; the shaded areas correspond to 95% CI's

Figure 2: Life expectancy gap between 2012-2013 and 2017-2018; the error bars correspond to 95% CI's

Figure 3: Local authorities detected with a lower life expectancy than others with a similar deprivation level for females

Figure 4: Local authorities detected with a lower life expectancy than others with a similar deprivation level for males

For peer review only

References

[1] Olshansky, S Jay, Douglas J Passaro, Ronald C Hershow, Jennifer Layden, Bruce A Carnes, Jacob Brody, Leonard Hayflick, Robert N Butler, David B Allison, and David S Ludwig. 2005. "A Potential Decline in Life Expectancy in the United States in the 21st Century." *New England Journal of Medicine* 352 (11): 1138–45.

[2] Hiam, Lucinda, Dominic Harrison, Martin McKee, and Danny Dorling. 2018. "Why Is Life Expectancy in England and Wales 'Stalling'?" *Journal of Epidemiology and Community Health* 72 (5): 404–8.

[3] Pebody, RG, HK Green, F Warburton, M Sinnathamby, J Ellis, K Mølbak, J Nielsen, S de Lusignan, and N Andrews. 2018. "Significant Spike in Excess Mortality in England in Winter 2014/15–Influenza the Likely Culprit." *Epidemiology & Infection*, 1–8.

[4] Liddell, Christine, Chris Morris, Barbara Gray, Anna Czerwinska, and Ben Thomas. 2016. "Excess Winter Mortality Associated with Alzheimer's Disease and Related Dementias in the Uk: A Case for Energy Justice." *Energy Research & Social Science* 11: 256–62.

[5] Hiam, Lucinda, Danny Dorling, Dominic Harrison, and Martin McKee. 2017a. "What Caused the Spike in Mortality in England and Wales in January 2015?" *Journal of the Royal Society of Medicine* 110 (4): 131–37.

[6] Hiam, Lucinda, Danny Dorling, Dominic Harrison, and Martin McKee. 2017b. "Why Has Mortality in England and Wales Been Increasing? An Iterative Demographic Analysis." *Journal of the Royal Society of Medicine* 110 (4): 153–62.

[7] Watkins, Johnathan, Wahyu Wulaningsih, Charlie Da Zhou, Dominic C Marshall, Guia DC Sylianteng, Phyllis G Dela Rosa, Viveka A Miguel, Rosalind Raine, Lawrence P King, and Mahiben Maruthappu. 2017. "Effects of Health and Social Care Spending Constraints on Mortality in England: A Time Trend Analysis." *BMJ Open* 7 (11): e017722.

[8] Woods, Laura M, Bernard Rachet, Michael Riga, Noell Stone, Anjali Shah, and Michel P Coleman. 2005. "Geographical Variation in Life Expectancy at Birth in England and Wales Is Largely Explained by Deprivation." *Journal of Epidemiology & Community Health* 59 (2): 115–20.

[9] Ribeiro, Ana Isabel, Elias Teixeira Krainski, Marilia Sá Carvalho, Guy Launoy, Carole Pornet, and Maria de Fátima de Pina. 2018. "Does Community Deprivation Determine Longevity After the Age of 75? A Cross-National Analysis." *International Journal of Public Health* 63 (4): 469–79.

[10] Raleigh, Veena Soni, and Victor A Kiri. 1997. "Life Expectancy in England: Variations and Trends by Gender, Health Authority, and Level of Deprivation." *Journal of Epidemiology & Community Health* 51 (6): 649–58.

[11] Bennett, James E, Jonathan Pearson-Stuttard, Vasilis Kontis, Simon Capewell, Ingrid Wolfe, and Majid Ezzati. 2018. "Contributions of Diseases and Injuries to Widening Life Expectancy Inequalities in England from 2001 to 2016: A Population-Based Analysis of Vital Registration Data." *The Lancet Public Health* 3 (12): e586–e597.

[12] Kim, J.I. and Kim, G. 2018. "Effects on inequality in life expectancy from a social ecology perspective." *BMC public health*, *18*(1):243.

[13] Kim, J.I. and Kim, G. 2014. Factors affecting the survival probability of becoming a centenarian for those aged 70, based on the human mortality database: income, health expenditure, telephone, and sanitation. *BMC geriatrics*, *14*(1):113.

[14] Public Health England. 2019a. "Recent Trends in Mortality in England: Review and Data

For peer review only

Packs. A Report on Recent Trends in Life Expectancy and Mortality in England." https://www.gov.uk/government/publications/recent-trends-in-mortality-in-england-review-and-data-packs.

[15] Mayhew, Les, Gillian Harper, and Andrés M Villegas. 2020. "An Investigation into the Impact of Deprivation on Demographic Inequalities in Adults." *Annals of Actuarial Science*, doi: 10.1017/S1748499520000068.

[16] Public Health England. 2018. "Health Profile for England: 2018." https://www.gov.uk/government/publications/health-profile-for-england-2018.

[17] Iacobucci, Gareth. 2019. "Life Expectancy Gap Between Rich and Poor in England Widens." *British Medical Journal*, 364:l1492.

[18] Seaman, Rosie, Tim Riffe, Alastair H Leyland, Frank Popham, and Alyson van Raalte. 2019. "The Increasing Lifespan Variation Gradient by Area-Level Deprivation: A Decomposition Analysis of Scotland 1981–2011." *Social Science & Medicine* 230: 147–57.

[19] Åström, Daniel Oudin, Paul W Franks, and Kristina Sundquist. 2018. "Neighborhoods and Mortality in Sweden: Is Deprivation Best Assessed Nationally or Regionally?" *Demographic Research* 38: 429–50.

[20] Newton, John N. 2018. "Dissecting the Life Expectancy Gap in England Provides Clues on How to Reduce It." *The Lancet Public Health* 3 (12): e560–e561.

[21] Office for National Statistics. 2019a. "National Life Tables, UK: 2016 to 2018." https://www.ons.gov.uk/releases/nationallifetablesuk2016to2018.

[22] Office for National Statistics. 2019b. "Data: National Life Tables, England." https://www.ons.gov.uk/peoplepopulationandcommunity/birthsdeathsandmarriages/lifeexpectancies/datasets/nationallifetablesenglandreferencetables.

[23] Lawson A, Lee D. 2017 Bayesian disease mapping for public health. In: *Handbook of statistics*. Elsevier. 443–81.

[24] Best N, Richardson S, Thomson A. 2005. A comparison of Bayesian spatial models for disease mapping. *Statistical Methods in Medical Research*; 14:35–59.

[25] Besag J, York J, Mollié A. 1991. Bayesian image restoration, with two applications in spatial statistics. *Annals of the Institute of Statistical Mathematics*; 43:1–20.

[26] Christie, Bryan. 2019. "Study Cites Austerity as Factor in Stalling of Life Expectancy in Rich Countries." *British Medical Journal* 364:l645.

[27] Loopstra, Rachel, Martin McKee, Srinivasa Vittal Katikireddi, David Taylor-Robinson, Ben Barr, and David Stuckler.2016. "Austerity and old-age mortality in England: a longitudinal cross-local area analysis, 2007–2013." *Journal of the Royal Society of Medicine* 109 (3): 109–116.

[28] Barr, Ben, James Higgerson, and Margaret Whitehead. 2017. "Investigating the Impact of the English Health Inequalities Strategy: Time Trend Analysis." *British Medical Journal* 358: j3310.

[29] White, Chris. 2015. "Trend in Life Expectancy at Birth and at Age 65 by Socio-Economic Position Based on the National Statistics Socio-Economic Classification, England and Wales: 1982-1986 to 2007-2011." *Statistical Bulletin*.

[30] Public Health England. 2019b. "Segment Tool: 2019 Data Update."https://www.gov.uk/government/statistics/segment-tool-2019-data-update.

For peer review only

For peer review only

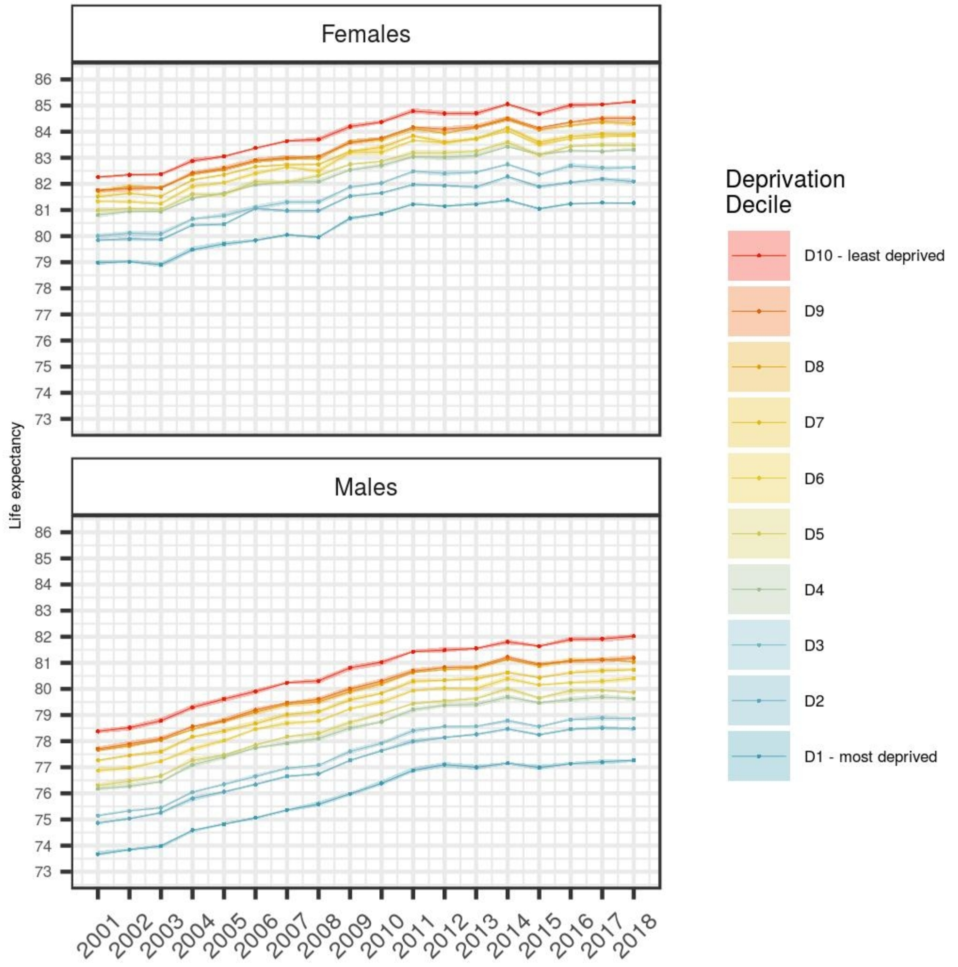

Figure 1: Life expectancy estimates from 2001 to 2018 across deprivation deciles; the shaded areas correspond to 95% CI's

89x89mm (300 x 300 DPI)

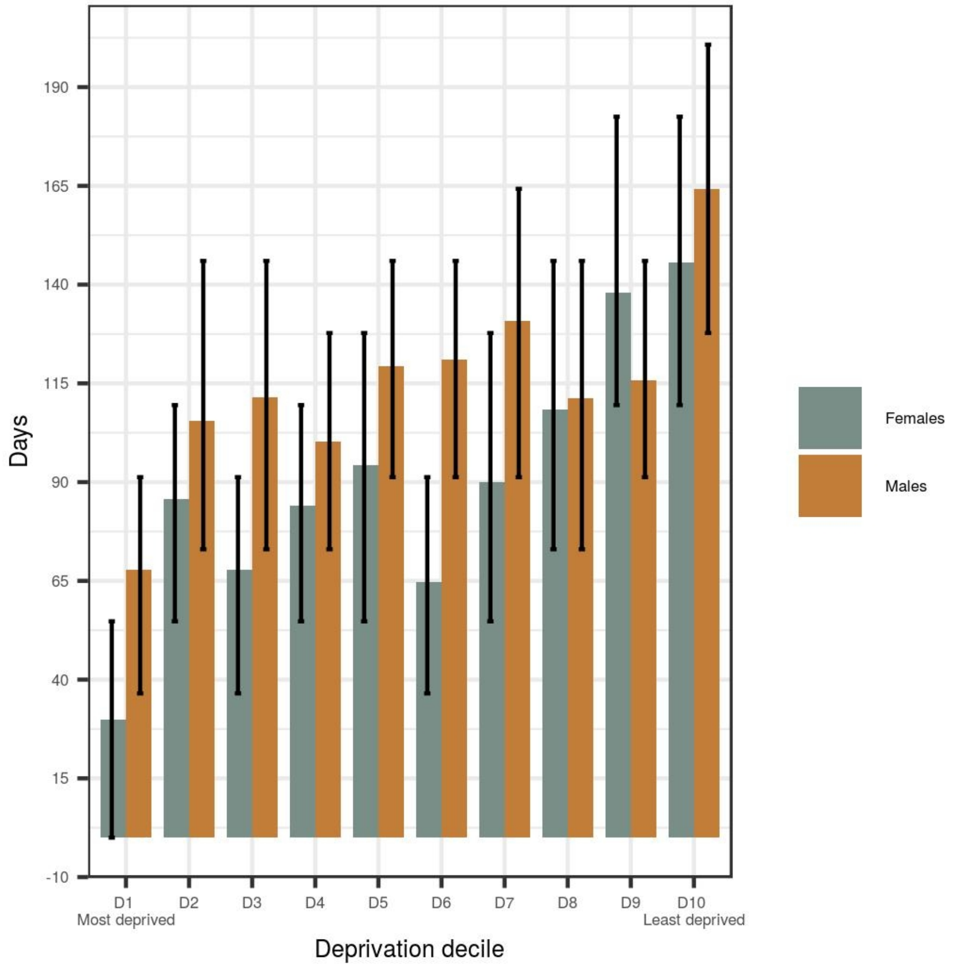

Figure 2: Life expectancy gap between 2012-2013 and 2017-2018; the error bars correspond to 95% CI's

89x89mm (300 x 300 DPI)

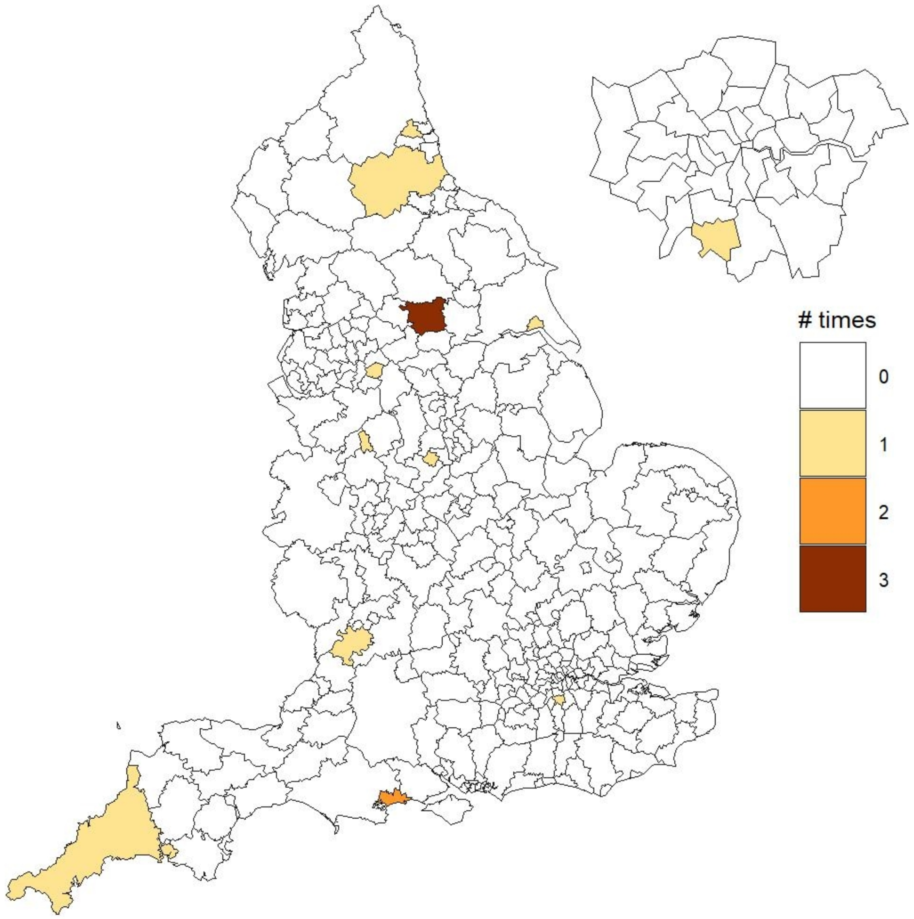

Caption : Figure 3: Local authorities detected with a lower life expectancy than others with a similar deprivation level for females

89x89mm (300 x 300 DPI)

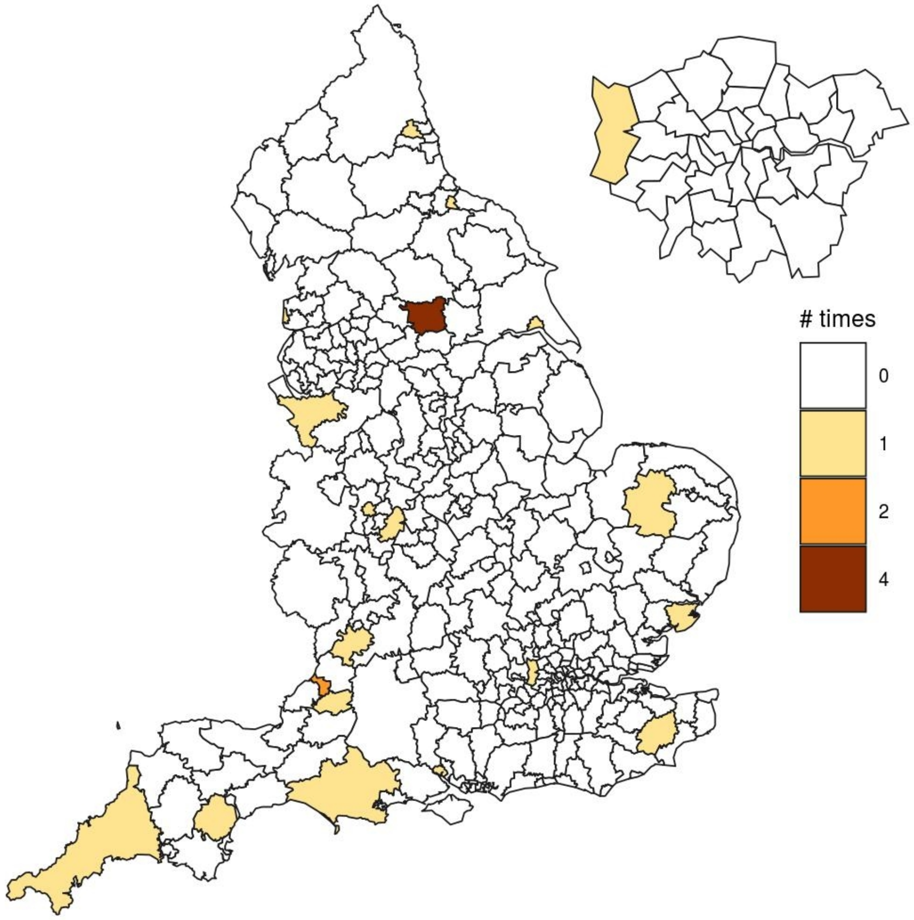

Figure 4: Local authorities detected with a lower life expectancy than others with a similar deprivation level for males

89x89mm (300 x 300 DPI)

Supplementary Material: A spatio-temporal model to estimate life expectancy and to detect unusual trends at the local authority level in England

Areti Boulieri[a], Marta Blangiardo[a*]

[a] Department of Epidemiology and Biostatistics, MRC Centre for Environment and Health, Imperial College London, London, UK

[*] Corresponding author:
Marta Blangiardo
Imperial College London, St Mary's Campus,
Norfolk Place, W2 1PG
m.blangiardo@imperial.ac.uk

For peer review only

Table S1: *: Local authorities with a low life expectancy for their deprivation level for females under the model with deprivation index at 2010. The areas detected under the model with deprivation index at 2019 are a subset (in bold). We present the posterior probability under the two models.*

		IMD2010		IMD2019	
Local Authority	Year	Decile	Pr(ω_{it}>0.95)	Decile	Pr(ω_{it}>0.95)
Blackpool	2017	D1	0.976	D1	0.912
Blackpool	2018	D1	0.959	D1	0.871
Bradford	2013	D1	0.981	D1	0.941
Kingston upon Hull, City of	2014	D1	0.951	D1	0.881
Kingston upon Hull, City of	2015	D1	0.972	D1	0.914
**Kingston upon Hull, City of**	**2017**	**D1**	**0.997**	**D1**	**0.977**
Kingston upon Hull, City of	2018	D1	0.981	D1	0.915
Manchester	2014	D1	0.960	D1	0.894
Oldham	2014	D1	0.980	D1	0.949
Stoke-on-Trent	2017	D1	0.980	D1	0.913
**Stoke-on-Trent**	**2018**	**D1**	**1.000**	**D1**	**0.999**
**Tameside**	**2018**	**D1**	**0.993**	**D1**	**0.969**
Wolverhampton	2017	D1	0.961	D1	0.864
Wolverhampton	2018	D1	0.991	D1	0.941
**Leeds**	**2013**	**D3**	**0.999**	**D2**	**0.997**
**Leeds**	**2015**	**D3**	**0.981**	**D2**	**0.958**
**Leeds**	**2018**	**D3**	**1.000**	**D2**	**1.000**
**Newcastle upon Tyne**	**2015**	**D2**	**0.974**	**D2**	**0.951**
Mansfield	2017	D2	0.962	D2	0.919
**Plymouth**	**2017**	**D3**	**0.986**	**D2**	**0.970**
King's Lynn and West Norfolk	2017	D3	0.951	D3	0.922
Medway	2014	D3	0.952	D3	0.930
**Derby**	**2018**	**D4**	**0.954**	**D3**	**0.956**
**County Durham**	**2013**	**D3**	**0.989**	**D4**	**0.978**
Cornwall inc Isles of Scilly	2013	D3	0.956	D4	0.923
**Cornwall inc Isles of Scilly**	**2016**	**D3**	**0.974**	**D4**	**0.964**
North Tyneside	2017	D4	0.954	D4	0.925
Breckland	2015	D5	0.956	D5	0.912
Stockport	2013	D5	0.967	D5	0.942
**Bournemouth, Christchurch and Poole**	**2015**	**D3**	**0.998**	**D6**	**0.996**
Bournemouth, Christchurch and Poole	2016	D3	0.959	D6	0.941
**Bournemouth, Christchurch and Poole**	**2017**	**D3**	**0.975**	**D6**	**0.963**
Colchester	2014	D7	0.959	D6	0.925
Colchester	2017	D7	0.970	D6	0.939
East Riding of Yorkshire	2012	D7	0.967	D7	0.931

For peer review only

Epping Forest	2014	D7	0.955	D7	0.905
Dorset Council	2016	D7	0.982	D7	0.946
North Somerset	2015	D7	0.967	D7	0.920
Chelmsford	2017	D6	0.965	D8	0.908
**Sutton**	**2014**	**D6**	**0.974**	**D8**	**0.954**
Tonbridge and Malling	2018	D8	0.954	D8	0.889
**Stroud**	**2013**	**D8**	**0.977**	**D9**	**0.954**
Stroud	2017	D8	0.952	D9	0.932
Harrogate	2014	D9	0.951	D9	0.901

For peer review only

Table S2*: Local authorities with a low life expectancy for their deprivation level for males under the model with deprivation index at 2010. The areas detected under the model with deprivation index at 2019 are a subset (in bold). We present the posterior probability under the two models.*

		IMD2010		IMD2019	
Local Authority	Year	Decile	Pr(ω_{it}>0.95)	Decile	Pr(ω_{it}>0.95)
Middlesbrough	2014	D1	0.962	D1	0.944
**Middlesbrough**	**2017**	**D1**	**0.989**	**D1**	**0.976**
**Blackpool**	**2015**	**D1**	**0.993**	**D1**	**0.985**
Blackpool	2018	D1	0.952	D1	0.896
Kingston upon Hull, City of	2016	D1	0.955	D1	0.892
**Kingston upon Hull, City of**	**2017**	**D1**	**1.000**	**D1**	**0.998**
Leicester	2015	D1	0.953	D1	0.902
Leicester	2017	D1	0.960	D1	0.891
Manchester	2014	D1	0.970	D1	0.933
Stoke-on-Trent	2018	D1	0.974	D1	0.918
South Tyneside	2018	D2	0.967	D1	0.945
**Birmingham**	**2014**	**D1**	**1.000**	**D1**	**0.996**
Walsall	2015	D2	0.970	D1	0.933
Walsall	2017	D2	0.974	D1	0.920
**Wolverhampton**	**2018**	**D1**	**0.995**	**D1**	**0.978**
Bradford	2016	D1	0.965	D1	0.870
**Leeds**	**2015**	**D3**	**0.980**	**D2**	**0.985**
**Newcastle upon Tyne**	**2014**	**D2**	**0.967**	**D2**	**0.970**
**Southampton**	**2013**	**D3**	**0.974**	**D2**	**0.977**
**Tendring**	**2017**	**D3**	**0.961**	**D2**	**0.967**
**Bristol, City of**	**2013**	**D3**	**0.975**	**D3**	**0.985**
**Bristol, City of**	**2018**	**D3**	**0.977**	**D3**	**0.990**
**Cornwall inc Isles of Scilly**	**2018**	**D3**	**0.995**	**D4**	**0.993**
**Ashford**	**2018**	**D6**	**0.957**	**D5**	**0.958**
**Breckland**	**2018**	**D6**	**0.995**	**D5**	**0.995**
**Hillingdon**	**2018**	**D4**	**0.951**	**D5**	**0.959**
Cherwell	2014	D8	0.952	D7	0.942
**Dorset Council**	**2016**	**D7**	**0.963**	**D7**	**0.965**
**Teignbridge**	**2012**	**D6**	**0.965**	**D7**	**0.964**
**Stroud**	**2016**	**D8**	**0.975**	**D9**	**0.976**
**Bath and North East Somerset**	**2016**	**D9**	**0.966**	**D9**	**0.967**

Mortality rates for the detected areas under the main model and comparison against the corresponding decile (IMD2019), females

Figure S1: Kingston upon Hull, females

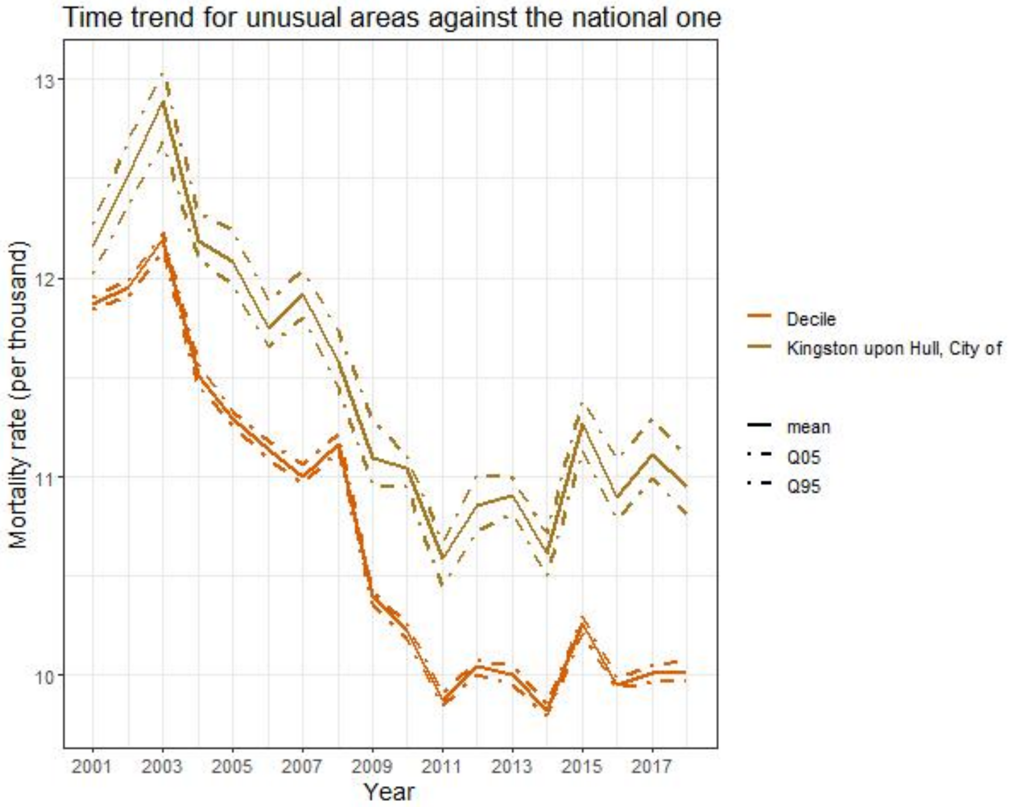

Figure S2: Stoke-on-Trent, females

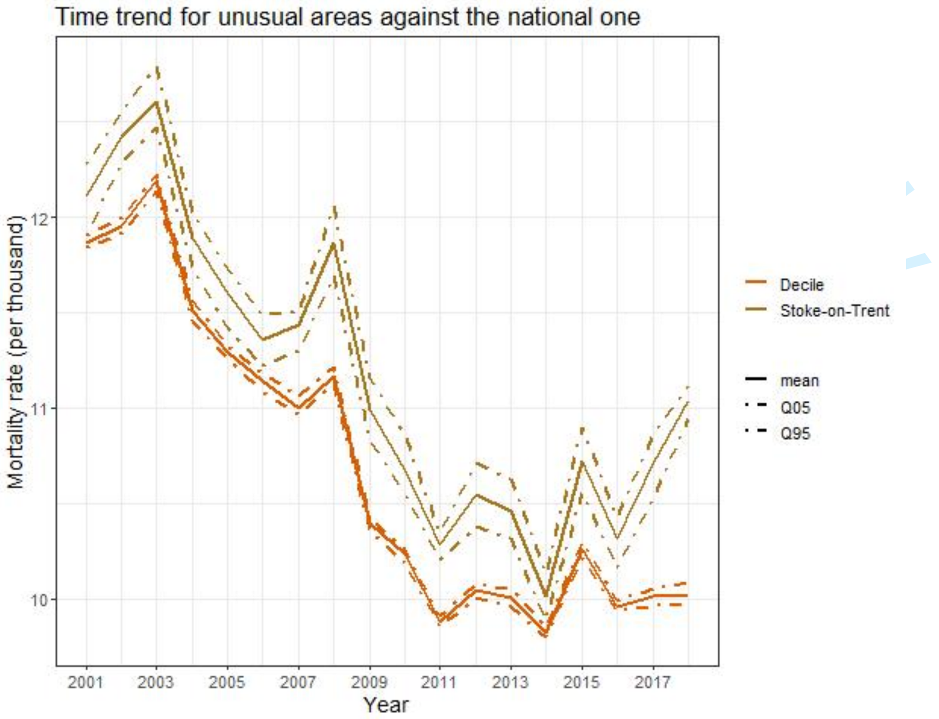

Figure S3: Tameside, females

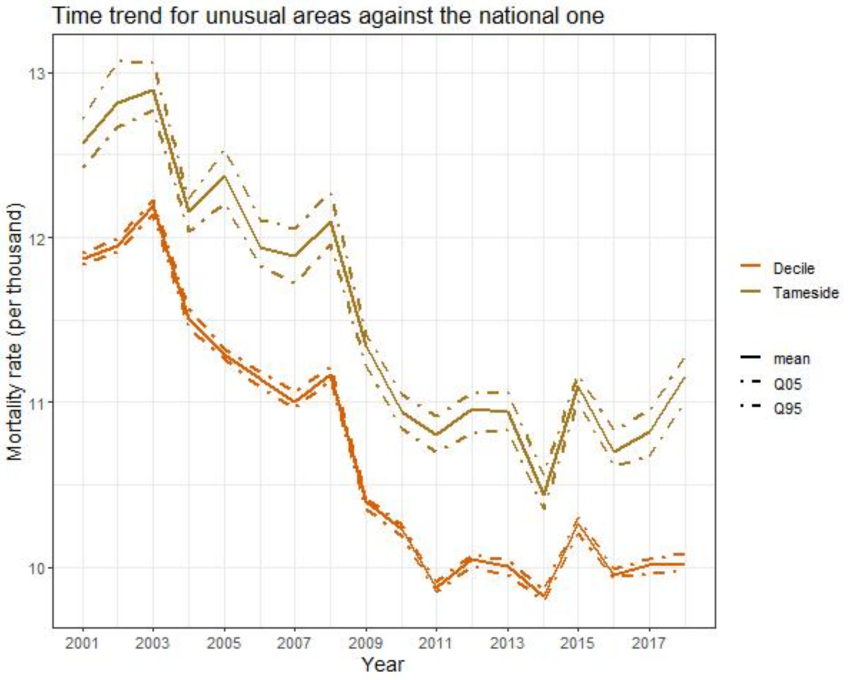

Figure S4: Newcastle upon Tyne, females

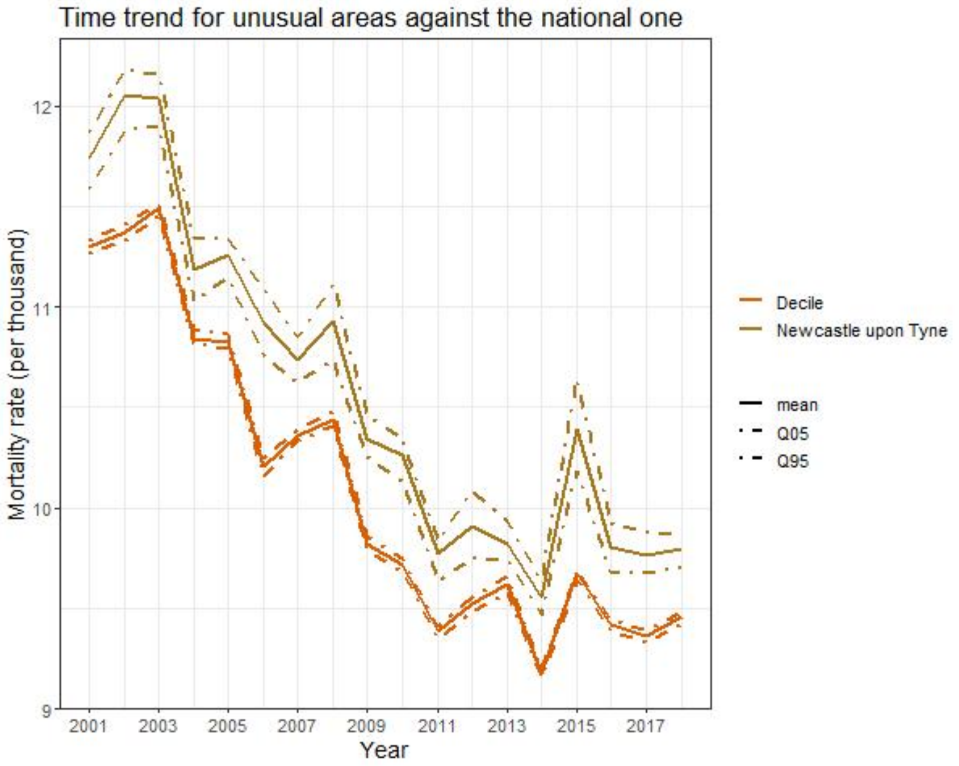

Figure S5: Plymouth, females

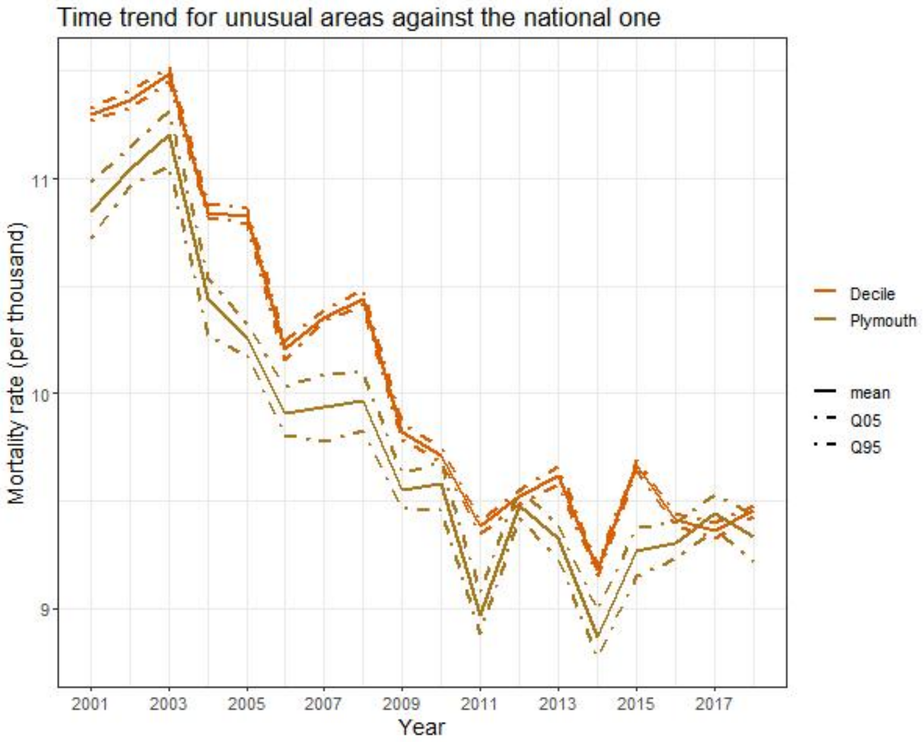

Figure S6: Leeds, females

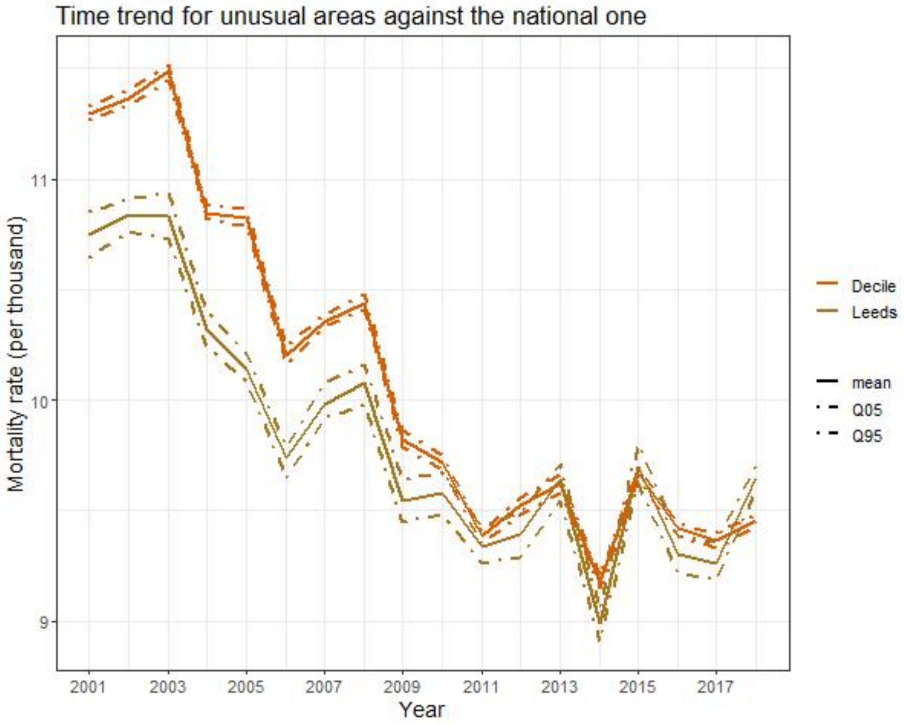

Figure S7: Derby, females

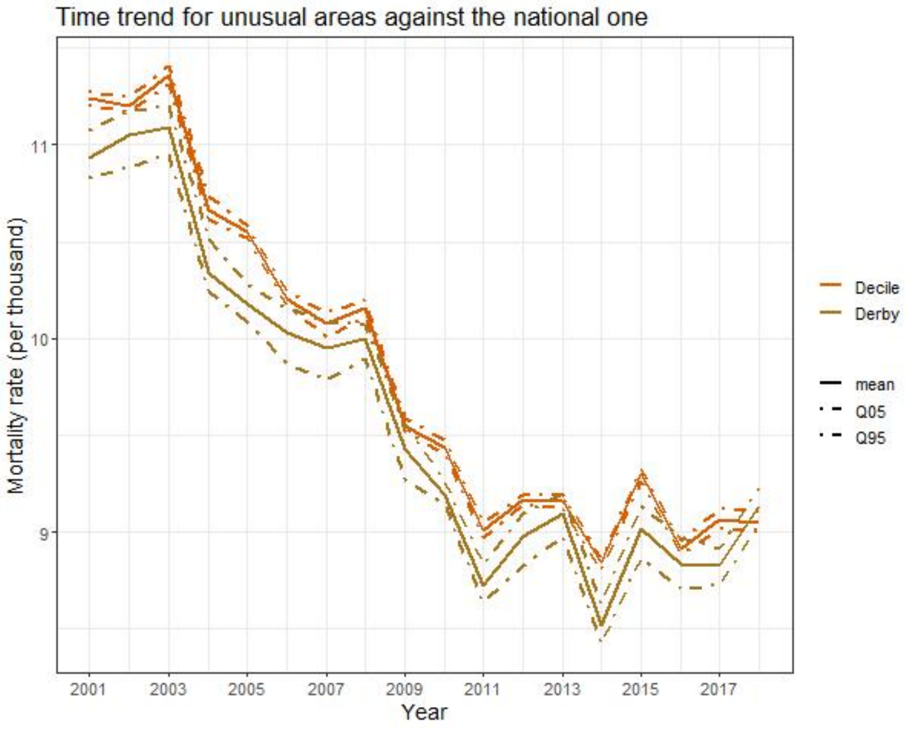

Figure S8: County Durham, females

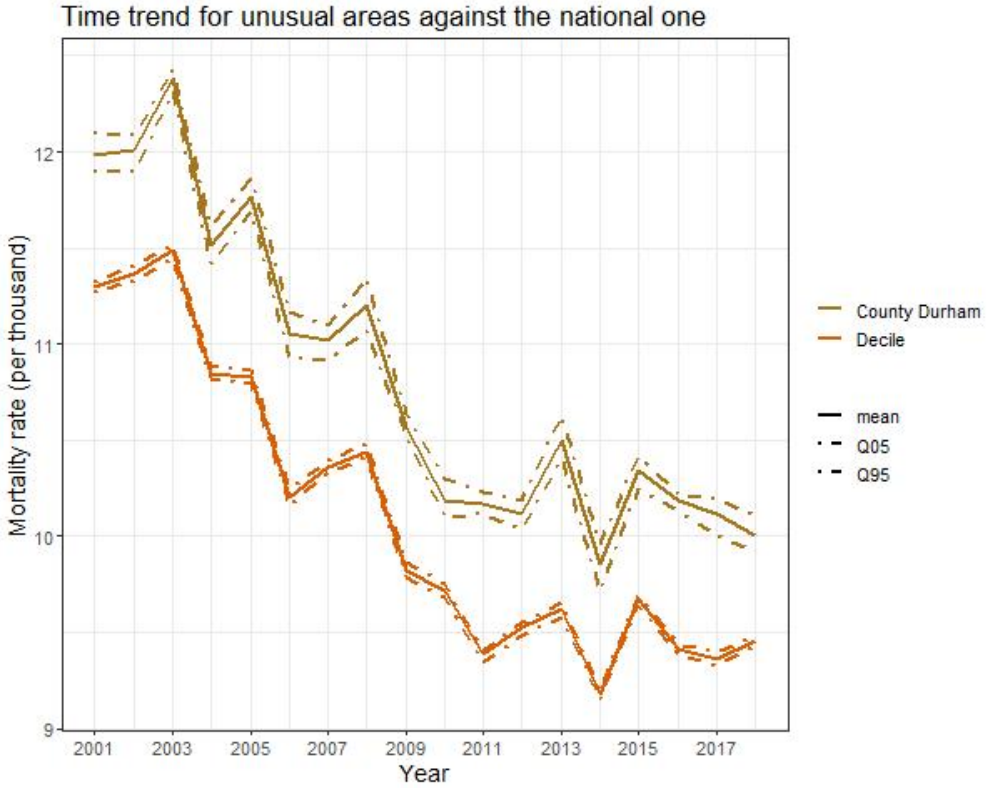

Figure S9: Cornwall including the Isles of Scilly, females

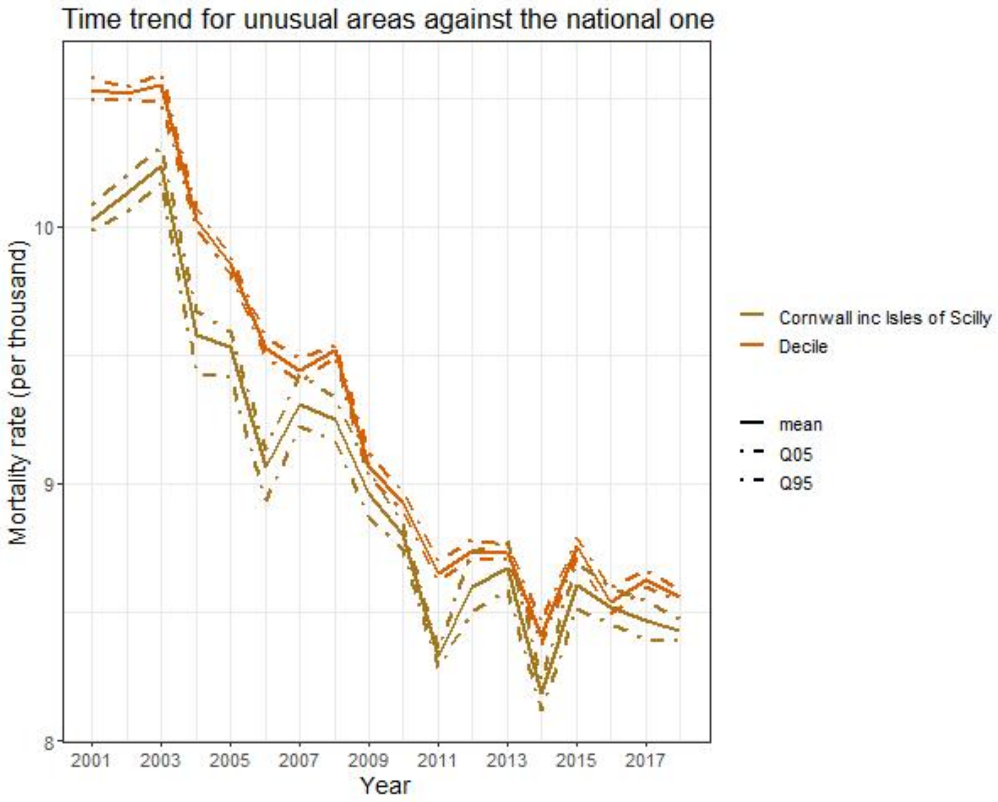

Figure S10: Bournemouth, Christchurch and Poole, Females

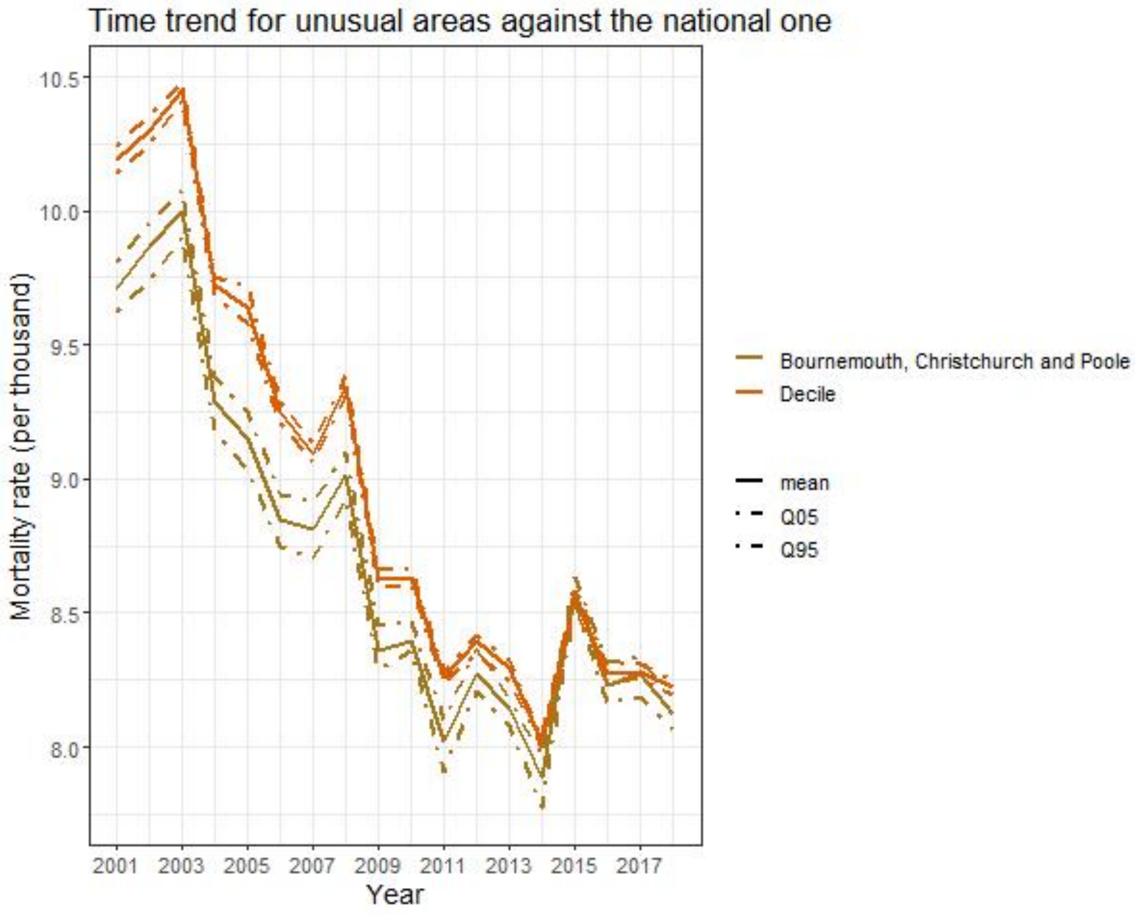

Figure S11: Stroud, females

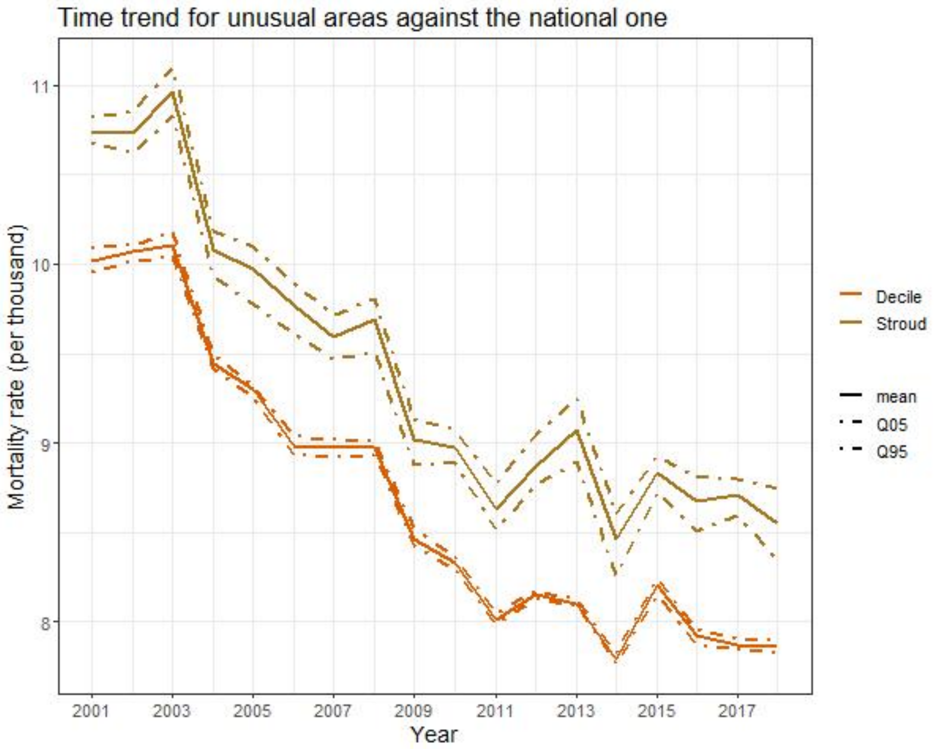

Figure S12: Sutton, females

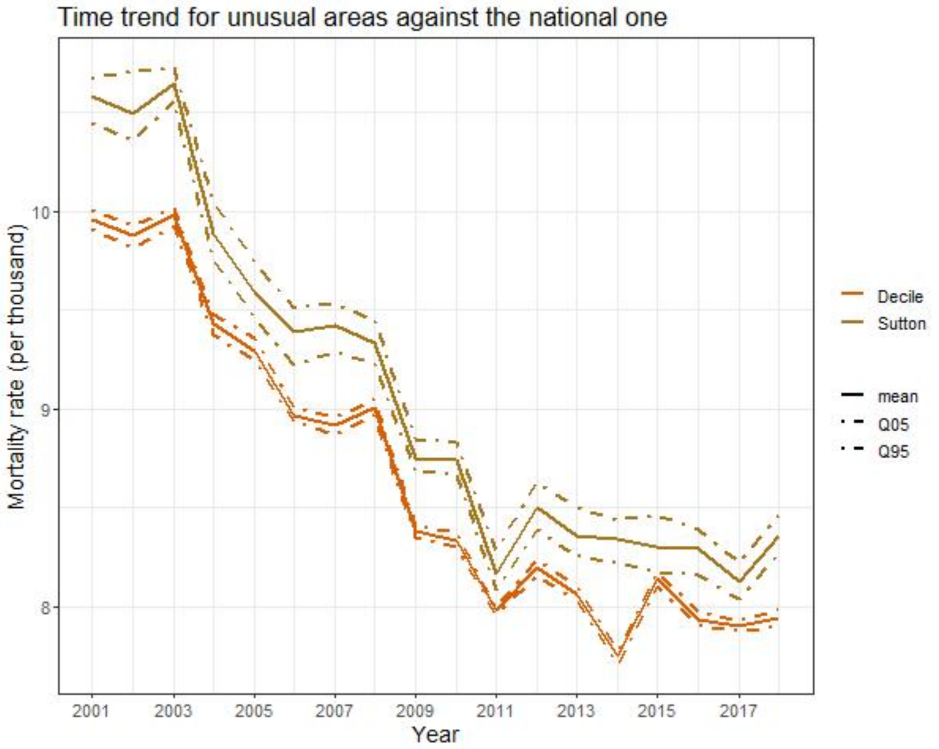

Mortality rates for the detected areas under the main model and comparison against the corresponding decile (IMD2019), males

Figure S13: Middlesbrough, males

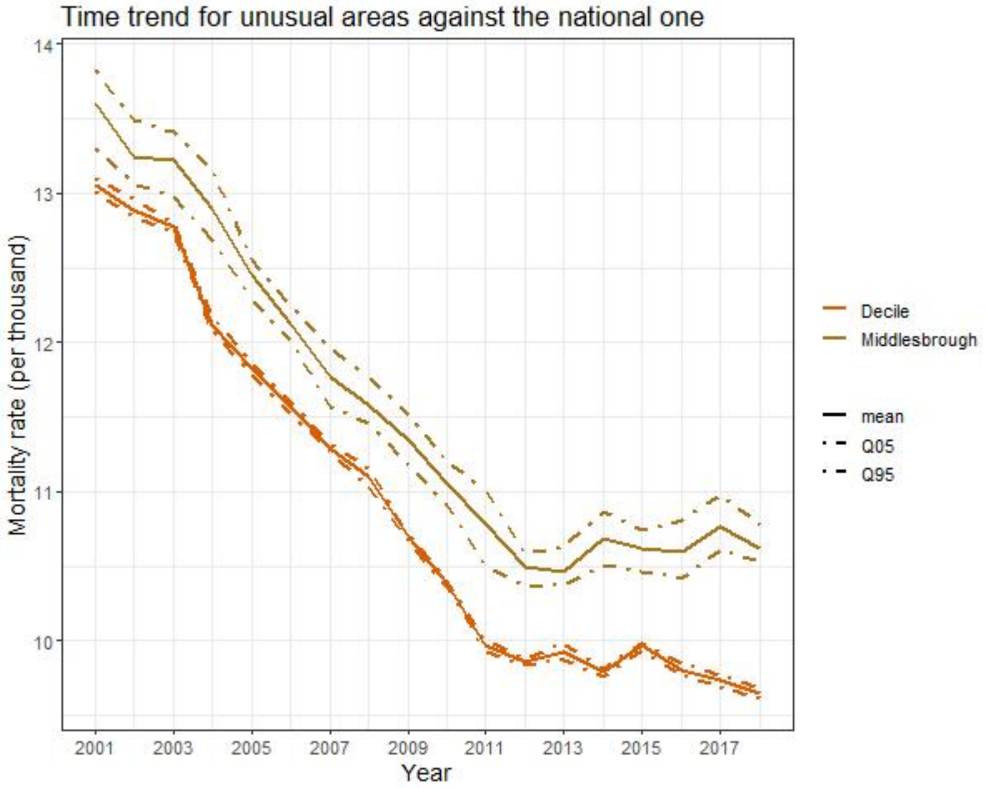

Figure S14: Wolverhampton, males

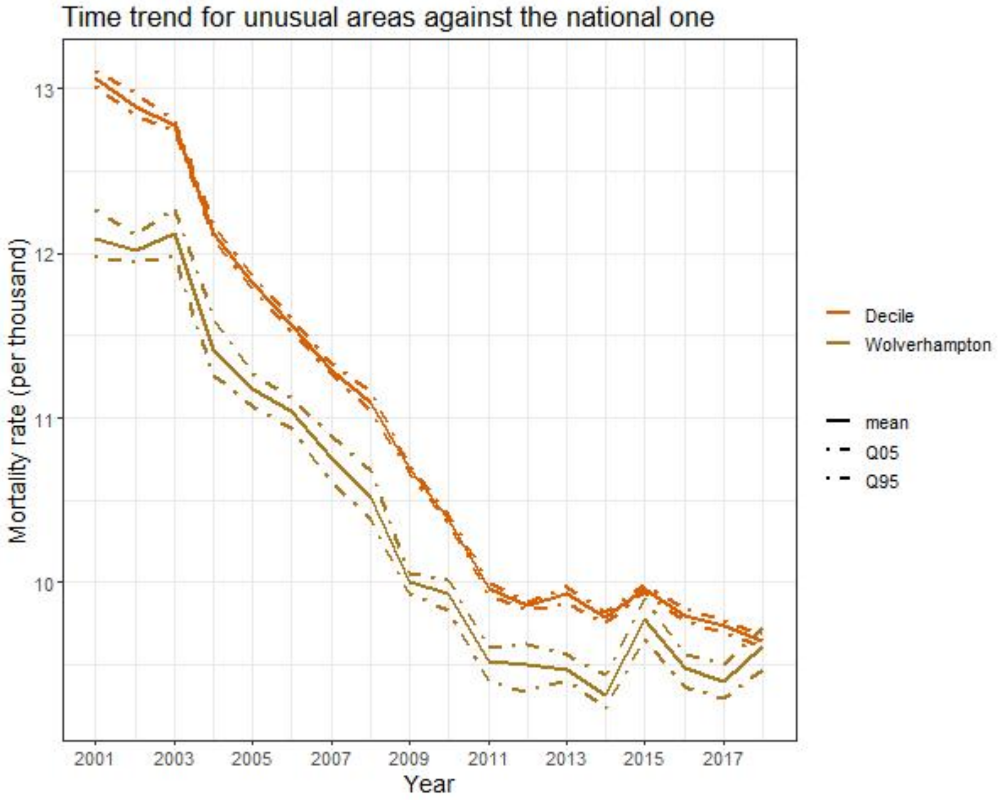

Figure S15: Kingston upon Hull, males

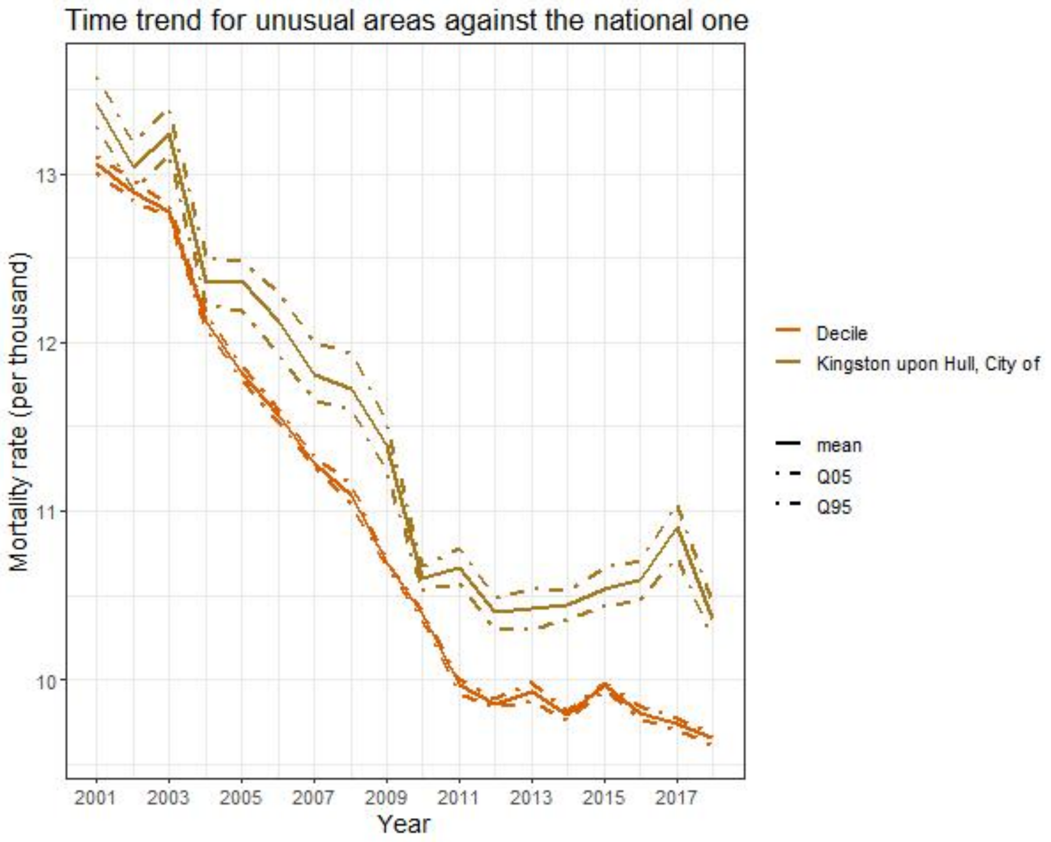

Figure S16: Birmingham, males

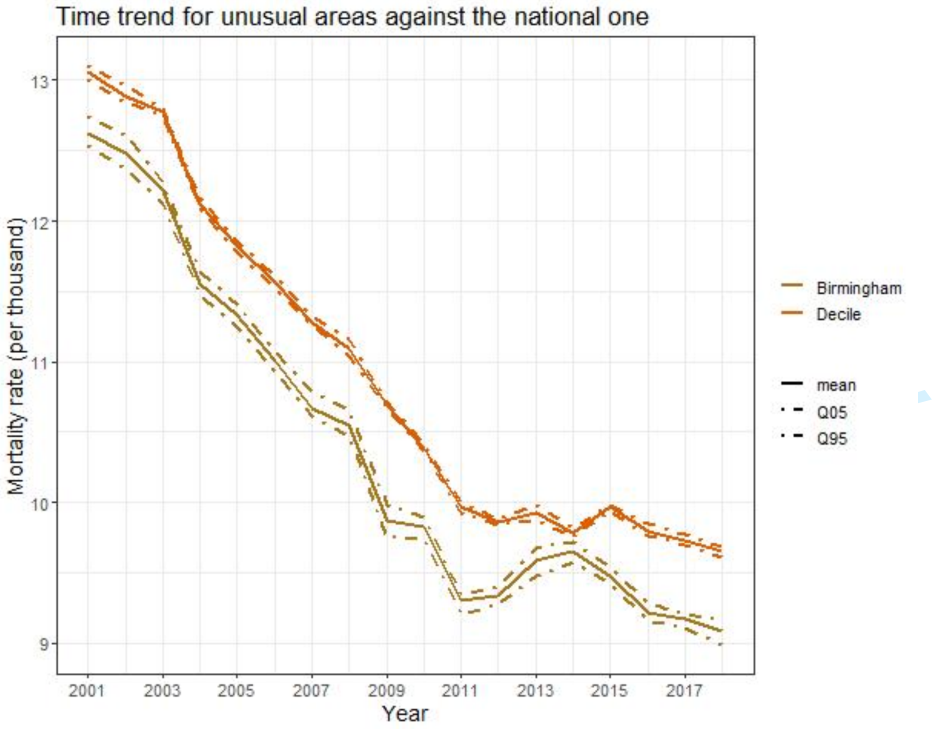

Figure S17: Tendring, males

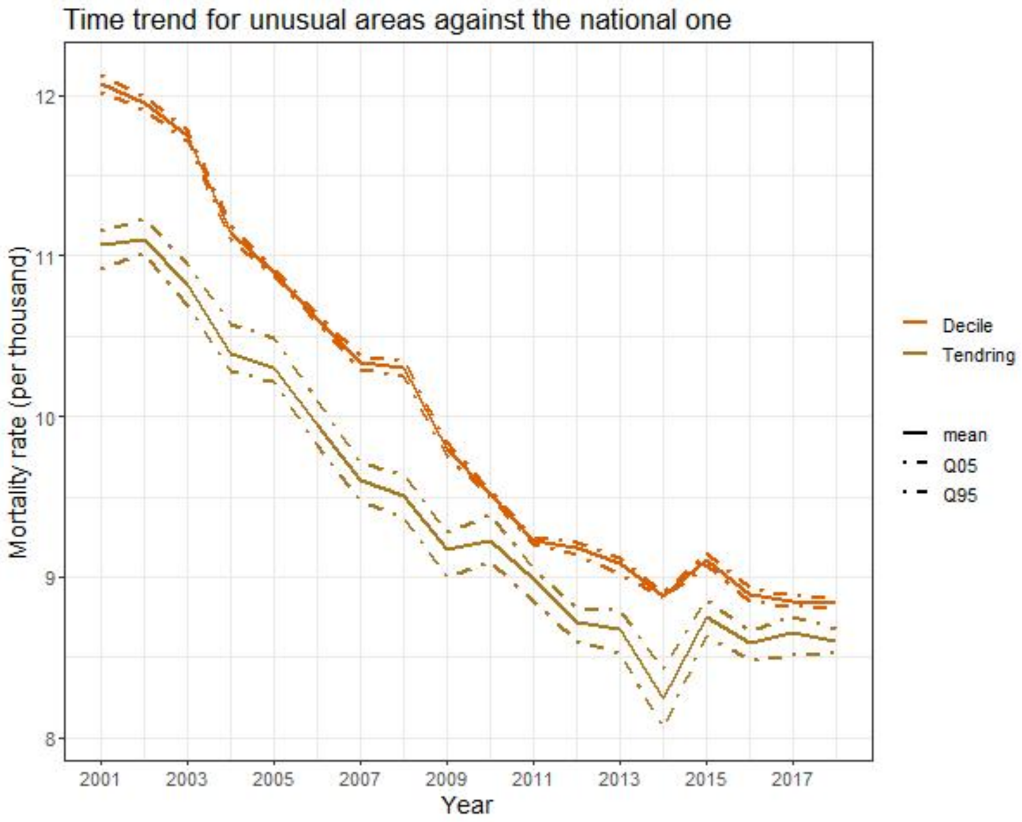

Figure S18: Leeds, males

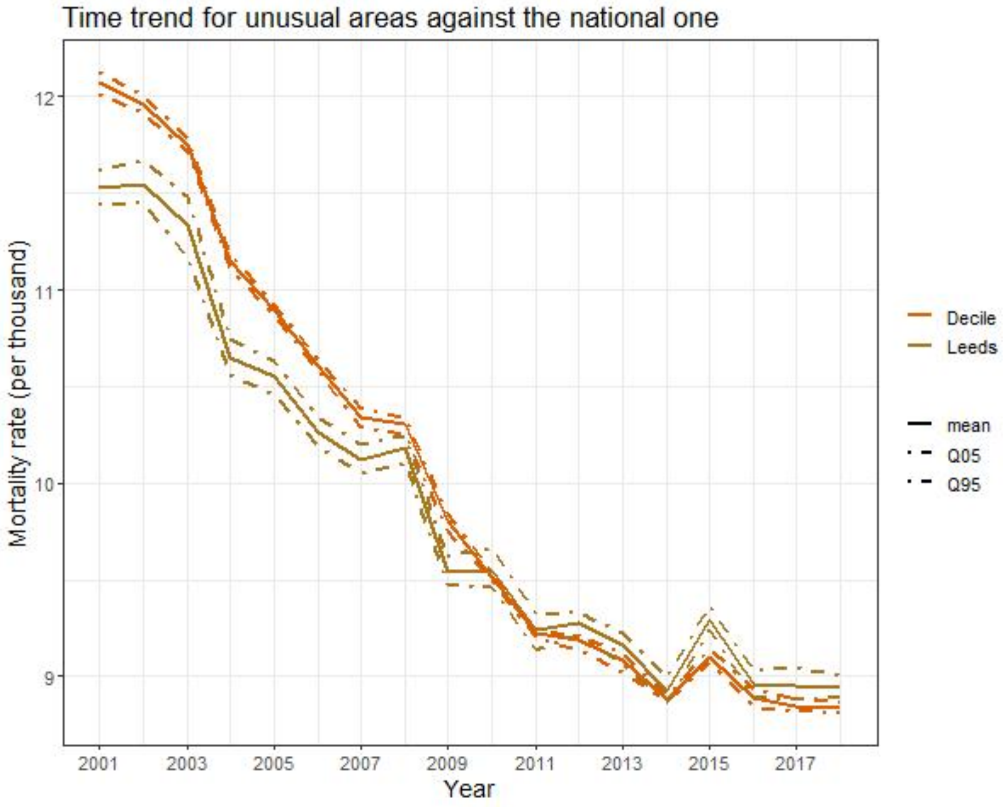

Figure S19: Newcastle upon Tyne, males

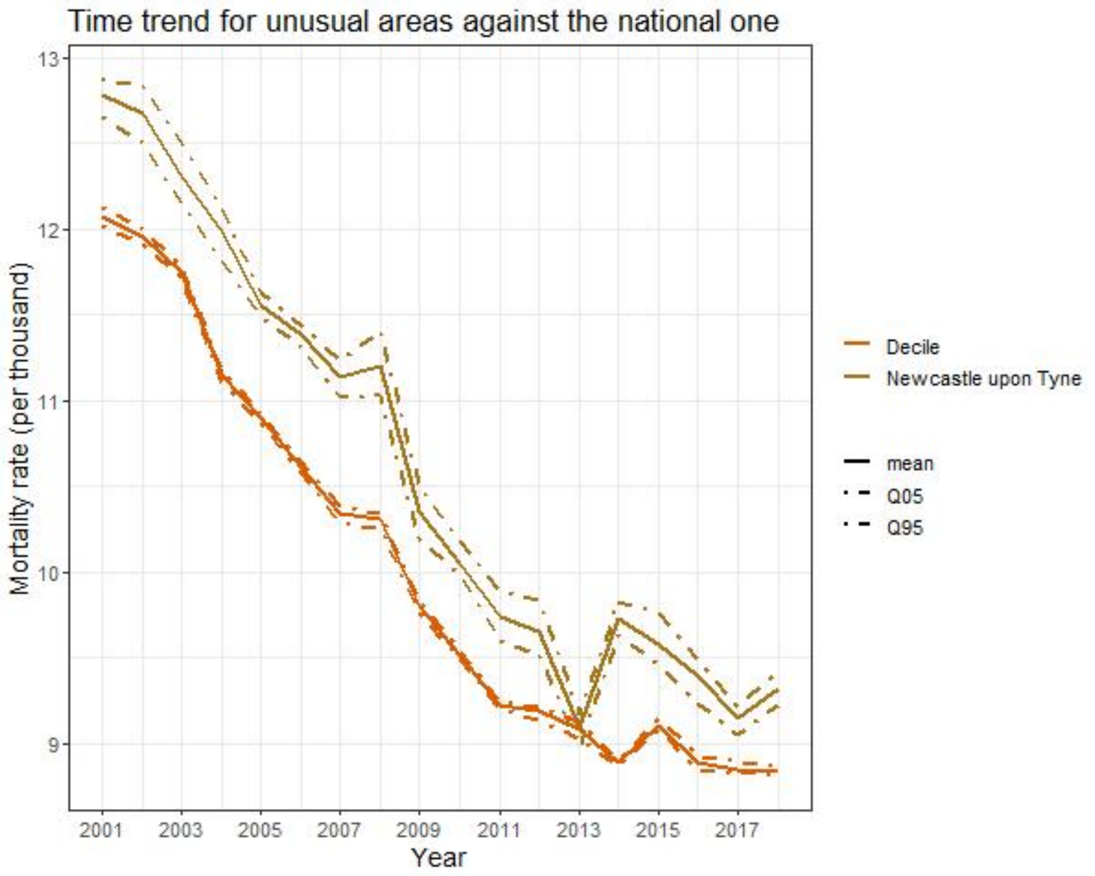

Figure S20: Southampton, males

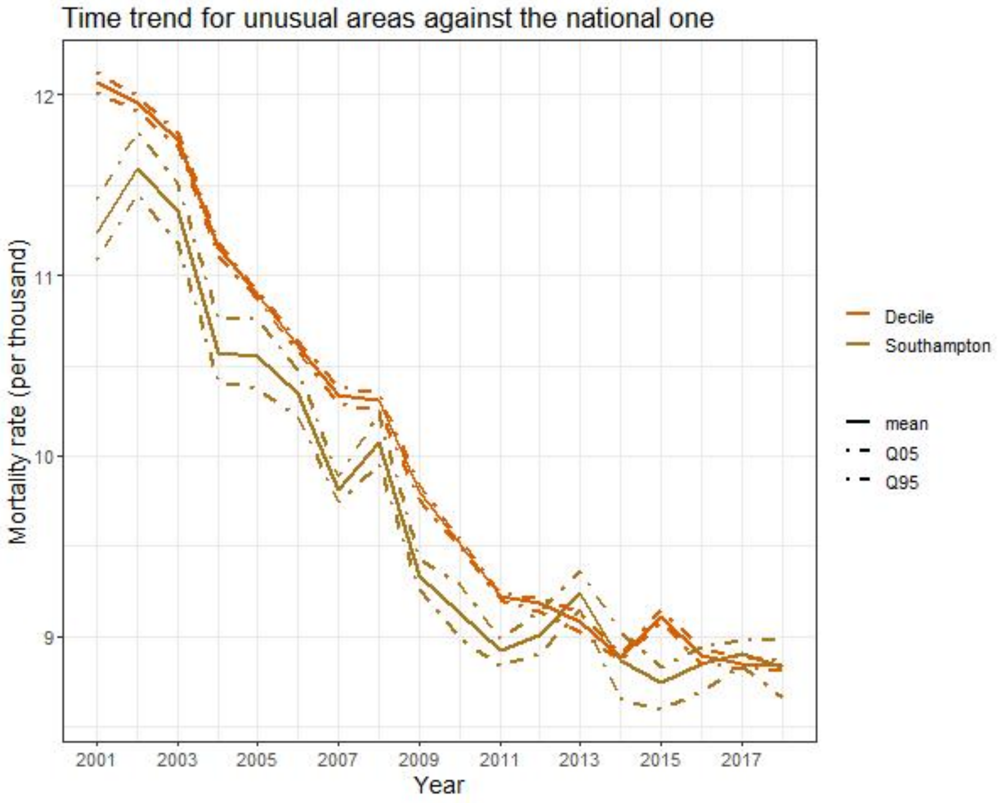

Figure S21: Bristol, males

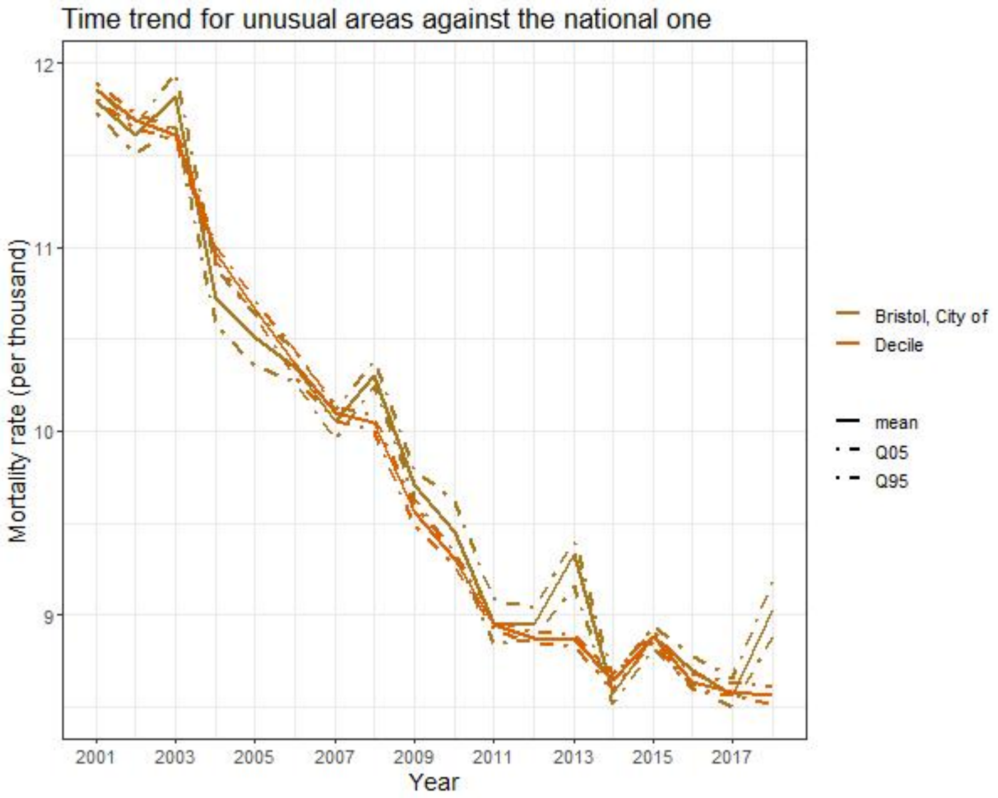

Figure S22: Cornwall including Isles of Scilly, males

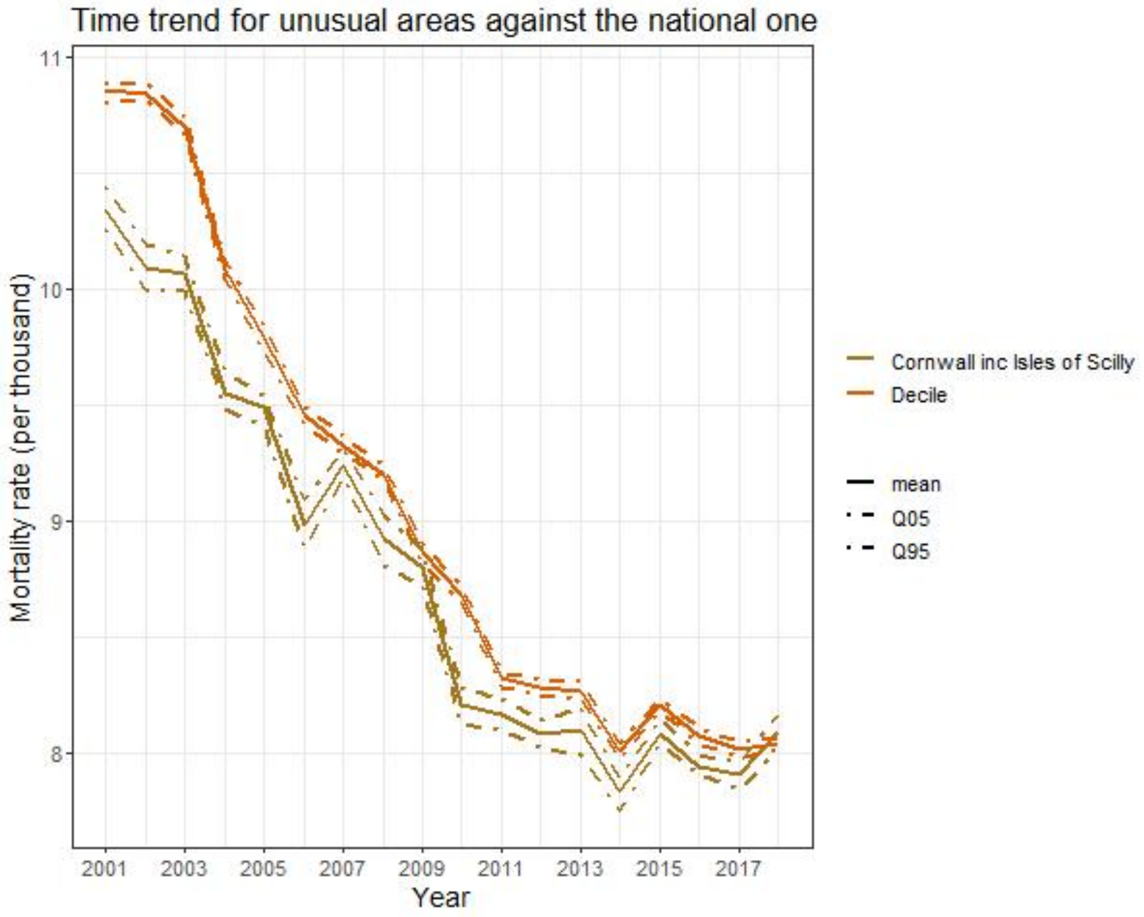

Figure S23: Breckland, males

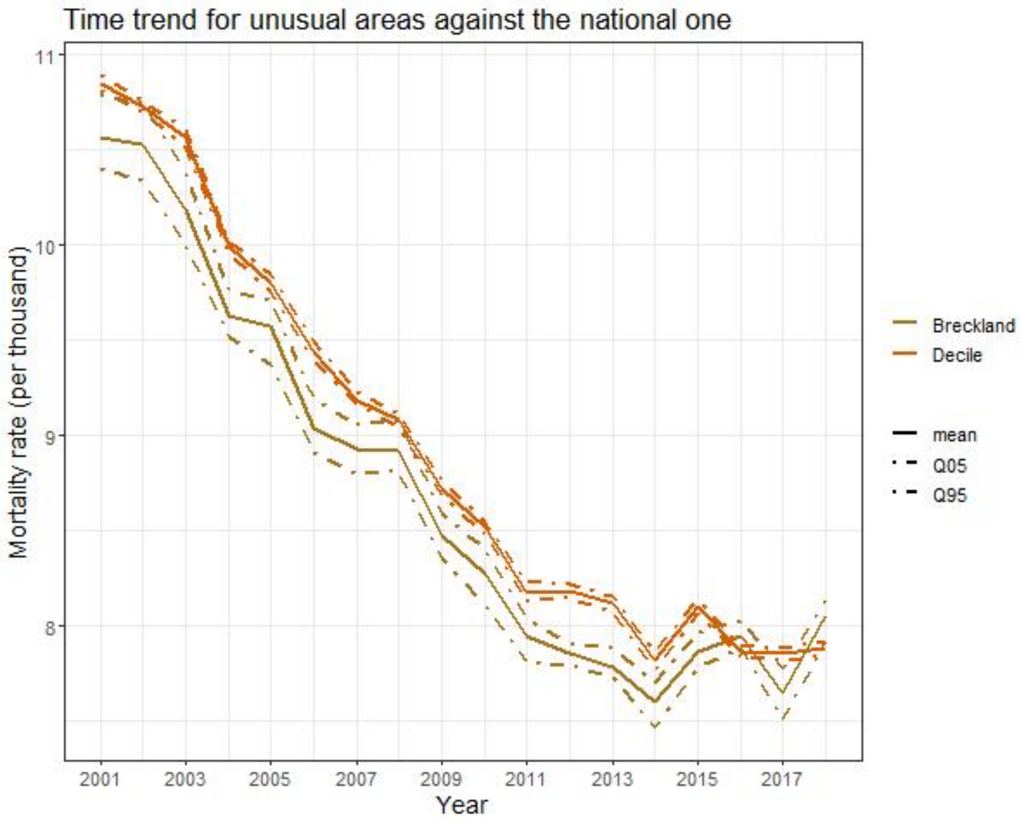

Figure S24: Ashford, males

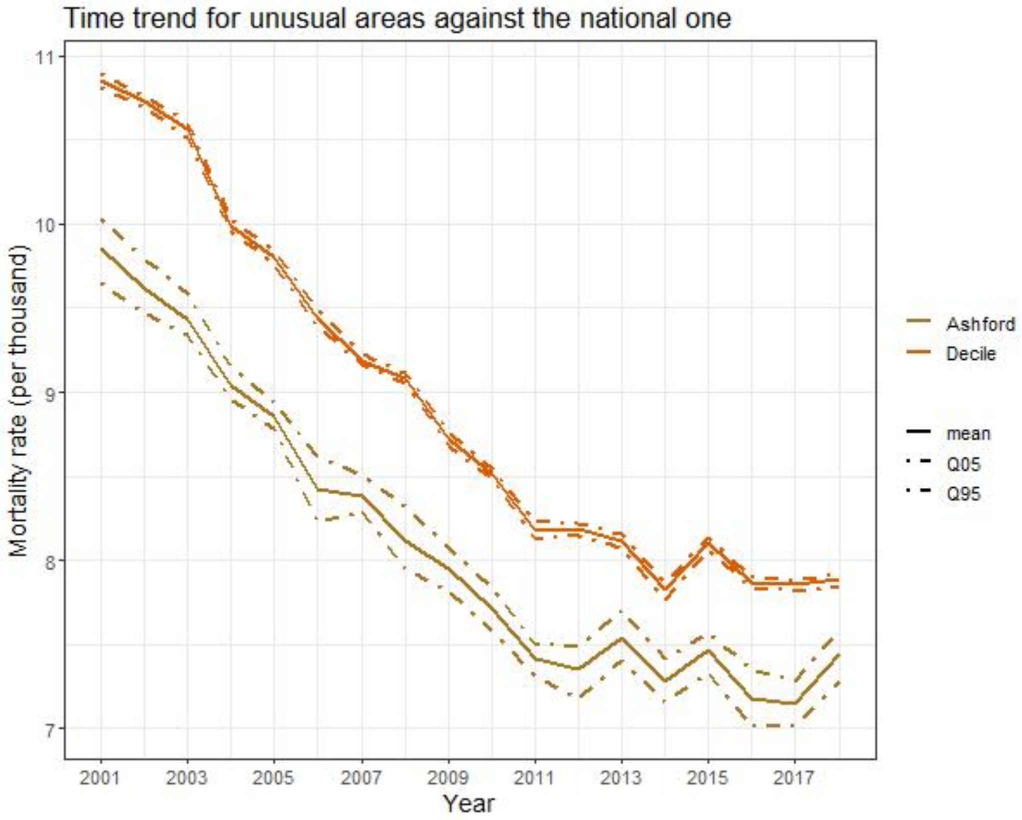

Figure S25: Hillingdon, males

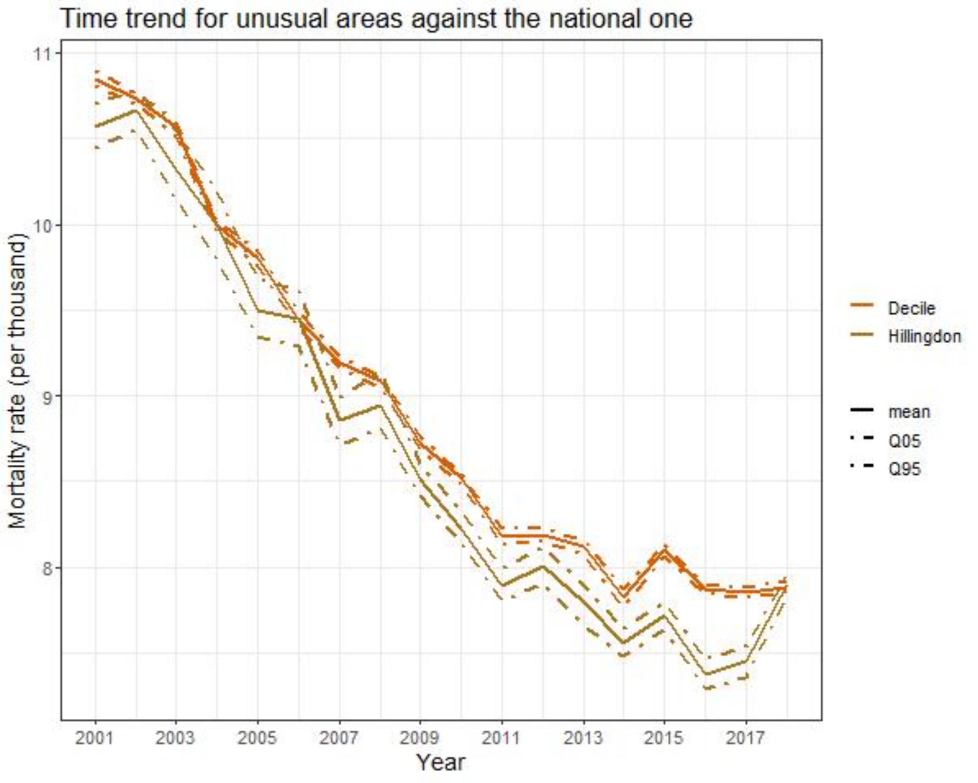

Figure S26: Cheshire West and Chester, males

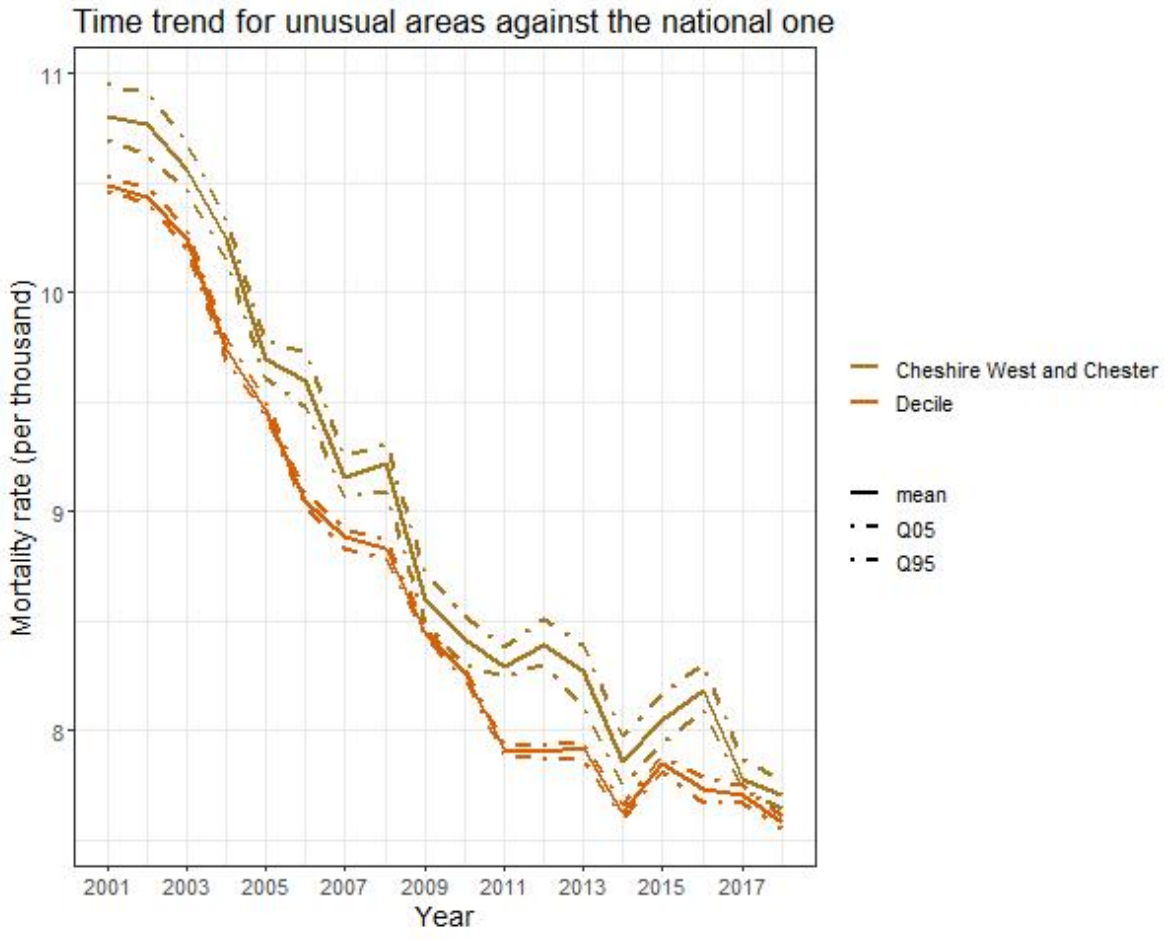

Figure S27: Dorset council, males

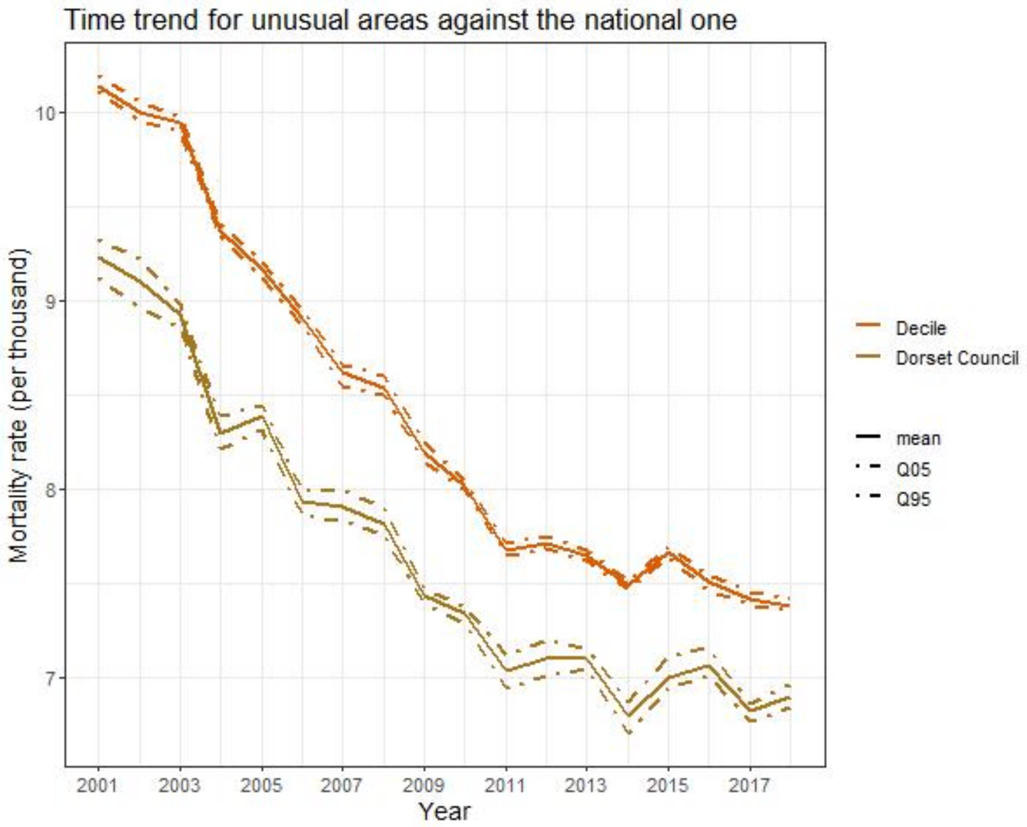

Figure S28: Teignbridge, males

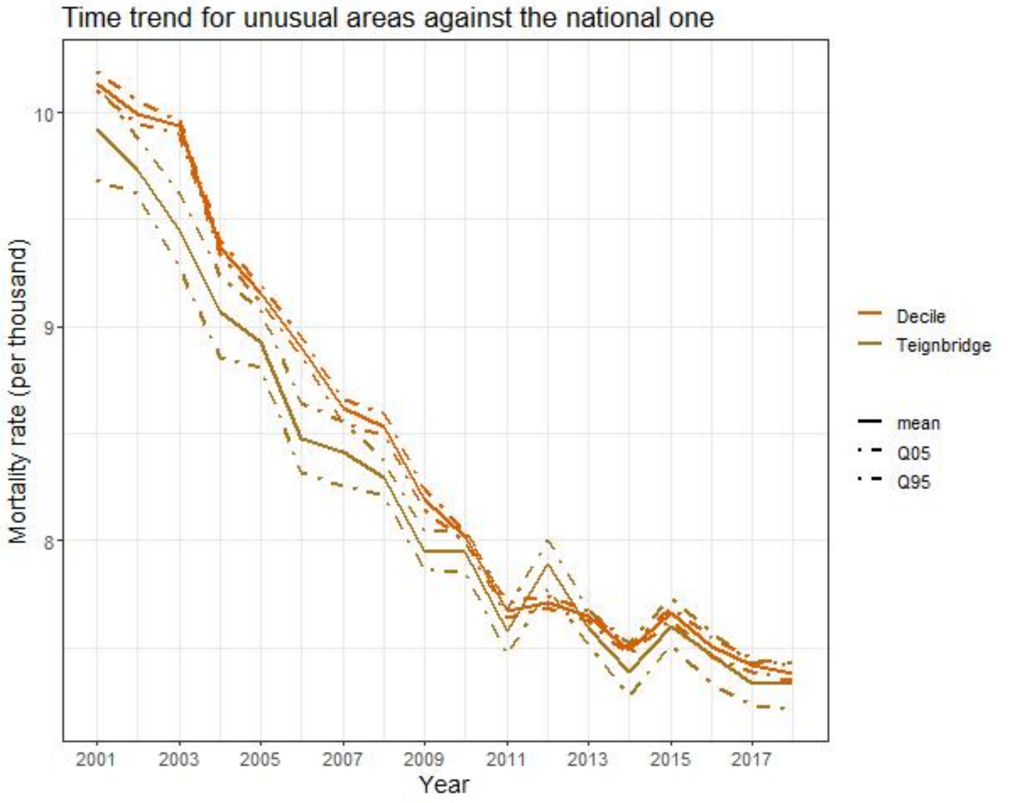

Figure S29: Stroud, males

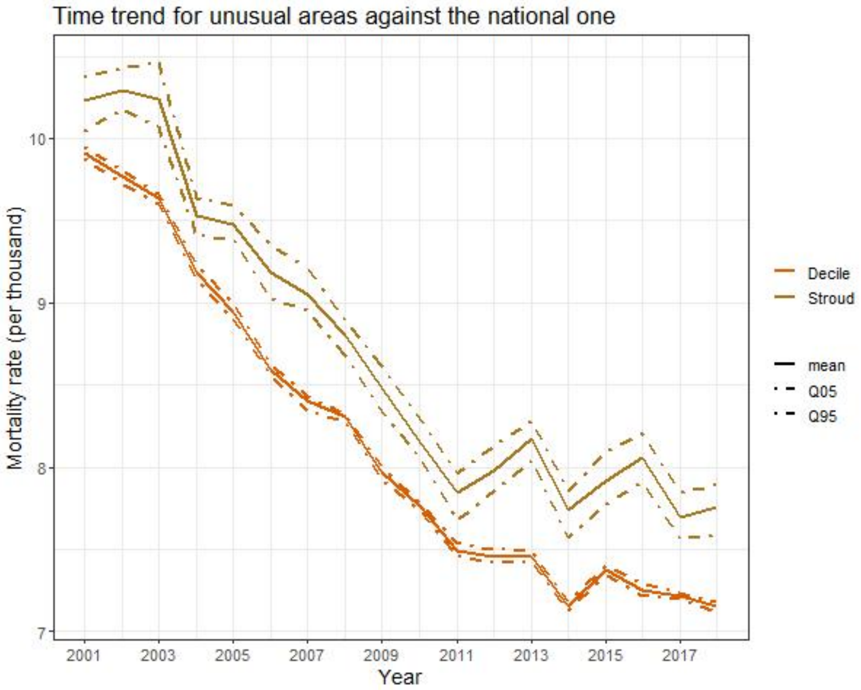

Figure S30: Bath and North East Somerset, males

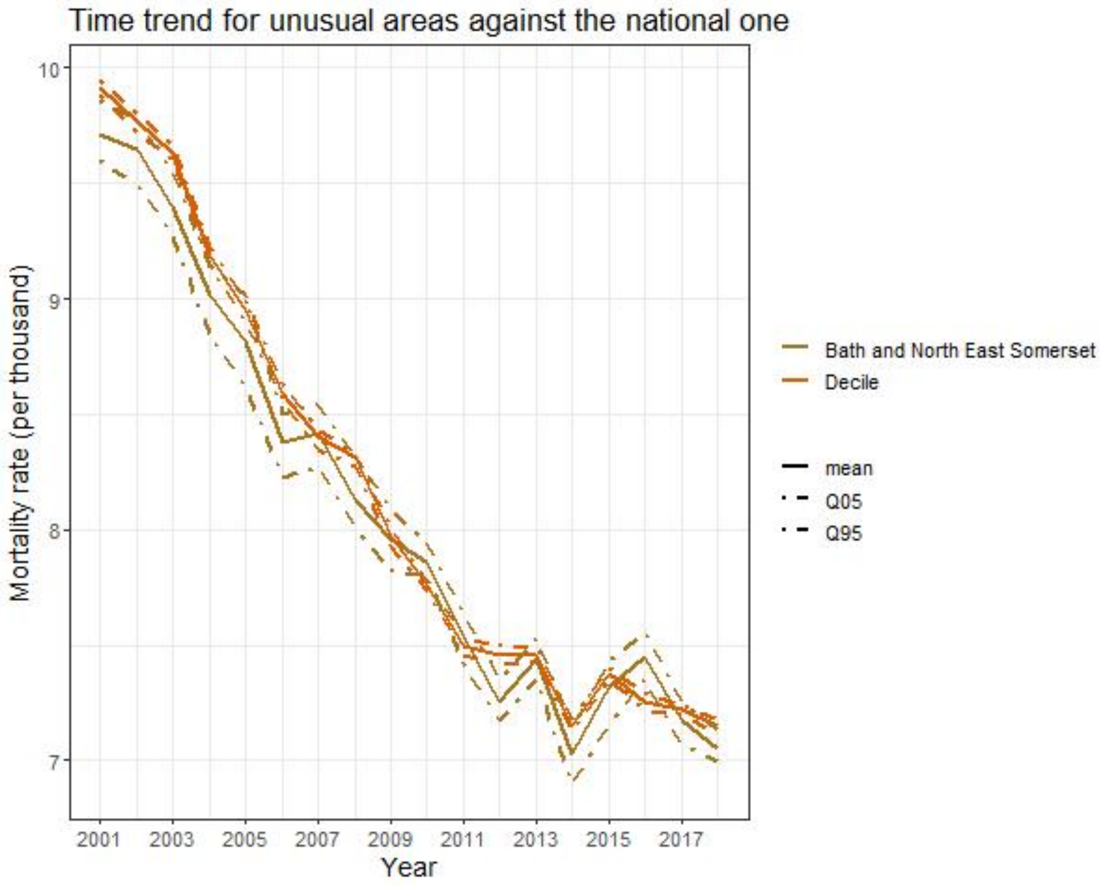

Supplement: Author's manuscript [file bmjopen-2020-036855.draft_revisions.pdf]
